# Supplementary material for: Integrated omics analysis reveals the immunologic characteristics of cystic Peyer’s patches in the cecum of Bactrian camels
Source: PeerJ. 2023 Jan 9;11:e14647. doi: 10.7717/peerj.14647 (PMC9835693; doi:10.7717/peerj.14647)
Supplement: Table S1 — The specific immune system-related pathways in this study contained the detected intestinal immune network for IgA production, theTh1/Th2/Th17 cell differentiation, the T cell receptor signaling pathway, and the antigen processing and presentation. [file peerj-11-14647-s001.docx]

Table S1. Differential expressed genes enriched in the specific immune-related pathways by KEGG enrichment analysis.

| Gene ID | | Gene symbol | Up- or down-regulated |
| --- | --- | --- | --- |
| 1. **IgA production** | | | |
| ncbi_105061813 | | CCR9 | Up |
| ncbi_105062175 | | AICDA | Up |
| ncbi_105062879 | | TGFB1 | Up |
| ncbi_105064235 | | HLA-DOA | Up |
| ncbi_105064238 | | HLA-DMA | Up |
| ncbi_105064239 | | HLA-DMB | Up |
| ncbi_105064245 | | HLA-DOB | Up |
| ncbi_105064246 | | HLA-DRB1 | Up |
| ncbi_105064247 | | HLA-DRB1 | Up |
| ncbi_105064248 | | HLA-DQA2 | Up |
| ncbi_105064249 | | HLA-DQB1 | Up |
| ncbi_105064250 | | HLA-DRA | Up |
| ncbi_105064276 | | HLA-DRB1 | Up |
| ncbi_105066901 | | TNFSF13B | Up |
| ncbi_105067053 | | CD40LG | Up |
| ncbi_105071211 | | LTBR | Down |
| ncbi_105071882 | | CD40 | Up |
| ncbi_105072525 | | MAP3K14 | Up |
| ncbi_105072900 | | TRBV25-1 | Up |
| ncbi_105072984 | | ITGA4 | Up |
| ncbi_105073193 | | TNFRSF13C | Up |
| ncbi_105074241 | | TNFRSF17 | Up |
| ncbi_105074341 | | IL15RA | Down |
| ncbi_105075425 | | IL10 | Up |
| ncbi_105076376 | | ITGB7 | Up |
| ncbi_105077504 | | TNFSF13 | Down |
| ncbi_105078061 | | ICOSLG | Up |
| ncbi_105078099 | | Madcam1 | Up |
| ncbi_105078293 | | CCL25 | Up |
| ncbi_105078516 | | CXCL12 | Up |
| ncbi_105079801 | | IL6 | Up |
| ncbi_105081591 | | CD80 | Up |
| ncbi_105081617 | | CD86 | Up |
| ncbi_105081933 | | TNFRSF13B | Up |
| ncbi_105082277 | | ICOS | Up |
| ncbi_105082279 | | CD28 | Up |
| ncbi_105083390 | | CXCR4 | Up |
| MSTRG.11100 | | TRAV16 | Up |
| MSTRG.11101 | | TRAV18 | Up |
| MSTRG.11102 | | TRAV18 | Up |
| MSTRG.11103 | | TRAV8-4 | Up |
| MSTRG.14532 | | IGHG1 | Up |
| MSTRG.14534 | | IGHG2 | Up |
| MSTRG.2847 | | TRBV16 | Up |
| MSTRG.2850 | | TRBV14 | Up |
| MSTRG.2852 | | TRBV10-3 | Up |
| MSTRG.2853 | | TRBV7-9 | Up |
| MSTRG.2854 | | TRBV5-5 | Up |
| MSTRG.2855 | | V-TCR | Up |
| MSTRG.2857 | | TRBV12-4 | Up |
| MSTRG.2858 | | TRBV3-1 | Up |
| MSTRG.4011 | | TRAV9-2 | Up |
| MSTRG.4012 | | TRAV14DV4 | Up |
| MSTRG.4014 | | TRAV9-2 | Up |
| MSTRG.4016 | | TRAV8-3 | Up |
| MSTRG.4017 | | TRAV9-1 | Up |
| MSTRG.4019 | | TRAV2 | Up |
| MSTRG.6776 | | TRDV1 | Up |
| MSTRG.6780 | | TRAV23DV6 | Up |
| MSTRG.6781 | | TRDV1 | Up |
| MSTRG.6787 | | TRA | Up |
| MSTRG.84 | | TRB | Up |
| MSTRG.85 | | TRB | Up |
| MSTRG.86 | | TRB | Up |
| MSTRG.87 | | TRBV29-1 | Up |
| MSTRG.89 | | TRBV27 | Up |
| MSTRG.2850 | | TRBV14 | Up |
| MSTRG.2852 | | TRBV10-3 | Up |
| MSTRG.2853 | | TRBV7-9 | Up |
| MSTRG.2854 | | TRBV5-5 | Up |
| MSTRG.2855 | | V-TCR | Up |
| MSTRG.2857 | | TRBV12-4 | Up |
| MSTRG.2858 | | TRBV3-1 | Up |
| MSTRG.4011 | | TRAV9-2 | Up |
| MSTRG.4012 | | TRAV14DV4 | Up |
| MSTRG.4014 | | TRAV9-2 | Up |
| MSTRG.4016 | | TRAV8-3 | Up |
| MSTRG.4017 | | TRAV9-1 | Up |
| MSTRG.4019 | | TRAV2 | Up |
| MSTRG.6776 | | TRDV1 | Up |
| MSTRG.6780 | | TRAV23DV6 | Up |
| MSTRG.6781 | | TRDV1 | Up |
| MSTRG.6787 | | TRA | Up |
| MSTRG.84 | | TRB | Up |
| MSTRG.85 | | TRB | Up |
| MSTRG.86 | | TRB | Up |
| MSTRG.87 | | TRBV29-1 | Up |
| MSTRG.89 | | TRBV27 | Up |
| 1. **Th1/Th2/Th17 cell differentiation** | | | |
| ncbi_105062116 | STAT6 | Up |  |
| ncbi_105063497 | RUNX3 | Up |  |
| ncbi_105063980 | STAT4 | Up |  |
| ncbi_105063982 | STAT1 | Up |  |
| ncbi_105064175 | MAPK13 | Down |  |
| ncbi_105064235 | HLA-DOA | Up |  |
| ncbi_105064238 | HLA-DMA | Up |  |
| ncbi_105064239 | HLA-DMB | Up |  |
| ncbi_105064245 | HLA-DOB | Up |  |
| ncbi_105064246 | HLA-DRB1 | Up |  |
| ncbi_105064247 | HLA-DRB1 | Up |  |
| ncbi_105064248 | HLA-DQA2 | Up |  |
| ncbi_105064249 | HLA-DQB1 | Up |  |
| ncbi_105064250 | HLA-DRA | Up |  |
| ncbi_105064276 | HLA-DRB1 | Up |  |
| ncbi_105064618 | JAK1 | Up |  |
| ncbi_105064951 | JAG1 | Up |  |
| ncbi_105066646 | IL12A | Up |  |
| ncbi_105066766 | LCK | Up |  |
| ncbi_105067817 | CD3G | Up |  |
| ncbi_105067818 | CD3D | Up |  |
| ncbi_105067821 | CD3E | Up |  |
| ncbi_105067906 | PPP3CA | Up |  |
| ncbi_105067913 | NFKB1 | Up |  |
| ncbi_105068057 | CD247 | Up |  |
| ncbi_105068381 | TYK2 | Up |  |
| ncbi_105069046 | NFATC1 | Up |  |
| ncbi_105069349 | MAPK1 | Up |  |
| ncbi_105069770 | NOTCH1 | Up |  |
| ncbi_105070806 | MAPK12 | Up |  |
| ncbi_105070807 | MAPK11 | Up |  |
| ncbi_105071091 | CD4 | Up |  |
| ncbi_105071245 | IKBKB | Up |  |
| ncbi_105071674 | JUN | Down |  |
| ncbi_105071843 | NFATC2 | Up |  |
| ncbi_105071908 | RBPJL | Down |  |
| ncbi_105071939 | PLCG1 | Up |  |
| ncbi_105072189 | ZAP70 | Up |  |
| ncbi_105072900 | TRBV25-1 | Up |  |
| ncbi_105073156 | IL2RB | Up |  |
| ncbi_105073341 | TBX21 | Up |  |
| ncbi_105074228 | GATA3 | Up |  |
| ncbi_105074303 | PRKCQ | Up |  |
| ncbi_105074562 | NOTCH3 | Up |  |
| ncbi_105074697 | IL2RA | Up |  |
| ncbi_105074856 | NFATC3 | Up |  |
| ncbi_105075281 | RBPJ | Up |  |
| ncbi_105075289 | FOS | Down |  |
| ncbi_105077267 | IFNGR1 | Up |  |
| ncbi_105077723 | MAPK10 | Up |  |
| ncbi_105078829 | V-MAF | Up |  |
| ncbi_105079173 | JAK2 | Up |  |
| ncbi_105079218 | NOTCH2 | Up |  |
| ncbi_105080031 | JAG2 | Up |  |
| ncbi_105080117 | STAT5A | Up |  |
| ncbi_105080118 | STAT5B | Up |  |
| ncbi_105082560 | JAK3 | Up |  |
| ncbi_105082567 | IL12RB1 | Up |  |
| ncbi_105082839 | Mapk3 | Down |  |
| ncbi_105082855 | LAT | Up |  |
| ncbi_105083483 | MAML2 | Up |  |
| ncbi_105083690 | IL2RG | Up |  |
| MSTRG.11100 | TRAV16 | Up |  |
| MSTRG.11101 | TRAV18 | Up |  |
| MSTRG.11102 | TRAV18 | Up |  |
| MSTRG.11103 | TRAV8-4 | Up |  |
| MSTRG.16869 | MAML2 | Down |  |
| MSTRG.2847 | TRBV16 | Up |  |
| MSTRG.2850 | TRBV14 | Up |  |
| MSTRG.2852 | TRBV10-3 | Up |  |
| MSTRG.2853 | TRBV7-9 | Up |  |
| MSTRG.2854 | TRBV5-5 | Up |  |
| MSTRG.2855 | V-TCR | Up |  |
| MSTRG.2857 | TRBV12-4 | Up |  |
| MSTRG.2858 | TRBV3-1 | Up |  |
| MSTRG.4011 | TRAV9-2 | Up |  |
| MSTRG.4012 | TRAV14DV4 | Up |  |
| MSTRG.4014 | TRAV9-2 | Up |  |
| MSTRG.4016 | TRAV8-3 | Up |  |
| MSTRG.4017 | TRAV9-1 | Up |  |
| MSTRG.4019 | TRAV2 | Up |  |
| MSTRG.6776 | TRDV1 | Up |  |
| MSTRG.6780 | TRAV23DV6 | Up |  |
| MSTRG.6781 | TRDV1 | Up |  |
| MSTRG.6787 | TRA | Up |  |
| MSTRG.84 | TRB | Up |  |
| MSTRG.85 | TRB | Up |  |
| MSTRG.86 | TRB | Up |  |
| MSTRG.87 | TRBV29-1 | Up |  |
| MSTRG.89 | TRBV27 | Up |  |
| ncbi_105062116 | STAT6 | Up |  |
| ncbi_105062879 | TGFB1 | Up |  |
| ncbi_105063982 | STAT1 | Up |  |
| ncbi_105064175 | MAPK13 | Down |  |
| ncbi_105064235 | HLA-DOA | Up |  |
| ncbi_105064238 | HLA-DMA | Up |  |
| ncbi_105064239 | HLA-DMB | Up |  |
| ncbi_105064245 | HLA-DOB | Up |  |
| ncbi_105064246 | HLA-DRB1 | Up |  |
| ncbi_105064247 | HLA-DRB1 | Up |  |
| ncbi_105064248 | HLA-DQA2 | Up |  |
| ncbi_105064249 | HLA-DQB1 | Up |  |
| ncbi_105064250 | HLA-DRA | Up |  |
| ncbi_105064276 | HLA-DRB1 | Up |  |
| ncbi_105064618 | JAK1 | Up |  |
| ncbi_105066766 | LCK | Up |  |
| ncbi_105067114 | IL21 | Up |  |
| ncbi_105067391 | TGFBR1 | Up |  |
| ncbi_105067817 | CD3G | Up |  |
| ncbi_105067818 | CD3D | Up |  |
| ncbi_105067821 | CD3E | Up |  |
| ncbi_105067906 | PPP3CA | Up |  |
| ncbi_105067913 | NFKB1 | Up |  |
| ncbi_105068057 | CD247 | Up |  |
| ncbi_105068250 | IL6R | Up |  |
| ncbi_105068307 | IL27RA | Up |  |
| ncbi_105068381 | TYK2 | Up |  |
| ncbi_105068579 | RXRA | Down |  |
| ncbi_105069046 | NFATC1 | Up |  |
| ncbi_105069349 | MAPK1 | Up |  |
| ncbi_105070210 | RUNX1 | Up |  |
| ncbi_105070806 | MAPK12 | Up |  |
| ncbi_105070807 | MAPK11 | Up |  |
| ncbi_105071091 | CD4 | Up |  |
| ncbi_105071245 | IKBKB | Up |  |
| ncbi_105071674 | JUN | Down |  |
| ncbi_105071703 | RORC | Down |  |
| ncbi_105071843 | NFATC2 | Up |  |
| ncbi_105071939 | PLCG1 | Up |  |
| ncbi_105072137 | IL1B | Up |  |
| ncbi_105072189 | ZAP70 | Up |  |
| ncbi_105072217 | IL1R1 | Up |  |
| ncbi_105072636 | TGFBR2 | Up |  |
| ncbi_105072900 | TRBV25-1 | Up |  |
| ncbi_105073156 | IL2RB | Up |  |
| ncbi_105073341 | TBX21 | Up |  |
| ncbi_105073656 | IL21R | Up |  |
| ncbi_105074009 | SMAD2 | Up |  |
| ncbi_105074228 | GATA3 | Up |  |
| ncbi_105074303 | PRKCQ | Up |  |
| ncbi_105074647 | HIF1A | Up |  |
| ncbi_105074697 | IL2RA | Up |  |
| ncbi_105074856 | NFATC3 | Up |  |
| ncbi_105075289 | FOS | Down |  |
| ncbi_105075556 | IL23R | Up |  |
| ncbi_105077267 | IFNGR1 | Up |  |
| ncbi_105077723 | MAPK10 | Up |  |
| ncbi_105077802 | IL6ST | Up |  |
| ncbi_105078609 | AHR | Up |  |
| ncbi_105079173 | JAK2 | Up |  |
| ncbi_105079416 | FOXP3 | Up |  |
| ncbi_105079801 | IL6 | Up |  |
| ncbi_105079942 | HSP90AA1 | Up |  |
| ncbi_105080117 | STAT5A | Up |  |
| ncbi_105080118 | STAT5B | Up |  |
| ncbi_105080511 | RORA | Up |  |
| ncbi_105081397 | IL22 | Up |  |
| ncbi_105082560 | JAK3 | Up |  |
| ncbi_105082567 | IL12RB1 | Up |  |
| ncbi_105082839 | Mapk3 | Down |  |
| ncbi_105082855 | LAT | Up |  |
| ncbi_105083690 | IL2RG | Up |  |
| ncbi_105083823 | HSP90AB1 | Up |  |
| MSTRG.11100 | TRAV16 | Up |  |
| MSTRG.11101 | TRAV18 | Up |  |
| MSTRG.11102 | TRAV18 | Up |  |
| MSTRG.11103 | TRAV8-4 | Up |  |
| MSTRG.2847 | TRBV16 | Up |  |
| MSTRG.2850 | TRBV14 | Up |  |
| MSTRG.2852 | TRBV10-3 | Up |  |
| MSTRG.2853 | TRBV7-9 | Up |  |
| MSTRG.2854 | TRBV5-5 | Up |  |
| MSTRG.2855 | V-TCR | Up |  |
| MSTRG.2857 | TRBV12-4 | Up |  |
| MSTRG.2858 | TRBV3-1 | Up |  |
| MSTRG.4011 | TRAV9-2 | Up |  |
| MSTRG.4012 | TRAV14DV4 | Up |  |
| MSTRG.4014 | TRAV9-2 | Up |  |
| MSTRG.4016 | TRAV8-3 | Up |  |
| MSTRG.4017 | TRAV9-1 | Up |  |
| MSTRG.4019 | TRAV2 | Up |  |
| MSTRG.6776 | TRDV1 | Up |  |
| MSTRG.6780 | TRAV23DV6 | Up |  |
| MSTRG.6781 | TRDV1 | Up |  |
| MSTRG.6787 | TRA | Up |  |
| MSTRG.84 | TRB | Up |  |
| MSTRG.85 | TRB | Up |  |
| MSTRG.86 | TRB | Up |  |
| MSTRG.87 | TRBV29-1 | Up |  |
| MSTRG.89 | TRBV27 | Up |  |
| ncbi_105062116 | STAT6 | Up |  |
| ncbi_105063497 | RUNX3 | Up |  |
| ncbi_105063980 | STAT4 | Up |  |
| ncbi_105063982 | STAT1 | Up |  |
| ncbi_105064175 | MAPK13 | Down |  |
| ncbi_105064235 | HLA-DOA | Up |  |
| ncbi_105064238 | HLA-DMA | Up |  |
| ncbi_105064239 | HLA-DMB | Up |  |
| ncbi_105064245 | HLA-DOB | Up |  |
| ncbi_105064246 | HLA-DRB1 | Up |  |
| ncbi_105064247 | HLA-DRB1 | Up |  |
| ncbi_105064248 | HLA-DQA2 | Up |  |
| ncbi_105064249 | HLA-DQB1 | Up |  |
| ncbi_105064250 | HLA-DRA | Up |  |
| ncbi_105064276 | HLA-DRB1 | Up |  |
| ncbi_105064618 | JAK1 | Up |  |
| ncbi_105064951 | JAG1 | Up |  |
| ncbi_105066646 | IL12A | Up |  |
| ncbi_105066766 | LCK | Up |  |
| ncbi_105067817 | CD3G | Up |  |
| ncbi_105067818 | CD3D | Up |  |
| ncbi_105067821 | CD3E | Up |  |
| ncbi_105067906 | PPP3CA | Up |  |
| ncbi_105067913 | NFKB1 | Up |  |
| ncbi_105068057 | CD247 | Up |  |
| ncbi_105068381 | TYK2 | Up |  |
| ncbi_105069046 | NFATC1 | Up |  |
| ncbi_105069349 | MAPK1 | Up |  |
| ncbi_105069770 | NOTCH1 | Up |  |
| ncbi_105070806 | MAPK12 | Up |  |
| ncbi_105070807 | MAPK11 | Up |  |
| ncbi_105071091 | CD4 | Up |  |
| ncbi_105071245 | IKBKB | Up |  |
| ncbi_105071674 | JUN | Down |  |
| ncbi_105071843 | NFATC2 | Up |  |
| ncbi_105071908 | RBPJL | Down |  |
| ncbi_105071939 | PLCG1 | Up |  |
| ncbi_105072189 | ZAP70 | Up |  |
| ncbi_105072900 | TRBV25-1 | Up |  |
| ncbi_105073156 | IL2RB | Up |  |
| ncbi_105073341 | TBX21 | Up |  |
| ncbi_105074228 | GATA3 | Up |  |
| ncbi_105074303 | PRKCQ | Up |  |
| ncbi_105074562 | NOTCH3 | Up |  |
| ncbi_105074697 | IL2RA | Up |  |
| ncbi_105074856 | NFATC3 | Up |  |
| ncbi_105075281 | RBPJ | Up |  |
| ncbi_105075289 | FOS | Down |  |
| ncbi_105077267 | IFNGR1 | Up |  |
| ncbi_105077723 | MAPK10 | Up |  |
| ncbi_105078829 | V-MAF | Up |  |
| ncbi_105079173 | JAK2 | Up |  |
| ncbi_105079218 | NOTCH2 | Up |  |
| ncbi_105080031 | JAG2 | Up |  |
| ncbi_105080117 | STAT5A | Up |  |
| ncbi_105080118 | STAT5B | Up |  |
| ncbi_105082560 | JAK3 | Up |  |
| ncbi_105082567 | IL12RB1 | Up |  |
| ncbi_105082839 | Mapk3 | Down |  |
| ncbi_105082855 | LAT | Up |  |
| ncbi_105083483 | MAML2 | Up |  |
| ncbi_105083690 | IL2RG | Up |  |
| MSTRG.11100 | TRAV16 | Up |  |
| MSTRG.11101 | TRAV18 | Up |  |
| MSTRG.11102 | TRAV18 | Up |  |
| MSTRG.11103 | TRAV8-4 | Up |  |
| MSTRG.16869 | MAML2 | Down |  |
| MSTRG.2847 | TRBV16 | Up |  |
| MSTRG.2850 | TRBV14 | Up |  |
| MSTRG.2852 | TRBV10-3 | Up |  |
| MSTRG.2853 | TRBV7-9 | Up |  |
| MSTRG.2854 | TRBV5-5 | Up |  |
| MSTRG.2855 | V-TCR | Up |  |
| MSTRG.2857 | TRBV12-4 | Up |  |
| MSTRG.2858 | TRBV3-1 | Up |  |
| MSTRG.4011 | TRAV9-2 | Up |  |
| MSTRG.4012 | TRAV14DV4 | Up |  |
| MSTRG.4014 | TRAV9-2 | Up |  |
| MSTRG.4016 | TRAV8-3 | Up |  |
| MSTRG.4017 | TRAV9-1 | Up |  |
| MSTRG.4019 | TRAV2 | Up |  |
| MSTRG.6776 | TRDV1 | Up |  |
| MSTRG.6780 | TRAV23DV6 | Up |  |
| MSTRG.6781 | TRDV1 | Up |  |
| MSTRG.6787 | TRA | Up |  |
| MSTRG.84 | TRB | Up |  |
| MSTRG.85 | TRB | Up |  |
| MSTRG.86 | TRB | Up |  |
| MSTRG.87 | TRBV29-1 | Up |  |
| MSTRG.89 | TRBV27 | Up |  |
| 1. **T cell receptor signaling pathway** | | |  |
| ncbi_105062297 | KRAS | Up |  |
| ncbi_105062855 | AKT2 | Up |  |
| ncbi_105063518 | PDCD1 | Up |  |
| ncbi_105064109 | MAP3K7 | Up |  |
| ncbi_105064175 | MAPK13 | Down |  |
| ncbi_105064416 | PIK3R1 | Up |  |
| ncbi_105065653 | GRB2 | Up |  |
| ncbi_105066766 | LCK | Up |  |
| ncbi_105067053 | CD40LG | Up |  |
| ncbi_105067817 | CD3G | Up |  |
| ncbi_105067818 | CD3D | Up |  |
| ncbi_105067821 | CD3E | Up |  |
| ncbi_105067906 | PPP3CA | Up |  |
| ncbi_105067913 | NFKB1 | Up |  |
| ncbi_105068057 | CD247 | Up |  |
| ncbi_105068613 | VAV2 | Up |  |
| ncbi_105069046 | NFATC1 | Up |  |
| ncbi_105069349 | MAPK1 | Up |  |
| ncbi_105069943 | HRAS | Down |  |
| ncbi_105070018 | PTPRC | Up |  |
| ncbi_105070806 | MAPK12 | Up |  |
| ncbi_105070807 | MAPK11 | Up |  |
| ncbi_105070875 | CDC42 | Up |  |
| ncbi_105071091 | CD4 | Up |  |
| ncbi_105071204 | PTPN6 | Up |  |
| ncbi_105071245 | IKBKB | Up |  |
| ncbi_105071674 | JUN | Down |  |
| ncbi_105071706 | MAP3K8 | Up |  |
| ncbi_105071843 | NFATC2 | Up |  |
| ncbi_105071939 | PLCG1 | Up |  |
| ncbi_105072189 | ZAP70 | Up |  |
| ncbi_105072260 | CD8A | Up |  |
| ncbi_105072377 | DLG1 | Up |  |
| ncbi_105072383 | Pak2 | Up |  |
| ncbi_105072525 | MAP3K14 | Up |  |
| ncbi_105072900 | TRBV25-1 | Up |  |
| ncbi_105073098 | Grap2 | Up |  |
| ncbi_105073996 | MALT1 | Up |  |
| ncbi_105074074 | TNF | Up |  |
| ncbi_105074303 | PRKCQ | Up |  |
| ncbi_105074856 | NFATC3 | Up |  |
| ncbi_105074908 | TEC | Up |  |
| ncbi_105075198 | PTPN6 | Up |  |
| ncbi_105075289 | FOS | Down |  |
| ncbi_105075425 | IL10 | Up |  |
| ncbi_105075749 | PAK3 | Up |  |
| ncbi_105076883 | RASGRP1 | Up |  |
| ncbi_105077134 | FYN | Up |  |
| ncbi_105077723 | MAPK10 | Up |  |
| ncbi_105078075 | CARD11 | Up |  |
| ncbi_105078399 | VAV1 | Up |  |
| ncbi_105078489 | SOS2 | Up |  |
| ncbi_105079555 | NRAS | Up |  |
| ncbi_105080142 | CBLB | Up |  |
| ncbi_105080927 | ITK | Up |  |
| ncbi_105082277 | ICOS | Up |  |
| ncbi_105082278 | CTLA4 | Up |  |
| ncbi_105082279 | CD28 | Up |  |
| ncbi_105082679 | PIK3R2 | Up |  |
| ncbi_105082694 | LCP2 | Up |  |
| ncbi_105082839 | Mapk3 | Down |  |
| ncbi_105082855 | LAT | Up |  |
| ncbi_105083596 | AKT3 | Up |  |
| ncbi_105083612 | PAK1 | Down |  |
| MSTRG.11100 | TRAV16 | Up |  |
| MSTRG.11101 | TRAV18 | Up |  |
| MSTRG.11102 | TRAV18 | Up |  |
| MSTRG.11103 | TRAV8-4 | Up |  |
| MSTRG.2847 | TRBV16 | Up |  |
| MSTRG.2850 | TRBV14 | Up |  |
| MSTRG.2852 | TRBV10-3 | Up |  |
| MSTRG.2853 | TRBV7-9 | Up |  |
| MSTRG.2854 | TRBV5-5 | Up |  |
| MSTRG.2855 | V-TCR | Up |  |
| MSTRG.2857 | TRBV12-4 | Up |  |
| MSTRG.2858 | TRBV3-1 | Up |  |
| MSTRG.4011 | TRAV9-2 | Up |  |
| MSTRG.4012 | TRAV14DV4 | Up |  |
| MSTRG.4014 | TRAV9-2 | Up |  |
| MSTRG.4016 | TRAV8-3 | Up |  |
| MSTRG.4017 | TRAV9-1 | Up |  |
| MSTRG.4019 | TRAV2 | Up |  |
| MSTRG.6776 | TRDV1 | Up |  |
| MSTRG.6780 | TRAV23DV6 | Up |  |
| MSTRG.6781 | TRDV1 | Up |  |
| MSTRG.6787 | TRA | Up |  |
| MSTRG.84 | TRB | Up |  |
| MSTRG.85 | TRB | Up |  |
| MSTRG.86 | TRB | Up |  |
| MSTRG.87 | TRBV29-1 | Up |  |
| MSTRG.89 | TRBV27 | Up |  |
| 1. **antigen processing and presentation** | | |  |
| ncbi_105061640 | HLA-A | Up |  |
| ncbi_105062008 | CANX | Up |  |
| ncbi_105062207 | KLRD1 | Up |  |
| ncbi_105062210 | KLRC1 | Up |  |
| ncbi_105064235 | HLA-DOA | Up |  |
| ncbi_105064238 | HLA-DMA | Up |  |
| ncbi_105064239 | HLA-DMB | Up |  |
| ncbi_105064241 | TAP1 | Up |  |
| ncbi_105064243 | TAP2 | Up |  |
| ncbi_105064245 | HLA-DOB | Up |  |
| ncbi_105064246 | HLA-DRB1 | Up |  |
| ncbi_105064247 | HLA-DRB1 | Up |  |
| ncbi_105064248 | HLA-DQA2 | Up |  |
| ncbi_105064249 | HLA-DQB1 | Up |  |
| ncbi_105064250 | HLA-DRA | Up |  |
| ncbi_105064276 | HLA-DRB1 | Up |  |
| ncbi_105065248 | Mr1 | Up |  |
| ncbi_105066308 | RFXAP | Up |  |
| ncbi_105068023 | HSPA6 | Up |  |
| ncbi_105071091 | CD4 | Up |  |
| ncbi_105071715 | RFX5 | Up |  |
| ncbi_105071741 | CTSS | Up |  |
| ncbi_105072260 | CD8A | Up |  |
| ncbi_105072753 | NFYC | Up |  |
| ncbi_105072900 | TRBV25-1 | Up |  |
| ncbi_105072963 | CREB1 | Up |  |
| ncbi_105074074 | TNF | Up |  |
| ncbi_105074084 | HLA-B | Up |  |
| ncbi_105074299 | CD74 | Up |  |
| ncbi_105074668 | HSPA2 | Up |  |
| ncbi_105076977 | B2M | Up |  |
| ncbi_105078638 | HSPA8 | Up |  |
| ncbi_105078803 | CTSL | Up |  |
| ncbi_105079874 | LGMN | Up |  |
| ncbi_105079942 | HSP90AA1 | Up |  |
| ncbi_105081302 | NFYB | Up |  |
| ncbi_105082116 | HSPA4 | Up |  |
| ncbi_105082680 | IFI30 | Up |  |
| ncbi_105083758 | NFYA | Up |  |
| ncbi_105083823 | HSP90AB1 | Up |  |
| ncbi_105084049 | Patr-A | Up |  |
| MSTRG.10587 | Patr-A | Up |  |
| MSTRG.11100 | TRAV16 | Up |  |
| MSTRG.11101 | TRAV18 | Up |  |
| MSTRG.11102 | TRAV18 | Up |  |
| MSTRG.11103 | TRAV8-4 | Up |  |
| MSTRG.2847 | TRBV16 | Up |  |
| MSTRG.2850 | TRBV14 | Up |  |
| MSTRG.2852 | TRBV10-3 | Up |  |
| MSTRG.2853 | TRBV7-9 | Up |  |
| MSTRG.2854 | TRBV5-5 | Up |  |
| MSTRG.2855 | V-TCR | Up |  |
| MSTRG.2857 | TRBV12-4 | Up |  |
| MSTRG.2858 | TRBV3-1 | Up |  |
| MSTRG.4011 | TRAV9-2 | Up |  |
| MSTRG.4012 | TRAV14DV4 | Up |  |
| MSTRG.4014 | TRAV9-2 | Up |  |
| MSTRG.4016 | TRAV8-3 | Up |  |
| MSTRG.4017 | TRAV9-1 | Up |  |
| MSTRG.4019 | TRAV2 | Up |  |
| MSTRG.4391 | HLA-C | Up |  |
| MSTRG.6776 | TRDV1 | Up |  |
| MSTRG.6780 | TRAV23DV6 | Up |  |
| MSTRG.6781 | TRDV1 | Up |  |
| MSTRG.6787 | TRA | Up |  |
| MSTRG.84 | TRB | Up |  |
| MSTRG.85 | TRB | Up |  |
| MSTRG.86 | TRB | Up |  |
| MSTRG.87 | TRBV29-1 | Up |  |
| MSTRG.89 | TRBV27 | Up |  |
