# Supplementary material for: Integrated omics analysis reveals the immunologic characteristics of cystic Peyer’s patches in the cecum of Bactrian camels
Source: PeerJ. 2023 Jan 9;11:e14647. doi: 10.7717/peerj.14647 (PMC9835693; doi:10.7717/peerj.14647)
Supplement: Table S2 [file peerj-11-14647-s002.docx]

Table S2. Differential expressed genes in multiple immune-related functions by GO enrichment analysis.

| Gene ID | Gene symbol | Up- or down-regulated |
| --- | --- | --- |
| ncbi_105062714 | NECTIN2 | Down |
| ncbi_105064363 | F2RL1 | Down |
| ncbi_105064505 | NLRP6 | Down |
| ncbi_105067509 | MUC12 | Down |
| ncbi_105067787 | NLRX1 | Down |
| ncbi_105068160 | MUC1 | Down |
| ncbi_105068211 | S100A14 | Down |
| ncbi_105068596 | C8G | Down |
| ncbi_105070033 | INAVA | Down |
| ncbi_105070452 | MUC5AC | Down |
| ncbi_105071585 | LGALS9 | Down |
| ncbi_105072080 | PLD2 | Down |
| ncbi_105073493 | Src | Down |
| ncbi_105074078 | NFKBIL1 | Down |
| ncbi_105074600 | LGALS3 | Down |
| ncbi_105075034 | NR1H4 | Down |
| ncbi_105075333 | SFTPD | Down |
| ncbi_105076928 | TYRO3 | Down |
| ncbi_105078179 | GNA11 | Down |
| ncbi_105078232 | TICAM1 | Down |
| ncbi_105078652 | BCAR1 | Down |
| ncbi_105080216 | MAPKAPK3 | Down |
| ncbi_105082839 | Mapk3 | Down |
| ncbi_105083612 | PAK1 | Down |
| ncbi_105083847 | TNFRSF21 | Down |
| ncbi_105084054 | TRIM15 | Down |
| ncbi_105062497 | PVR | Down |
| ncbi_105062622 | TRPM4 | Down |
| ncbi_105068168 | Zbtb7b | Down |
| ncbi_105071048 | Prkcz | Down |
| ncbi_105071703 | RORC | Down |
| ncbi_105073695 | ZP2 | Down |
| ncbi_105077504 | TNFSF13 | Down |
| ncbi_105080455 | GCNT3 | Down |
| ncbi_105084152 | HFE | Down |
| ncbi_105062554 | AP2S1 | Down |
| ncbi_105066456 | RAB3B | Down |
| ncbi_105068388 | AP1M2 | Down |
| ncbi_105068392 | Dnm2 | Down |
| ncbi_105069840 | ACE | Down |
| ncbi_105070308 | CTSF | Down |
| ncbi_105072102 | VAMP8 | Down |
| ncbi_105078337 | DNM2 | Down |
| ncbi_105079614 | CDH17 | Down |
| ncbi_105063810 | FOSL2 | Down |
| ncbi_105066015 | LCN2 | Down |
| ncbi_105067778 | USP2 | Down |
| ncbi_105071674 | JUN | Down |
| ncbi_105076636 | ADM | Down |
| ncbi_105080565 | PLA2G1B | Down |
| ncbi_105080854 | Spns2 | Down |
| ncbi_105082425 | MUC2 | Down |
| ncbi_105068247 | ADAM15 | Down |
| ncbi_105075289 | FOS | Down |
| MSTRG.2461 | CEACAM1 | Down |
| ncbi_105081824 | RBP4 | Down |
| ncbi_105064859 | GFER | Down |
| ncbi_105066883 | LAMP1 | Down |
| ncbi_105075274 | GZMB | Down |
| ncbi_105061675 | ADORA2B | Down |
| ncbi_105066096 | PLA2G3 | Down |
| ncbi_105063556 | RAB17 | Down |
| ncbi_105070842 | IFNLR1 | Down |
| ncbi_105073713 | GP2 | Down |
| ncbi_105061930 | TOM1 | Down |
| ncbi_105062266 | MGST1 | Down |
| ncbi_105062488 | TTR | Down |
| ncbi_105064789 | HMOX2 | Down |
| ncbi_105065590 | CANT1 | Down |
| ncbi_105069359 | BCR | Down |
| ncbi_105069742 | AGPAT2 | Down |
| ncbi_105070263 | PRDX6 | Down |
| ncbi_105070346 | GSTP1 | Down |
| ncbi_105070740 | ALAD | Down |
| ncbi_105070824 | ARSA | Down |
| ncbi_105070893 | Cda | Down |
| ncbi_105073791 | GSDMD | Down |
| ncbi_105073794 | NAPRT | Down |
| ncbi_105073815 | DGAT1 | Down |
| ncbi_105074045 | NEU1 | Down |
| ncbi_105075922 | CD63 | Down |
| ncbi_105076503 | COMMD9 | Down |
| ncbi_105077234 | VNN1 | Down |
| ncbi_105077271 | HEBP2 | Down |
| ncbi_105078014 | PFKL | Down |
| ncbi_105078327 | Rab3d | Down |
| ncbi_105079095 | RAB9B | Down |
| ncbi_105079334 | Jup | Down |
| ncbi_105080841 | AOC1 | Down |
| ncbi_105082194 | -- | Down |
| ncbi_105082877 | MVP | Down |
| ncbi_105068815 | DHRS1 | Down |
| ncbi_105067470 | PPARG | Down |
| ncbi_105068165 | DCST1 | Down |
| ncbi_105070638 | PRDX5 | Down |
| ncbi_105062241 | ADORA1 | Down |
| ncbi_105064029 | IHH | Down |
| ncbi_105064285 | MMP28 | Down |
| ncbi_105069071 | CIB1 | Down |
| ncbi_105069336 | DDT | Down |
| ncbi_105078064 | TFF2 | Down |
| ncbi_105078991 | Tmem178a | Down |
| ncbi_105080260 | TCTA | Down |
| ncbi_105082481 | GAS2L1 | Down |
| MSTRG.11018 | Pol | Up |
| MSTRG.11100 | TRAV16 | Up |
| MSTRG.11103 | TRAV8-4 | Up |
| MSTRG.1254 | Pol | Up |
| MSTRG.14532 | IGHG1 | Up |
| MSTRG.14534 | IGHG2 | Up |
| MSTRG.16018 | Pol | Up |
| MSTRG.1870 | Pol | Up |
| MSTRG.2128 | RIPK2 | Up |
| MSTRG.2294 | Clec2d11 | Up |
| MSTRG.2850 | TRBV14 | Up |
| MSTRG.2853 | TRBV7-9 | Up |
| MSTRG.2857 | TRBV12-4 | Up |
| MSTRG.38 | IGLV1-40 | Up |
| MSTRG.4015 | TRAV13-1 | Up |
| MSTRG.4016 | TRAV8-3 | Up |
| MSTRG.43 | IGLV1-40 | Up |
| MSTRG.44 | IGLV8-61 | Up |
| MSTRG.47 | IGLV8-61 | Up |
| MSTRG.48 | IGLV1-40 | Up |
| MSTRG.56 | IGLV8-61 | Up |
| MSTRG.57 | IGLV1-40 | Up |
| MSTRG.6780 | TRAV23DV6 | Up |
| MSTRG.6787 | TRA | Up |
| MSTRG.7219 | -- | Up |
| MSTRG.7644 | CFH | Up |
| MSTRG.7741 | Pol | Up |
| MSTRG.84 | TRB | Up |
| MSTRG.85 | TRB | Up |
| MSTRG.86 | TRB | Up |
| ncbi_105061950 | EIF2B3 | Up |
| ncbi_105062165 | CLEC4E | Up |
| ncbi_105062169 | C3AR1 | Up |
| ncbi_105062185 | A2M | Up |
| ncbi_105062201 | CLEC7A | Up |
| ncbi_105062206 | Klre1 | Up |
| ncbi_105062208 | KLRK1 | Up |
| ncbi_105062466 | CD79A | Up |
| ncbi_105062523 | RNF125 | Up |
| ncbi_105062540 | PGLYRP1 | Up |
| ncbi_105062558 | SAE1 | Up |
| ncbi_105062561 | C5AR1 | Up |
| ncbi_105062717 | CD226 | Up |
| ncbi_105062951 | Pum2 | Up |
| ncbi_105062962 | APOB | Up |
| ncbi_105063019 | BRAF | Up |
| ncbi_105063150 | CAV1 | Up |
| ncbi_105063343 | KLHL6 | Up |
| ncbi_105063426 | PTAFR | Up |
| ncbi_105063432 | THEMIS2 | Up |
| ncbi_105063438 | FGR | Up |
| ncbi_105063442 | WASF2 | Up |
| ncbi_105063578 | INPP5D | Up |
| ncbi_105063642 | PRKDC | Up |
| ncbi_105063692 | RAP1A | Up |
| ncbi_105063875 | CLU | Up |
| ncbi_105063974 | Slc39a10 | Up |
| ncbi_105063988 | XRCC5 | Up |
| ncbi_105064002 | ARPC2 | Up |
| ncbi_105064109 | MAP3K7 | Up |
| ncbi_105064250 | HLA-DRA | Up |
| ncbi_105064416 | PIK3R1 | Up |
| ncbi_105064418 | Cd180 | Up |
| ncbi_105064730 | RIPK2 | Up |
| ncbi_105064750 | CXorf21 | Up |
| ncbi_105064798 | CREBBP | Up |
| ncbi_105064893 | CGAS | Up |
| ncbi_105065009 | Uba2 | Up |
| ncbi_105065061 | Nfkbid | Up |
| ncbi_105065089 | LSM14A | Up |
| ncbi_105065195 | DDX60 | Up |
| ncbi_105065267 | C5 | Up |
| ncbi_105065282 | C5 | Up |
| ncbi_105065609 | Sec14l1 | Up |
| ncbi_105065653 | GRB2 | Up |
| ncbi_105065688 | CD300A | Up |
| ncbi_105066195 | LY96 | Up |
| ncbi_105066234 | HMGB1 | Up |
| ncbi_105066256 | USP12 | Up |
| ncbi_105066286 | PSPC1 | Up |
| ncbi_105066381 | PAG1 | Up |
| ncbi_105066402 | ARRB2 | Up |
| ncbi_105066513 | RPS27A | Up |
| ncbi_105066550 | PELI1 | Up |
| ncbi_105066766 | LCK | Up |
| ncbi_105066790 | Pum1 | Up |
| ncbi_105066936 | SERPING1 | Up |
| ncbi_105067152 | TNIP3 | Up |
| ncbi_105067238 | CFH | Up |
| ncbi_105067355 | Pax5 | Up |
| ncbi_105067455 | IRAK2 | Up |
| ncbi_105067517 | SH2B2 | Up |
| ncbi_105067556 | Nr4a3 | Up |
| ncbi_105067776 | THY1 | Up |
| ncbi_105067817 | CD3G | Up |
| ncbi_105067818 | CD3D | Up |
| ncbi_105067821 | CD3E | Up |
| ncbi_105067913 | NFKB1 | Up |
| ncbi_105067955 | Cfi | Up |
| ncbi_105068021 | FCGR2 | Up |
| ncbi_105068024 | FCGR2 | Up |
| ncbi_105068057 | CD247 | Up |
| ncbi_105068220 | S100A8 | Up |
| ncbi_105068222 | S100A9 | Up |
| ncbi_105068379 | ICAM3 | Up |
| ncbi_105068458 | LIME1 | Up |
| ncbi_105068613 | VAV2 | Up |
| ncbi_105068672 | OTUD4 | Up |
| ncbi_105068687 | MARCO | Up |
| ncbi_105068701 | ELF2 | Up |
| ncbi_105068899 | RC3H1 | Up |
| ncbi_105069174 | Sin3a | Up |
| ncbi_105069302 | ELMO1 | Up |
| ncbi_105069349 | MAPK1 | Up |
| ncbi_105069552 | Fcer1g | Up |
| ncbi_105069710 | RAPGEF1 | Up |
| ncbi_105069735 | CARD9 | Up |
| ncbi_105069778 | CD79B | Up |
| ncbi_105069905 | IRF7 | Up |
| ncbi_105069927 | RC3H2 | Up |
| ncbi_105070018 | PTPRC | Up |
| ncbi_105070021 | DENND1B | Up |
| ncbi_105070053 | LPXN | Up |
| ncbi_105070100 | TKFC | Up |
| ncbi_105070210 | RUNX1 | Up |
| ncbi_105070311 | RBM14 | Up |
| ncbi_105070384 | CD81 | Up |
| ncbi_105070509 | FCRL3 | Up |
| ncbi_105070511 | FCRL5 | Up |
| ncbi_105070693 | FYB1 | Up |
| ncbi_105070721 | TLR4 | Up |
| ncbi_105070724 | C9 | Up |
| ncbi_105070775 | OTULIN | Up |
| ncbi_105070819 | C7 | Up |
| ncbi_105070840 | C6 | Up |
| ncbi_105070868 | C1QB | Up |
| ncbi_105070870 | C1qc | Up |
| ncbi_105070871 | C1QA | Up |
| ncbi_105070875 | CDC42 | Up |
| ncbi_105071055 | CDK11B | Up |
| ncbi_105071091 | CD4 | Up |
| ncbi_105071245 | IKBKB | Up |
| ncbi_105071401 | YES1 | Up |
| ncbi_105071448 | PTPN2 | Up |
| ncbi_105071463 | COLEC12 | Up |
| ncbi_105071518 | MYO1C | Up |
| ncbi_105071519 | CRK | Up |
| ncbi_105071576 | SARM1 | Up |
| ncbi_105071659 | PIK3C3 | Up |
| ncbi_105071741 | CTSS | Up |
| ncbi_105071843 | NFATC2 | Up |
| ncbi_105071879 | Elmo2 | Up |
| ncbi_105071939 | PLCG1 | Up |
| ncbi_105072189 | ZAP70 | Up |
| ncbi_105072295 | TNIP2 | Up |
| ncbi_105072340 | MASP1 | Up |
| ncbi_105072383 | Pak2 | Up |
| ncbi_105072832 | SFPQ | Up |
| ncbi_105073084 | EP300 | Up |
| ncbi_105073098 | Grap2 | Up |
| ncbi_105073232 | CCR7 | Up |
| ncbi_105073342 | SKAP1 | Up |
| ncbi_105073450 | Wipf1 | Up |
| ncbi_105073505 | SLA2 | Up |
| ncbi_105073541 | ITCH | Up |
| ncbi_105073576 | HCK | Up |
| ncbi_105073669 | Prkcb | Up |
| ncbi_105073903 | BCL2 | Up |
| ncbi_105073996 | MALT1 | Up |
| ncbi_105074038 | C4A | Up |
| ncbi_105074040 | CFB | Up |
| ncbi_105074074 | TNF | Up |
| ncbi_105074228 | GATA3 | Up |
| ncbi_105074267 | CYLD | Up |
| ncbi_105074268 | NOD2 | Up |
| ncbi_105074303 | PRKCQ | Up |
| ncbi_105074544 | PGLYRP2 | Up |
| ncbi_105074645 | PRKCH | Up |
| ncbi_105074785 | MEF2C | Up |
| ncbi_105074796 | CMTM3 | Up |
| ncbi_105074808 | CBFB | Up |
| ncbi_105074900 | USP46 | Up |
| ncbi_105074908 | TEC | Up |
| ncbi_105074909 | TXK | Up |
| ncbi_105074959 | TLR1 | Up |
| ncbi_105074961 | TLR10 | Up |
| ncbi_105075198 | PTPN6 | Up |
| ncbi_105075416 | CR2 | Up |
| ncbi_105075418 | C4BPA | Up |
| ncbi_105075427 | MAPKAPK2 | Up |
| ncbi_105075442 | Rab29 | Up |
| ncbi_105075451 | CR1 | Up |
| ncbi_105075498 | PRKACB | Up |
| ncbi_105075508 | C1S | Up |
| ncbi_105075561 | PDE4B | Up |
| ncbi_105075564 | ACTB | Up |
| ncbi_105075582 | C1R | Up |
| ncbi_105075749 | PAK3 | Up |
| ncbi_105075816 | ACTR2 | Up |
| ncbi_105076003 | USP9X | Up |
| ncbi_105076044 | RSAD2 | Up |
| ncbi_105076188 | CACNB3 | Up |
| ncbi_105076317 | NCKAP1L | Up |
| ncbi_105076321 | TESPA1 | Up |
| ncbi_105076433 | PTPRJ | Up |
| ncbi_105076558 | MUC15 | Up |
| ncbi_105077134 | FYN | Up |
| ncbi_105077207 | THEMIS | Up |
| ncbi_105077308 | Tab2 | Up |
| ncbi_105077436 | TNFAIP3 | Up |
| ncbi_105077475 | CLEC10A | Up |
| ncbi_105077614 | RFTN1 | Up |
| ncbi_105077753 | TLR7 | Up |
| ncbi_105077754 | TLR8 | Up |
| ncbi_105077776 | LRRC70 | Up |
| ncbi_105077788 | Pde4d | Up |
| ncbi_105077858 | MAP3K1 | Up |
| ncbi_105077943 | PROS1 | Up |
| ncbi_105077986 | S100B | Up |
| ncbi_105078000 | ITGB2 | Up |
| ncbi_105078041 | Ubash3a | Up |
| ncbi_105078075 | CARD11 | Up |
| ncbi_105078127 | STK11 | Up |
| ncbi_105078237 | PTPRS | Up |
| ncbi_105078264 | C3 | Up |
| ncbi_105078399 | VAV1 | Up |
| ncbi_105078440 | ARF6 | Up |
| ncbi_105078621 | CD46 | Up |
| ncbi_105078730 | LTF | Up |
| ncbi_105078736 | DDX58 | Up |
| ncbi_105078779 | UBQLN1 | Up |
| ncbi_105078885 | STAP1 | Up |
| ncbi_105078902 | LAX1 | Up |
| ncbi_105078942 | PLCG2 | Up |
| ncbi_105078949 | NLRC4 | Up |
| ncbi_105079021 | PRKCE | Up |
| ncbi_105079220 | ABI1 | Up |
| ncbi_105079377 | CFP | Up |
| ncbi_105079392 | WAS | Up |
| ncbi_105079416 | FOXP3 | Up |
| ncbi_105079555 | NRAS | Up |
| ncbi_105079673 | PTPN22 | Up |
| ncbi_105079727 | TLR2 | Up |
| ncbi_105079757 | Dusp22 | Up |
| ncbi_105079861 | RPS6KA5 | Up |
| ncbi_105079948 | Traf3 | Up |
| ncbi_105080142 | CBLB | Up |
| ncbi_105080390 | ANKRD17 | Up |
| ncbi_105080508 | SYK | Up |
| ncbi_105080530 | MYO1G | Up |
| ncbi_105080594 | NAA25 | Up |
| ncbi_105080614 | ARPC3 | Up |
| ncbi_105080678 | SCARB1 | Up |
| ncbi_105080742 | BTK | Up |
| ncbi_105080796 | PLEKHA1 | Up |
| ncbi_105080925 | HAVCR2 | Up |
| ncbi_105080927 | ITK | Up |
| ncbi_105080929 | Cyfip2 | Up |
| ncbi_105081080 | TAB3 | Up |
| ncbi_105081131 | CXorf21 | Up |
| ncbi_105081297 | HSP90B1 | Up |
| ncbi_105081349 | IRAK3 | Up |
| ncbi_105081359 | Tbk1 | Up |
| ncbi_105081383 | IRAK4 | Up |
| ncbi_105081543 | TRAT1 | Up |
| ncbi_105081555 | GCSAM | Up |
| ncbi_105081617 | CD86 | Up |
| ncbi_105081892 | PIK3AP1 | Up |
| ncbi_105081934 | NOD1 | Up |
| ncbi_105081945 | TANK | Up |
| ncbi_105081951 | IFIH1 | Up |
| ncbi_105081995 | UBR3 | Up |
| ncbi_105082022 | PJA2 | Up |
| ncbi_105082043 | TICAM2 | Up |
| ncbi_105082153 | TRIL | Up |
| ncbi_105082179 | MATR3 | Up |
| ncbi_105082208 | CD14 | Up |
| ncbi_105082278 | CTLA4 | Up |
| ncbi_105082279 | CD28 | Up |
| ncbi_105082327 | HSPD1 | Up |
| ncbi_105082679 | PIK3R2 | Up |
| ncbi_105082694 | LCP2 | Up |
| ncbi_105082735 | LIMK1 | Up |
| ncbi_105082737 | LAT2 | Up |
| ncbi_105082750 | RABGEF1 | Up |
| ncbi_105082773 | ITGAM | Up |
| ncbi_105082855 | LAT | Up |
| ncbi_105083195 | DDX60 | Up |
| ncbi_105083239 | Cd38 | Up |
| ncbi_105083414 | ACTR3 | Up |
| ncbi_105083440 | Rgcc | Up |
| ncbi_105083447 | ELF1 | Up |
| ncbi_105083471 | BIRC2 | Up |
| ncbi_105083472 | BIRC3 | Up |
| ncbi_105083823 | HSP90AB1 | Up |
| ncbi_105083830 | CDC5L | Up |
| ncbi_105083952 | Susd4 | Up |
| ncbi_105083953 | TLR5 | Up |
| ncbi_105061720 | MLH1 | Up |
| ncbi_105062116 | STAT6 | Up |
| ncbi_105062175 | AICDA | Up |
| ncbi_105062421 | PIK3CG | Up |
| ncbi_105062459 | Pou2f2 | Up |
| ncbi_105062499 | BCL3 | Up |
| ncbi_105062879 | TGFB1 | Up |
| ncbi_105063338 | LAMP3 | Up |
| ncbi_105063850 | PTK2B | Up |
| ncbi_105064238 | HLA-DMA | Up |
| ncbi_105064241 | TAP1 | Up |
| ncbi_105064320 | XRCC4 | Up |
| ncbi_105064571 | ERAP2 | Up |
| ncbi_105064572 | ERAP1 | Up |
| ncbi_105064739 | NBN | Up |
| ncbi_105064951 | JAG1 | Up |
| ncbi_105065211 | RIF1 | Up |
| ncbi_105065218 | NLRP3 | Up |
| ncbi_105065395 | JAM3 | Up |
| ncbi_105065533 | CD7 | Up |
| ncbi_105065629 | FOXJ1 | Up |
| ncbi_105065802 | UNC13D | Up |
| ncbi_105065925 | SIGLEC11 | Up |
| ncbi_105066389 | SIRT1 | Up |
| ncbi_105066404 | ALOX15 | Up |
| ncbi_105066646 | IL12A | Up |
| ncbi_105066795 | RNF19B | Up |
| ncbi_105066901 | TNFSF13B | Up |
| ncbi_105066903 | LIG4 | Up |
| ncbi_105067053 | CD40LG | Up |
| ncbi_105067541 | SIT1 | Up |
| ncbi_105067944 | LEF1 | Up |
| ncbi_105068011 | SHLD2 | Up |
| ncbi_105068033 | SH2D1B | Up |
| ncbi_105068034 | SLAMF6 | Up |
| ncbi_105068132 | SEMA4A | Up |
| ncbi_105068307 | IL27RA | Up |
| ncbi_105068375 | ICAM1 | Up |
| ncbi_105069475 | CD1B | Up |
| ncbi_105069481 | CD1A | Up |
| ncbi_105069496 | FCER1A | Up |
| ncbi_105069524 | SLAMF6 | Up |
| ncbi_105069525 | CD84 | Up |
| ncbi_105069526 | SLAMF1 | Up |
| ncbi_105069528 | SLAMF7 | Up |
| ncbi_105069531 | LY9 | Up |
| ncbi_105069532 | CD244 | Up |
| ncbi_105070182 | CD6 | Up |
| ncbi_105070328 | Clcf1 | Up |
| ncbi_105070459 | IL13RA2 | Up |
| ncbi_105070510 | FCRL4 | Up |
| ncbi_105071106 | CD27 | Up |
| ncbi_105071369 | PAXIP1 | Up |
| ncbi_105071462 | THOC1 | Up |
| ncbi_105071599 | ATAD5 | Up |
| ncbi_105071882 | CD40 | Up |
| ncbi_105072137 | IL1B | Up |
| ncbi_105072177 | ARID5A | Up |
| ncbi_105072217 | IL1R1 | Up |
| ncbi_105072221 | IL18R1 | Up |
| ncbi_105072222 | IL18RAP | Up |
| ncbi_105072260 | CD8A | Up |
| ncbi_105072345 | BCL6 | Up |
| ncbi_105072377 | DLG1 | Up |
| ncbi_105072388 | RNF168 | Up |
| ncbi_105072396 | TFRC | Up |
| ncbi_105072477 | DCLRE1C | Up |
| ncbi_105072538 | NSD2 | Up |
| ncbi_105072629 | Eomes | Up |
| ncbi_105072660 | EPHB6 | Up |
| ncbi_105072662 | GPR183 | Up |
| ncbi_105073097 | SAMSN1 | Up |
| ncbi_105073193 | TNFRSF13C | Up |
| ncbi_105073341 | TBX21 | Up |
| ncbi_105073406 | CD274 | Up |
| ncbi_105073415 | IL33 | Up |
| ncbi_105073987 | TNFRSF11A | Up |
| ncbi_105074023 | AGER | Up |
| ncbi_105074075 | LTA | Up |
| ncbi_105074241 | TNFRSF17 | Up |
| ncbi_105074271 | ADCY7 | Up |
| ncbi_105074299 | CD74 | Up |
| ncbi_105074340 | LAIR1 | Up |
| ncbi_105074667 | ZBTB1 | Up |
| ncbi_105075305 | BATF | Up |
| ncbi_105075425 | IL10 | Up |
| ncbi_105075454 | FCAMR | Up |
| ncbi_105075556 | IL23R | Up |
| ncbi_105075667 | UNG | Up |
| ncbi_105075938 | SH2D1A | Up |
| ncbi_105076036 | ADAM17 | Up |
| ncbi_105076509 | Cd44 | Up |
| ncbi_105076638 | SWAP70 | Up |
| ncbi_105077043 | EIF2AK4 | Up |
| ncbi_105077060 | TP53BP1 | Up |
| ncbi_105077226 | STX7 | Up |
| ncbi_105077295 | STX11 | Up |
| ncbi_105077310 | PMS2 | Up |
| ncbi_105077790 | GAPT | Up |
| ncbi_105077802 | IL6ST | Up |
| ncbi_105077860 | IL31RA | Up |
| ncbi_105078059 | AIRE | Up |
| ncbi_105078061 | ICOSLG | Up |
| ncbi_105078074 | LOXL3 | Up |
| ncbi_105078212 | EBI3 | Up |
| ncbi_105078268 | ADGRE1 | Up |
| ncbi_105078407 | FCER2 | Up |
| ncbi_105078638 | HSPA8 | Up |
| ncbi_105078644 | CRTAM | Up |
| ncbi_105078727 | Ccr2 | Up |
| ncbi_105078772 | Ccl19 | Up |
| ncbi_105078803 | CTSL | Up |
| ncbi_105079027 | SOCS5 | Up |
| ncbi_105079035 | MSH2 | Up |
| ncbi_105079039 | MSH6 | Up |
| ncbi_105079173 | JAK2 | Up |
| ncbi_105079639 | SASH3 | Up |
| ncbi_105079801 | IL6 | Up |
| ncbi_105080059 | IL18 | Up |
| ncbi_105080085 | CCR6 | Up |
| ncbi_105080141 | ALCAM | Up |
| ncbi_105080402 | JCHAIN | Up |
| ncbi_105080423 | CXCL13 | Up |
| ncbi_105080477 | NEDD4 | Up |
| ncbi_105080511 | RORA | Up |
| ncbi_105080621 | P2RX7 | Up |
| ncbi_105080629 | ORAI1 | Up |
| ncbi_105081591 | CD80 | Up |
| ncbi_105081708 | NFKB2 | Up |
| ncbi_105081852 | FAS | Up |
| ncbi_105081933 | TNFRSF13B | Up |
| ncbi_105082002 | DPP4 | Up |
| ncbi_105082027 | CAMK4 | Up |
| ncbi_105082560 | JAK3 | Up |
| ncbi_105082567 | IL12RB1 | Up |
| ncbi_105082942 | CYRIB | Up |
| ncbi_105083520 | CTSC | Up |
| ncbi_105083560 | EXO1 | Up |
| ncbi_105083767 | TFEB | Up |
| ncbi_105083961 | Dusp10 | Up |
| ncbi_105084034 | HLX | Up |
| ncbi_105084090 | TRIM27 | Up |
| ncbi_105062366 | DMTF1 | Up |
| ncbi_105062804 | ZNF271 | Up |
| ncbi_105065506 | ATP7A | Up |
| ncbi_105081428 | FOXP1 | Up |
| MSTRG.10587 | Patr-A | Up |
| ncbi_105061640 | HLA-A | Up |
| ncbi_105061802 | KIF15 | Up |
| ncbi_105061896 | SH3GL2 | Up |
| ncbi_105061944 | KIF2C | Up |
| ncbi_105062008 | CANX | Up |
| ncbi_105062132 | KIF5A | Up |
| ncbi_105063698 | CAPZA1 | Up |
| ncbi_105063861 | RAB10 | Up |
| ncbi_105063862 | KIF3C | Up |
| ncbi_105064235 | HLA-DOA | Up |
| ncbi_105064239 | HLA-DMB | Up |
| ncbi_105064242 | PSMB8 | Up |
| ncbi_105064245 | HLA-DOB | Up |
| ncbi_105064246 | HLA-DRB1 | Up |
| ncbi_105064247 | HLA-DRB1 | Up |
| ncbi_105064248 | HLA-DQA2 | Up |
| ncbi_105064249 | HLA-DQB1 | Up |
| ncbi_105064276 | HLA-DRB1 | Up |
| ncbi_105064353 | AP3B1 | Up |
| ncbi_105064569 | LNPEP | Up |
| ncbi_105065234 | Sec31a | Up |
| ncbi_105065851 | CLTC | Up |
| ncbi_105066246 | FLT3 | Up |
| ncbi_105067134 | SEC24D | Up |
| ncbi_105067922 | CENPE | Up |
| ncbi_105067950 | SEC24B | Up |
| ncbi_105069231 | KIF23 | Up |
| ncbi_105069387 | MARCHF1 | Up |
| ncbi_105070909 | CAPZB | Up |
| ncbi_105071092 | LAG3 | Up |
| ncbi_105071563 | RAB34 | Up |
| ncbi_105072910 | ITGAV | Up |
| ncbi_105073672 | DCTN5 | Up |
| ncbi_105074084 | HLA-B | Up |
| ncbi_105074307 | Dctn4 | Up |
| ncbi_105075595 | Rab8b | Up |
| ncbi_105076220 | RACGAP1 | Up |
| ncbi_105076550 | KIF18A | Up |
| ncbi_105076940 | Snap23 | Up |
| ncbi_105077089 | ATG5 | Up |
| ncbi_105077779 | KIF2A | Up |
| ncbi_105077789 | RAB3C | Up |
| ncbi_105078362 | AP3D1 | Up |
| ncbi_105078773 | CCL21 | Up |
| ncbi_105079208 | Sec22b | Up |
| ncbi_105079300 | AP1S2 | Up |
| ncbi_105079648 | RAB33A | Up |
| ncbi_105079874 | LGMN | Up |
| ncbi_105081829 | KIF11 | Up |
| ncbi_105082108 | KIF3A | Up |
| ncbi_105082130 | SAR1B | Up |
| ncbi_105082132 | SEC24A | Up |
| ncbi_105082486 | TMEM106B | Up |
| ncbi_105082680 | IFI30 | Up |
| ncbi_105082774 | PYCARD | Up |
| ncbi_105082817 | KIF22 | Up |
| ncbi_105083304 | RAB6A | Up |
| ncbi_105083761 | TREM2 | Up |
| ncbi_105084049 | Patr-A | Up |
| ncbi_105062992 | DOCK11 | Up |
| ncbi_105064071 | DOCK10 | Up |
| ncbi_105065498 | Itm2a | Up |
| ncbi_105073142 | LGALS1 | Up |
| ncbi_105073150 | MFNG | Up |
| ncbi_105079218 | NOTCH2 | Up |
| ncbi_105061840 | TRIM6 | Up |
| ncbi_105065032 | FFAR3 | Up |
| ncbi_105066875 | GAS6 | Up |
| ncbi_105067114 | IL21 | Up |
| ncbi_105067841 | APOA1 | Up |
| ncbi_105069198 | SEMA7A | Up |
| ncbi_105069553 | APOA2 | Up |
| ncbi_105069653 | ANGPT1 | Up |
| ncbi_105071266 | TEK | Up |
| ncbi_105072636 | TGFBR2 | Up |
| ncbi_105073714 | GPRC5B | Up |
| ncbi_105075375 | TGFB3 | Up |
| ncbi_105078739 | Kars1 | Up |
| ncbi_105079594 | WNT5A | Up |
| ncbi_105081548 | CD96 | Up |
| ncbi_105082675 | CCDC194 | Up |
| ncbi_105083972 | Tgfb2 | Up |
| MSTRG.6068 | DEFB103A | Up |
| ncbi_105061639 | Ly86 | Up |
| ncbi_105061687 | CD83 | Up |
| ncbi_105062624 | CD37 | Up |
| ncbi_105063379 | BMI1 | Up |
| ncbi_105063410 | YTHDF2 | Up |
| ncbi_105063518 | PDCD1 | Up |
| ncbi_105064083 | CCL20 | Up |
| ncbi_105064710 | RNASE6 | Up |
| ncbi_105064890 | UBE2I | Up |
| ncbi_105066278 | IL7 | Up |
| ncbi_105066467 | Faf1 | Up |
| ncbi_105067599 | MMP7 | Up |
| ncbi_105068713 | NUP88 | Up |
| ncbi_105068865 | RARRES2 | Up |
| ncbi_105069602 | RPL30 | Up |
| ncbi_105069770 | NOTCH1 | Up |
| ncbi_105070085 | MS4A1 | Up |
| ncbi_105070900 | PLA2G2A | Up |
| ncbi_105071199 | MXRA8 | Up |
| ncbi_105071773 | H2B-I | Up |
| ncbi_105072335 | ST6GAL1 | Up |
| ncbi_105073482 | BPI | Up |
| ncbi_105075281 | RBPJ | Up |
| ncbi_105076881 | LYZ | Up |
| ncbi_105077332 | VIP | Up |
| ncbi_105078293 | CCL25 | Up |
| ncbi_105078516 | CXCL12 | Up |
| ncbi_105078691 | CCL22 | Up |
| ncbi_105079146 | PPP2R3C | Up |
| ncbi_105079789 | LY86 | Up |
| ncbi_105079814 | NPY | Up |
| ncbi_105080041 | Pou2af1 | Up |
| ncbi_105080362 | CXCL11 | Up |
| ncbi_105080363 | CXCL10 | Up |
| ncbi_105080364 | CXCL9 | Up |
| ncbi_105081282 | CCL8 | Up |
| ncbi_105081283 | CCL2 | Up |
| ncbi_105082201 | ANKHD1 | Up |
| ncbi_105084023 | PARP1 | Up |
| ncbi_105084136 | H2BC11 | Up |
| ncbi_105067906 | PPP3CA | Up |
| ncbi_105068022 | FCGR3 | Up |
| ncbi_105069046 | NFATC1 | Up |
| ncbi_105070081 | Ms4a2 | Up |
| ncbi_105074326 | Oscar | Up |
| ncbi_105074856 | NFATC3 | Up |
| ncbi_105077723 | MAPK10 | Up |
| ncbi_105082020 | FER | Up |
| ncbi_105064336 | MSH3 | Up |
| ncbi_105065155 | IKZF1 | Up |
| ncbi_105069346 | VPREB3 | Up |
| ncbi_105071078 | Tnfrsf4 | Up |
| ncbi_105072204 | REV1 | Up |
| ncbi_105075089 | GALNT2 | Up |
| ncbi_105078145 | Tcf3 | Up |
| ncbi_105079111 | NUGGC | Up |
| ncbi_105080694 | POLM | Up |
| ncbi_105081608 | POLQ | Up |
| ncbi_105082182 | MZB1 | Up |
| ncbi_105069118 | IL16 | Up |
| ncbi_105069442 | PDZD2 | Up |
| ncbi_105062193 | KLRF2 | Up |
| ncbi_105064901 | DNASE1 | Up |
| ncbi_105065001 | CEBPG | Up |
| ncbi_105067849 | Cadm1 | Up |
| ncbi_105070386 | IGF2 | Up |
| ncbi_105074100 | TUBB5 | Up |
| ncbi_105074347 | NCR1 | Up |
| ncbi_105076883 | RASGRP1 | Up |
| ncbi_105077550 | PIK3R6 | Up |
| ncbi_105079566 | DNASE1L3 | Up |
| ncbi_105079768 | SERPINB9 | Up |
| ncbi_105080118 | STAT5B | Up |
| ncbi_105082742 | NCF1 | Up |
| ncbi_105082840 | CORO1A | Up |
| ncbi_105061888 | RPS6 | Up |
| ncbi_105066132 | LCP1 | Up |
| ncbi_105076865 | APBB1IP | Up |
| ncbi_105079217 | APBB1IP | Up |
| ncbi_105082804 | ITGAL | Up |
| ncbi_105062207 | KLRD1 | Up |
| ncbi_105063956 | MILR1 | Up |
| ncbi_105065956 | STXBP1 | Up |
| ncbi_105067865 | CBL | Up |
| ncbi_105071482 | CRHR1 | Up |
| ncbi_105072411 | SNX4 | Up |
| ncbi_105073153 | RAC2 | Up |
| ncbi_105073933 | GATA2 | Up |
| ncbi_105076528 | C12orf4 | Up |
| ncbi_105079453 | FOXF1 | Up |
| ncbi_105083251 | DHX30 | Up |
| ncbi_105083553 | GAB2 | Up |
| ncbi_105077460 | MAP3K4 | Up |
| ncbi_105082996 | PLCB4 | Up |
| ncbi_105061746 | CX3CR1 | Up |
| ncbi_105061978 | FRMPD3 | Up |
| ncbi_105062041 | RAB24 | Up |
| ncbi_105062047 | HK3 | Up |
| ncbi_105062055 | FAF2 | Up |
| ncbi_105062085 | Dok3 | Up |
| ncbi_105062195 | CLEC12A | Up |
| ncbi_105062282 | CHI3L1 | Up |
| ncbi_105062293 | LRMP | Up |
| ncbi_105062395 | FGL2 | Up |
| ncbi_105062405 | PSMC2 | Up |
| ncbi_105062462 | DSG1 | Up |
| ncbi_105062690 | CD33 | Up |
| ncbi_105062835 | GMFG | Up |
| ncbi_105063007 | CLEC5A | Up |
| ncbi_105063106 | CDK13 | Up |
| ncbi_105063107 | IMPDH1 | Up |
| ncbi_105063269 | DNAJC13 | Up |
| ncbi_105063335 | ATP11B | Up |
| ncbi_105063643 | PSMA5 | Up |
| ncbi_105063676 | CD53 | Up |
| ncbi_105063945 | PSMD12 | Up |
| ncbi_105063957 | PECAM1 | Up |
| ncbi_105064101 | STK11IP | Up |
| ncbi_105064292 | CCL3 | Up |
| ncbi_105064445 | ARSB | Up |
| ncbi_105064912 | MMP25 | Up |
| ncbi_105064955 | SNAP25 | Up |
| ncbi_105065063 | TYROBP | Up |
| ncbi_105065176 | PLAC8 | Up |
| ncbi_105065350 | AGL | Up |
| ncbi_105065405 | CCT2 | Up |
| ncbi_105065582 | GAA | Up |
| ncbi_105065870 | MPO | Up |
| ncbi_105066392 | FABP5 | Up |
| ncbi_105066622 | CPNE3 | Up |
| ncbi_105066707 | SUCNR1 | Up |
| ncbi_105066846 | ARMC8 | Up |
| ncbi_105066996 | CEP290 | Up |
| ncbi_105067019 | ADAM8 | Up |
| ncbi_105067283 | OSTF1 | Up |
| ncbi_105067314 | VCP | Up |
| ncbi_105067396 | ERP44 | Up |
| ncbi_105067839 | PAFAH1B2 | Up |
| ncbi_105068023 | HSPA6 | Up |
| ncbi_105068058 | CREG1 | Up |
| ncbi_105068078 | SELL | Up |
| ncbi_105068389 | SLC44A2 | Up |
| ncbi_105069087 | IQGAP1 | Up |
| ncbi_105069823 | PSMD1 | Up |
| ncbi_105070029 | PKP1 | Up |
| ncbi_105070269 | CCT8 | Up |
| ncbi_105070332 | TBC1D10C | Up |
| ncbi_105070479 | STXBP3 | Up |
| ncbi_105071023 | KCNAB2 | Up |
| ncbi_105071154 | TNFRSF1B | Up |
| ncbi_105071393 | ROCK1 | Up |
| ncbi_105071569 | ALDOC | Up |
| ncbi_105071799 | CTSZ | Up |
| ncbi_105071885 | MMP9 | Up |
| ncbi_105072053 | PTPRB | Up |
| ncbi_105072514 | CYBB | Up |
| ncbi_105072641 | CMTM6 | Up |
| ncbi_105072704 | DNAJC3 | Up |
| ncbi_105072769 | PPIE | Up |
| ncbi_105073201 | SYNGR1 | Up |
| ncbi_105073293 | Kpnb1 | Up |
| ncbi_105073503 | DSN1 | Up |
| ncbi_105074688 | ATP6V1D | Up |
| ncbi_105074965 | PGM2 | Up |
| ncbi_105074982 | ATP8A1 | Up |
| ncbi_105075016 | LTA4H | Up |
| ncbi_105075026 | APAF1 | Up |
| ncbi_105075359 | PLAU | Up |
| ncbi_105075546 | LRRC7 | Up |
| ncbi_105075903 | PA2G4 | Up |
| ncbi_105076004 | DDX3X | Up |
| ncbi_105076123 | ADA2 | Up |
| ncbi_105076153 | ANO6 | Up |
| ncbi_105076243 | BIN2 | Up |
| ncbi_105076516 | CAT | Up |
| ncbi_105076611 | COPB1 | Up |
| ncbi_105076634 | AMPD3 | Up |
| ncbi_105077369 | IGF2R | Up |
| ncbi_105077509 | CD68 | Up |
| ncbi_105077917 | NIT2 | Up |
| ncbi_105078012 | TRPM2 | Up |
| ncbi_105078121 | CNN2 | Up |
| ncbi_105078305 | PRAM1 | Up |
| ncbi_105078354 | ARHGAP45 | Up |
| ncbi_105078429 | PYGL | Up |
| ncbi_105078745 | B4GALT1 | Up |
| ncbi_105079106 | COTL1 | Up |
| ncbi_105079128 | CRISPLD2 | Up |
| ncbi_105079303 | MOSPD2 | Up |
| ncbi_105079662 | RAP2C | Up |
| ncbi_105080129 | ACLY | Up |
| ncbi_105080320 | NFASC | Up |
| ncbi_105080513 | ADAM10 | Up |
| ncbi_105080563 | SIGLEC14 | Up |
| ncbi_105080606 | HVCN1 | Up |
| ncbi_105080846 | P2RX1 | Up |
| ncbi_105080899 | Gm2a | Up |
| ncbi_105081249 | LPCAT1 | Up |
| ncbi_105081268 | GYG1 | Up |
| ncbi_105081341 | RAP1B | Up |
| ncbi_105081345 | CAND1 | Up |
| ncbi_105081356 | GNS | Up |
| ncbi_105081466 | GGH | Up |
| ncbi_105081524 | Huwe1 | Up |
| ncbi_105081536 | CD47 | Up |
| ncbi_105081946 | PSMD14 | Up |
| ncbi_105082395 | ITGAX | Up |
| ncbi_105082407 | SIGLEC14 | Up |
| ncbi_105082432 | ARHGAP9 | Up |
| ncbi_105082635 | DOCK2 | Up |
| ncbi_105082646 | STK10 | Up |
| ncbi_105082772 | ITGAX | Up |
| ncbi_105083082 | TMEM30A | Up |
| ncbi_105083174 | Cd93 | Up |
| ncbi_105083270 | RHOG | Up |
| ncbi_105083946 | DEGS1 | Up |
| ncbi_105062412 | KMT2E | Up |
| ncbi_105065687 | CD300LB | Up |
| ncbi_105068250 | IL6R | Up |
| ncbi_105063610 | TRAF3IP1 | Up |
| ncbi_105074697 | IL2RA | Up |
| ncbi_105078966 | EIF2AK2 | Up |
| ncbi_105080798 | HTRA1 | Up |
| ncbi_105081847 | IFIT1 | Up |
| ncbi_105084176 | TRIM38 | Up |
| ncbi_105063949 | BPTF | Up |
| ncbi_105064792 | DNAJA3 | Up |
| ncbi_105065208 | NMI | Up |
| ncbi_105067593 | MMP12 | Up |
| ncbi_105067651 | CNOT7 | Up |
| ncbi_105068180 | ADAR | Up |
| ncbi_105071402 | ADCYAP1 | Up |
| ncbi_105078696 | NLRC5 | Up |
| ncbi_105078750 | NOL6 | Up |
| ncbi_105080329 | ATM | Up |
| ncbi_105081412 | Yaf2 | Up |
| ncbi_105081629 | PARP14 | Up |
| ncbi_105061760 | CTNNB1 | Up |
| ncbi_105061976 | Tsc22d3 | Up |
| ncbi_105062400 | LRRC17 | Up |
| ncbi_105062876 | Axl | Up |
| ncbi_105063097 | GLI3 | Up |
| ncbi_105063497 | RUNX3 | Up |
| ncbi_105063667 | RBM15 | Up |
| ncbi_105063844 | EMILIN1 | Up |
| ncbi_105063928 | PRKAR1A | Up |
| ncbi_105064128 | CNR1 | Up |
| ncbi_105064395 | CARTPT | Up |
| ncbi_105065309 | GLMN | Up |
| ncbi_105065889 | TAL1 | Up |
| ncbi_105066134 | Lrch1 | Up |
| ncbi_105066223 | N4BP2L2 | Up |
| ncbi_105066611 | GPR171 | Up |
| ncbi_105066884 | Cul4a | Up |
| ncbi_105067006 | KITLG | Up |
| ncbi_105067130 | PDE5A | Up |
| ncbi_105067685 | CASP3 | Up |
| ncbi_105067856 | ZBTB16 | Up |
| ncbi_105067907 | BANK1 | Up |
| ncbi_105068454 | ZBTB46 | Up |
| ncbi_105069328 | ADORA2A | Up |
| ncbi_105070130 | HMGB3 | Up |
| ncbi_105070281 | GABPA | Up |
| ncbi_105070360 | GAL | Up |
| ncbi_105070848 | CNR2 | Up |
| ncbi_105070898 | PLA2G2D | Up |
| ncbi_105070907 | NBL1 | Up |
| ncbi_105071321 | SFRP1 | Up |
| ncbi_105071346 | IDO1 | Up |
| ncbi_105071427 | TWSG1 | Up |
| ncbi_105071725 | TNFAIP8L2 | Up |
| ncbi_105071943 | MAFB | Up |
| ncbi_105072147 | Zc3h8 | Up |
| ncbi_105072334 | ADIPOQ | Up |
| ncbi_105072374 | APOD | Up |
| ncbi_105072661 | GPR18 | Up |
| ncbi_105073407 | PDCD1LG2 | Up |
| ncbi_105073466 | NFE2L2 | Up |
| ncbi_105074125 | LST1 | Up |
| ncbi_105075052 | IGF1 | Up |
| ncbi_105075226 | NCAPG2 | Up |
| ncbi_105075308 | SLIT2 | Up |
| ncbi_105075309 | SLIT2 | Up |
| ncbi_105075712 | DTX1 | Up |
| ncbi_105075813 | MEIS1 | Up |
| ncbi_105076520 | LMO2 | Up |
| ncbi_105076630 | CTR9 | Up |
| ncbi_105076867 | GREM1 | Up |
| ncbi_105076880 | MEIS2 | Up |
| ncbi_105077074 | FBN1 | Up |
| ncbi_105077449 | ZC3H12D | Up |
| ncbi_105077878 | GPER1 | Up |
| ncbi_105078109 | FSTL3 | Up |
| ncbi_105078641 | UBASH3B | Up |
| ncbi_105079473 | PIAS3 | Up |
| ncbi_105079718 | FBXW7 | Up |
| ncbi_105079747 | TMEM131L | Up |
| ncbi_105079807 | GPNMB | Up |
| ncbi_105079862 | GPR68 | Up |
| ncbi_105080498 | LEO1 | Up |
| ncbi_105080838 | TMEM176A | Up |
| ncbi_105080839 | TMEM176B | Up |
| ncbi_105081328 | FBXO7 | Up |
| ncbi_105081445 | COPS5 | Up |
| ncbi_105081557 | CD200 | Up |
| ncbi_105081578 | TIGIT | Up |
| ncbi_105081715 | LDB1 | Up |
| ncbi_105081745 | Bloc1s2 | Up |
| ncbi_105081939 | MARCHF7 | Up |
| ncbi_105082895 | CCN3 | Up |
| ncbi_105082940 | MYC | Up |
| ncbi_105082997 | PLCB1 | Up |
| ncbi_105083000 | CDK6 | Up |
| ncbi_105083572 | LRRC32 | Up |
| ncbi_105083791 | ITPKB | Up |
| ncbi_105083845 | ADGRF5 | Up |
| ncbi_105083884 | BMP5 | Up |
| ncbi_105083994 | CDC73 | Up |
| ncbi_105084187 | RIPOR2 | Up |
| ncbi_105062842 | SUPT5H | Up |
| ncbi_105064286 | CCL5 | Up |
| ncbi_105064592 | POLR3G | Up |
| ncbi_105065286 | GBP5 | Up |
| ncbi_105069028 | DHX9 | Up |
| ncbi_105071814 | ZBP1 | Up |
| ncbi_105073267 | MED1 | Up |
| ncbi_105074946 | UBE2K | Up |
| ncbi_105075256 | COCH | Up |
| ncbi_105075731 | MMP2 | Up |
| ncbi_105078332 | TRDMT1 | Up |
| ncbi_105081314 | POLR3B | Up |
| ncbi_105081628 | PARP9 | Up |
| ncbi_105083355 | HMGB2 | Up |
| MSTRG.7218 | VPREB1 | Up |
| ncbi_105063982 | STAT1 | Up |
| ncbi_105067806 | DDX6 | Up |
| ncbi_105069203 | PML | Up |
| ncbi_105069779 | GH1 | Up |
| ncbi_105070291 | PACS1 | Up |
| ncbi_105073202 | Apobec3 | Up |
| ncbi_105076189 | DDX23 | Up |
| ncbi_105076504 | TRIM44 | Up |
| ncbi_105077700 | HERC5 | Up |
| ncbi_105079583 | SELENOK | Up |
| ncbi_105081627 | DTX3L | Up |
| ncbi_105083276 | NUP98 | Up |
| ncbi_105062297 | KRAS | Up |
| ncbi_105064618 | JAK1 | Up |
| ncbi_105065593 | SOCS3 | Up |
| ncbi_105068674 | ABCE1 | Up |
| ncbi_105069511 | PIAS1 | Up |
| ncbi_105070115 | FADS3 | Up |
| ncbi_105070236 | IFNAR2 | Up |
| ncbi_105071851 | PTPN1 | Up |
| ncbi_105073500 | SAMHD1 | Up |
| ncbi_105077267 | IFNGR1 | Up |
| ncbi_105080590 | PTPN11 | Up |
| ncbi_105082290 | SUMO1 | Up |
| ncbi_105080009 | BCL11B | Up |
| ncbi_105080211 | DCAF1 | Up |
| ncbi_105082121 | Tcf7 | Up |
| ncbi_105077472 | BCL6B | Up |
