# Supplementary material for: Integrated omics analysis reveals the immunologic characteristics of cystic Peyer’s patches in the cecum of Bactrian camels
Source: PeerJ. 2023 Jan 9;11:e14647. doi: 10.7717/peerj.14647 (PMC9835693; doi:10.7717/peerj.14647)
Supplement: Table S3 [file peerj-11-14647-s003.docx]

Table S3. Detailed topological information in the co-occurrence network calculated by Cytoscape v3.9.0.

| Node | | Average Shortest PathLength | | Betweenness Centrality | | Closeness Centrality | | Clustering Coefficient | | degree | | Eccentricity | | Neighborhood Connectivity | | Topological Coefficient |
| --- | --- | --- | --- | --- | --- | --- | --- | --- | --- | --- | --- | --- | --- | --- | --- | --- |
| BTK | 2.35 | | 0.05 | | 0.43 | | 0.53 | | 226.00 | | 5.00 | | 121.38 | |  |  |
| P2RX7 | 2.35 | | 0.05 | | 0.43 | | 0.53 | | 226.00 | | 5.00 | | 121.38 | |  |  |
| Pax5 | 2.35 | | 0.05 | | 0.43 | | 0.53 | | 226.00 | | 5.00 | | 121.38 | |  |  |
| DSG1 | 2.35 | | 0.05 | | 0.43 | | 0.53 | | 226.00 | | 5.00 | | 121.38 | |  |  |
| PTPN2 | 2.32 | | 0.46 | | 0.43 | | 0.53 | | 226.00 | | 6.00 | | 126.60 | |  |  |
| DOCK11 | 2.35 | | 0.05 | | 0.43 | | 0.53 | | 226.00 | | 5.00 | | 121.38 | |  |  |
| TBX21 | 2.35 | | 0.05 | | 0.43 | | 0.53 | | 226.00 | | 5.00 | | 121.38 | |  |  |
| IL10 | 2.35 | | 0.05 | | 0.43 | | 0.53 | | 226.00 | | 5.00 | | 121.38 | |  |  |
| HLA-DOB | 2.35 | | 0.05 | | 0.43 | | 0.53 | | 226.00 | | 5.00 | | 121.38 | |  |  |
| CENPE | 2.46 | | 0.17 | | 0.41 | | 0.59 | | 195.00 | | 6.00 | | 119.97 | |  |  |
| RAB3C | 2.81 | | 0.03 | | 0.36 | | 0.44 | | 161.00 | | 7.00 | | 74.84 | |  |  |
| ACTB | 2.81 | | 0.03 | | 0.36 | | 0.44 | | 161.00 | | 7.00 | | 74.84 | |  |  |
| MPO | 2.81 | | 0.03 | | 0.36 | | 0.44 | | 161.00 | | 7.00 | | 74.84 | |  |  |
| BCL11B | 2.81 | | 0.03 | | 0.36 | | 0.44 | | 161.00 | | 7.00 | | 74.84 | |  |  |
| IL12RB1 | 2.81 | | 0.03 | | 0.36 | | 0.44 | | 161.00 | | 7.00 | | 74.84 | |  |  |
| CTLA4 | 2.81 | | 0.03 | | 0.36 | | 0.44 | | 161.00 | | 7.00 | | 74.84 | |  |  |
| SIGLEC11 | 2.81 | | 0.03 | | 0.36 | | 0.44 | | 161.00 | | 7.00 | | 74.84 | |  |  |
| PDCD1 | 2.81 | | 0.03 | | 0.36 | | 0.44 | | 161.00 | | 7.00 | | 74.84 | |  |  |
| PGM2 | 2.67 | | 0.00 | | 0.37 | | 1.00 | | 142.00 | | 6.00 | | 147.70 | |  |  |
| IL18R1 | 2.67 | | 0.00 | | 0.37 | | 1.00 | | 142.00 | | 6.00 | | 147.70 | |  |  |
| MAP3K7 | 2.67 | | 0.00 | | 0.37 | | 1.00 | | 142.00 | | 6.00 | | 147.70 | |  |  |
| TFRC | 2.67 | | 0.00 | | 0.37 | | 1.00 | | 142.00 | | 6.00 | | 147.70 | |  |  |
| HMGB3 | 2.67 | | 0.00 | | 0.37 | | 1.00 | | 142.00 | | 6.00 | | 147.70 | |  |  |
| TLR4 | 2.67 | | 0.00 | | 0.37 | | 1.00 | | 142.00 | | 6.00 | | 147.70 | |  |  |
| LNPEP | 2.67 | | 0.00 | | 0.37 | | 1.00 | | 142.00 | | 6.00 | | 147.70 | |  |  |
| CCT2 | 2.67 | | 0.00 | | 0.37 | | 1.00 | | 142.00 | | 6.00 | | 147.70 | |  |  |
| PARP1 | 2.67 | | 0.00 | | 0.37 | | 1.00 | | 142.00 | | 6.00 | | 147.70 | |  |  |
| ADAM17 | 2.67 | | 0.00 | | 0.37 | | 1.00 | | 142.00 | | 6.00 | | 147.70 | |  |  |
| Bloc1s2 | 2.67 | | 0.00 | | 0.37 | | 1.00 | | 142.00 | | 6.00 | | 147.70 | |  |  |
| LCP1 | 2.67 | | 0.00 | | 0.37 | | 1.00 | | 142.00 | | 6.00 | | 147.70 | |  |  |
| Rab29 | 2.67 | | 0.00 | | 0.37 | | 1.00 | | 142.00 | | 6.00 | | 147.70 | |  |  |
| PSMC2 | 2.67 | | 0.00 | | 0.37 | | 1.00 | | 142.00 | | 6.00 | | 147.70 | |  |  |
| CR2 | 2.67 | | 0.00 | | 0.37 | | 1.00 | | 142.00 | | 6.00 | | 147.70 | |  |  |
| ARF6 | 2.67 | | 0.00 | | 0.37 | | 1.00 | | 142.00 | | 6.00 | | 147.70 | |  |  |
| SFPQ | 2.67 | | 0.00 | | 0.37 | | 1.00 | | 142.00 | | 6.00 | | 147.70 | |  |  |
| DHX9 | 2.67 | | 0.00 | | 0.37 | | 1.00 | | 142.00 | | 6.00 | | 147.70 | |  |  |
| Elmo2 | 2.67 | | 0.00 | | 0.37 | | 1.00 | | 142.00 | | 6.00 | | 147.70 | |  |  |
| Patr-A | 2.67 | | 0.00 | | 0.37 | | 1.00 | | 142.00 | | 6.00 | | 147.70 | |  |  |
| PSMD1 | 2.67 | | 0.00 | | 0.37 | | 1.00 | | 142.00 | | 6.00 | | 147.70 | |  |  |
| NOD1 | 2.67 | | 0.00 | | 0.37 | | 1.00 | | 142.00 | | 6.00 | | 147.70 | |  |  |
| HLA-DMB | 2.67 | | 0.00 | | 0.37 | | 1.00 | | 142.00 | | 6.00 | | 147.70 | |  |  |
| Kpnb1 | 2.67 | | 0.00 | | 0.37 | | 1.00 | | 142.00 | | 6.00 | | 147.70 | |  |  |
| KRAS | 2.67 | | 0.00 | | 0.37 | | 1.00 | | 142.00 | | 6.00 | | 147.70 | |  |  |
| DPP4 | 2.67 | | 0.00 | | 0.37 | | 1.00 | | 142.00 | | 6.00 | | 147.70 | |  |  |
| Nfkbid | 2.67 | | 0.00 | | 0.37 | | 1.00 | | 142.00 | | 6.00 | | 147.70 | |  |  |
| TEK | 2.67 | | 0.00 | | 0.37 | | 1.00 | | 142.00 | | 6.00 | | 147.70 | |  |  |
| ELMO1 | 2.67 | | 0.00 | | 0.37 | | 1.00 | | 142.00 | | 6.00 | | 147.70 | |  |  |
| AP3B1 | 2.67 | | 0.00 | | 0.37 | | 1.00 | | 142.00 | | 6.00 | | 147.70 | |  |  |
| HLA-DRB1 | 2.67 | | 0.00 | | 0.37 | | 1.00 | | 142.00 | | 6.00 | | 147.70 | |  |  |
| BIRC3 | 2.67 | | 0.00 | | 0.37 | | 1.00 | | 142.00 | | 6.00 | | 147.70 | |  |  |
| ERAP2 | 2.67 | | 0.00 | | 0.37 | | 1.00 | | 142.00 | | 6.00 | | 147.70 | |  |  |
| POLR3G | 2.67 | | 0.00 | | 0.37 | | 1.00 | | 142.00 | | 6.00 | | 147.70 | |  |  |
| HVCN1 | 2.67 | | 0.00 | | 0.37 | | 1.00 | | 142.00 | | 6.00 | | 147.70 | |  |  |
| KIF11 | 2.67 | | 0.00 | | 0.37 | | 1.00 | | 142.00 | | 6.00 | | 147.70 | |  |  |
| ARID5A | 2.67 | | 0.00 | | 0.37 | | 1.00 | | 142.00 | | 6.00 | | 147.70 | |  |  |
| ELF1 | 2.67 | | 0.00 | | 0.37 | | 1.00 | | 142.00 | | 6.00 | | 147.70 | |  |  |
| ARSB | 2.67 | | 0.00 | | 0.37 | | 1.00 | | 142.00 | | 6.00 | | 147.70 | |  |  |
| CD83 | 2.67 | | 0.00 | | 0.37 | | 1.00 | | 142.00 | | 6.00 | | 147.70 | |  |  |
| Pum2 | 2.67 | | 0.00 | | 0.37 | | 1.00 | | 142.00 | | 6.00 | | 147.70 | |  |  |
| NCKAP1L | 2.67 | | 0.00 | | 0.37 | | 1.00 | | 142.00 | | 6.00 | | 147.70 | |  |  |
| SAE1 | 2.67 | | 0.00 | | 0.37 | | 1.00 | | 142.00 | | 6.00 | | 147.70 | |  |  |
| ICOSLG | 2.67 | | 0.00 | | 0.37 | | 1.00 | | 142.00 | | 6.00 | | 147.70 | |  |  |
| TBC1D10C | 2.67 | | 0.00 | | 0.37 | | 1.00 | | 142.00 | | 6.00 | | 147.70 | |  |  |
| Wipf1 | 2.67 | | 0.00 | | 0.37 | | 1.00 | | 142.00 | | 6.00 | | 147.70 | |  |  |
| MYO1G | 2.67 | | 0.00 | | 0.37 | | 1.00 | | 142.00 | | 6.00 | | 147.70 | |  |  |
| Uba2 | 2.67 | | 0.00 | | 0.37 | | 1.00 | | 142.00 | | 6.00 | | 147.70 | |  |  |
| LRMP | 2.67 | | 0.00 | | 0.37 | | 1.00 | | 142.00 | | 6.00 | | 147.70 | |  |  |
| ZBTB1 | 2.67 | | 0.00 | | 0.37 | | 1.00 | | 142.00 | | 6.00 | | 147.70 | |  |  |
| CDC42 | 2.67 | | 0.00 | | 0.37 | | 1.00 | | 142.00 | | 6.00 | | 147.70 | |  |  |
| SEC24A | 2.67 | | 0.00 | | 0.37 | | 1.00 | | 142.00 | | 6.00 | | 147.70 | |  |  |
| JAK1 | 2.67 | | 0.00 | | 0.37 | | 1.00 | | 142.00 | | 6.00 | | 147.70 | |  |  |
| HMGB1 | 2.67 | | 0.00 | | 0.37 | | 1.00 | | 142.00 | | 6.00 | | 147.70 | |  |  |
| PARP14 | 2.67 | | 0.00 | | 0.37 | | 1.00 | | 142.00 | | 6.00 | | 147.70 | |  |  |
| NUGGC | 2.67 | | 0.00 | | 0.37 | | 1.00 | | 142.00 | | 6.00 | | 147.70 | |  |  |
| ITPKB | 2.67 | | 0.00 | | 0.37 | | 1.00 | | 142.00 | | 6.00 | | 147.70 | |  |  |
| STX11 | 2.67 | | 0.00 | | 0.37 | | 1.00 | | 142.00 | | 6.00 | | 147.70 | |  |  |
| RABGEF1 | 2.67 | | 0.00 | | 0.37 | | 1.00 | | 142.00 | | 6.00 | | 147.70 | |  |  |
| ATAD5 | 2.67 | | 0.00 | | 0.37 | | 1.00 | | 142.00 | | 6.00 | | 147.70 | |  |  |
| PTPN11 | 2.67 | | 0.00 | | 0.37 | | 1.00 | | 142.00 | | 6.00 | | 147.70 | |  |  |
| Kars1 | 2.67 | | 0.00 | | 0.37 | | 1.00 | | 142.00 | | 6.00 | | 147.70 | |  |  |
| Rab8b | 2.67 | | 0.00 | | 0.37 | | 1.00 | | 142.00 | | 6.00 | | 147.70 | |  |  |
| STAT1 | 2.67 | | 0.00 | | 0.37 | | 1.00 | | 142.00 | | 6.00 | | 147.70 | |  |  |
| ARPC2 | 2.67 | | 0.00 | | 0.37 | | 1.00 | | 142.00 | | 6.00 | | 147.70 | |  |  |
| MARCHF1 | 2.67 | | 0.00 | | 0.37 | | 1.00 | | 142.00 | | 6.00 | | 147.70 | |  |  |
| RAP1B | 2.67 | | 0.00 | | 0.37 | | 1.00 | | 142.00 | | 6.00 | | 147.70 | |  |  |
| CD68 | 2.67 | | 0.00 | | 0.37 | | 1.00 | | 142.00 | | 6.00 | | 147.70 | |  |  |
| TLR10 | 2.67 | | 0.00 | | 0.37 | | 1.00 | | 142.00 | | 6.00 | | 147.70 | |  |  |
| RIF1 | 2.67 | | 0.00 | | 0.37 | | 1.00 | | 142.00 | | 6.00 | | 147.70 | |  |  |
| Slc39a10 | 2.67 | | 0.00 | | 0.37 | | 1.00 | | 142.00 | | 6.00 | | 147.70 | |  |  |
| CBLB | 2.67 | | 0.00 | | 0.37 | | 1.00 | | 142.00 | | 6.00 | | 147.70 | |  |  |
| Sec22b | 2.67 | | 0.00 | | 0.37 | | 1.00 | | 142.00 | | 6.00 | | 147.70 | |  |  |
| PTPRC | 2.67 | | 0.00 | | 0.37 | | 1.00 | | 142.00 | | 6.00 | | 147.70 | |  |  |
| TRIM38 | 2.67 | | 0.00 | | 0.37 | | 1.00 | | 142.00 | | 6.00 | | 147.70 | |  |  |
| Tab2 | 2.67 | | 0.00 | | 0.37 | | 1.00 | | 142.00 | | 6.00 | | 147.70 | |  |  |
| SWAP70 | 2.67 | | 0.00 | | 0.37 | | 1.00 | | 142.00 | | 6.00 | | 147.70 | |  |  |
| STX7 | 2.67 | | 0.00 | | 0.37 | | 1.00 | | 142.00 | | 6.00 | | 147.70 | |  |  |
| ERP44 | 2.67 | | 0.00 | | 0.37 | | 1.00 | | 142.00 | | 6.00 | | 147.70 | |  |  |
| TAP1 | 2.67 | | 0.00 | | 0.37 | | 1.00 | | 142.00 | | 6.00 | | 147.70 | |  |  |
| PIK3C3 | 2.67 | | 0.00 | | 0.37 | | 1.00 | | 142.00 | | 6.00 | | 147.70 | |  |  |
| RFTN1 | 2.67 | | 0.00 | | 0.37 | | 1.00 | | 142.00 | | 6.00 | | 147.70 | |  |  |
| UBQLN1 | 2.67 | | 0.00 | | 0.37 | | 1.00 | | 142.00 | | 6.00 | | 147.70 | |  |  |
| MSH2 | 2.67 | | 0.00 | | 0.37 | | 1.00 | | 142.00 | | 6.00 | | 147.70 | |  |  |
| TEC | 2.67 | | 0.00 | | 0.37 | | 1.00 | | 142.00 | | 6.00 | | 147.70 | |  |  |
| GABPA | 2.67 | | 0.00 | | 0.37 | | 1.00 | | 142.00 | | 6.00 | | 147.70 | |  |  |
| PAFAH1B2 | 2.67 | | 0.00 | | 0.37 | | 1.00 | | 142.00 | | 6.00 | | 147.70 | |  |  |
| CD53 | 2.67 | | 0.00 | | 0.37 | | 1.00 | | 142.00 | | 6.00 | | 147.70 | |  |  |
| CCT8 | 2.67 | | 0.00 | | 0.37 | | 1.00 | | 142.00 | | 6.00 | | 147.70 | |  |  |
| MSH6 | 2.67 | | 0.00 | | 0.37 | | 1.00 | | 142.00 | | 6.00 | | 147.70 | |  |  |
| CTR9 | 2.67 | | 0.00 | | 0.37 | | 1.00 | | 142.00 | | 6.00 | | 147.70 | |  |  |
| Cd93 | 2.67 | | 0.00 | | 0.37 | | 1.00 | | 142.00 | | 6.00 | | 147.70 | |  |  |
| Lrch1 | 2.67 | | 0.00 | | 0.37 | | 1.00 | | 142.00 | | 6.00 | | 147.70 | |  |  |
| IL18 | 2.67 | | 0.00 | | 0.37 | | 1.00 | | 142.00 | | 6.00 | | 147.70 | |  |  |
| OSTF1 | 2.67 | | 0.00 | | 0.37 | | 1.00 | | 142.00 | | 6.00 | | 147.70 | |  |  |
| NRAS | 2.67 | | 0.00 | | 0.37 | | 1.00 | | 142.00 | | 6.00 | | 147.70 | |  |  |
| CNOT7 | 2.67 | | 0.00 | | 0.37 | | 1.00 | | 142.00 | | 6.00 | | 147.70 | |  |  |
| KIF15 | 2.67 | | 0.00 | | 0.37 | | 1.00 | | 142.00 | | 6.00 | | 147.70 | |  |  |
| PLCG2 | 2.67 | | 0.00 | | 0.37 | | 1.00 | | 142.00 | | 6.00 | | 147.70 | |  |  |
| PELI1 | 2.67 | | 0.00 | | 0.37 | | 1.00 | | 142.00 | | 6.00 | | 147.70 | |  |  |
| NECTIN2 | 2.67 | | 0.00 | | 0.37 | | 1.00 | | 142.00 | | 6.00 | | 147.70 | |  |  |
| Pou2f2 | 2.67 | | 0.00 | | 0.37 | | 1.00 | | 142.00 | | 6.00 | | 147.70 | |  |  |
| CAPZA1 | 2.67 | | 0.00 | | 0.37 | | 1.00 | | 142.00 | | 6.00 | | 147.70 | |  |  |
| Snap23 | 2.67 | | 0.00 | | 0.37 | | 1.00 | | 142.00 | | 6.00 | | 147.70 | |  |  |
| DNAJC13 | 2.67 | | 0.00 | | 0.37 | | 1.00 | | 142.00 | | 6.00 | | 147.70 | |  |  |
| Traf3 | 2.67 | | 0.00 | | 0.37 | | 1.00 | | 142.00 | | 6.00 | | 147.70 | |  |  |
| DDX6 | 2.67 | | 0.00 | | 0.37 | | 1.00 | | 142.00 | | 6.00 | | 147.70 | |  |  |
| ABCE1 | 2.67 | | 0.00 | | 0.37 | | 1.00 | | 142.00 | | 6.00 | | 147.70 | |  |  |
| HSP90B1 | 2.67 | | 0.00 | | 0.37 | | 1.00 | | 142.00 | | 6.00 | | 147.70 | |  |  |
| STK10 | 2.67 | | 0.00 | | 0.37 | | 1.00 | | 142.00 | | 6.00 | | 147.70 | |  |  |
| KIF2A | 2.67 | | 0.00 | | 0.37 | | 1.00 | | 142.00 | | 6.00 | | 147.70 | |  |  |
| HSPD1 | 2.67 | | 0.00 | | 0.37 | | 1.00 | | 142.00 | | 6.00 | | 147.70 | |  |  |
| CTSC | 2.67 | | 0.00 | | 0.37 | | 1.00 | | 142.00 | | 6.00 | | 147.70 | |  |  |
| CAPZB | 2.67 | | 0.00 | | 0.37 | | 1.00 | | 142.00 | | 6.00 | | 147.70 | |  |  |
| STXBP1 | 2.67 | | 0.00 | | 0.37 | | 1.00 | | 142.00 | | 6.00 | | 147.70 | |  |  |
| GYG1 | 2.67 | | 0.00 | | 0.37 | | 1.00 | | 142.00 | | 6.00 | | 147.70 | |  |  |
| TMEM30A | 2.67 | | 0.00 | | 0.37 | | 1.00 | | 142.00 | | 6.00 | | 147.70 | |  |  |
| CAND1 | 2.67 | | 0.00 | | 0.37 | | 1.00 | | 142.00 | | 6.00 | | 147.70 | |  |  |
| LRRC32 | 2.67 | | 0.00 | | 0.37 | | 1.00 | | 142.00 | | 6.00 | | 147.70 | |  |  |
| MATR3 | 2.67 | | 0.00 | | 0.37 | | 1.00 | | 142.00 | | 6.00 | | 147.70 | |  |  |
| HSP90AB1 | 2.67 | | 0.00 | | 0.37 | | 1.00 | | 142.00 | | 6.00 | | 147.70 | |  |  |
| TANK | 2.67 | | 0.00 | | 0.37 | | 1.00 | | 142.00 | | 6.00 | | 147.70 | |  |  |
| DENND1B | 2.67 | | 0.00 | | 0.37 | | 1.00 | | 142.00 | | 6.00 | | 147.70 | |  |  |
| MSH3 | 2.67 | | 0.00 | | 0.37 | | 1.00 | | 142.00 | | 6.00 | | 147.70 | |  |  |
| BIRC2 | 2.67 | | 0.00 | | 0.37 | | 1.00 | | 142.00 | | 6.00 | | 147.70 | |  |  |
| PMS2 | 2.67 | | 0.00 | | 0.37 | | 1.00 | | 142.00 | | 6.00 | | 147.70 | |  |  |
| ACTR3 | 2.67 | | 0.00 | | 0.37 | | 1.00 | | 142.00 | | 6.00 | | 147.70 | |  |  |
| PSMD12 | 2.67 | | 0.00 | | 0.37 | | 1.00 | | 142.00 | | 6.00 | | 147.70 | |  |  |
| PAXIP1 | 2.67 | | 0.00 | | 0.37 | | 1.00 | | 142.00 | | 6.00 | | 147.70 | |  |  |
| Yaf2 | 2.67 | | 0.00 | | 0.37 | | 1.00 | | 142.00 | | 6.00 | | 147.70 | |  |  |
| RAP1A | 2.67 | | 0.00 | | 0.37 | | 1.00 | | 142.00 | | 6.00 | | 147.70 | |  |  |
| KIF22 | 2.67 | | 0.00 | | 0.37 | | 1.00 | | 142.00 | | 6.00 | | 147.70 | |  |  |
| ACTR2 | 2.67 | | 0.00 | | 0.37 | | 1.00 | | 142.00 | | 6.00 | | 147.70 | |  |  |
| CNR2 | 2.84 | | 0.12 | | 0.35 | | 0.47 | | 135.00 | | 7.00 | | 70.14 | |  |  |
| CCL3 | 2.75 | | 0.21 | | 0.36 | | 0.52 | | 126.00 | | 6.00 | | 77.16 | |  |  |
| FCGR2 | 3.56 | | 0.03 | | 0.28 | | 0.50 | | 101.00 | | 8.00 | | 59.45 | |  |  |
| NFATC2 | 2.95 | | 0.02 | | 0.34 | | 0.47 | | 88.00 | | 6.00 | | 45.82 | |  |  |
| CXorf21 | 2.95 | | 0.02 | | 0.34 | | 0.47 | | 88.00 | | 6.00 | | 45.82 | |  |  |
| RC3H1 | 2.95 | | 0.02 | | 0.34 | | 0.47 | | 88.00 | | 6.00 | | 45.82 | |  |  |
| GREM1 | 2.95 | | 0.02 | | 0.34 | | 0.47 | | 88.00 | | 6.00 | | 45.82 | |  |  |
| NFASC | 2.95 | | 0.02 | | 0.34 | | 0.47 | | 88.00 | | 6.00 | | 45.82 | |  |  |
| CD86 | 2.95 | | 0.02 | | 0.34 | | 0.47 | | 88.00 | | 6.00 | | 45.82 | |  |  |
| CD47 | 2.97 | | 0.00 | | 0.34 | | 1.00 | | 84.00 | | 7.00 | | 93.63 | |  |  |
| PRKACB | 2.97 | | 0.00 | | 0.34 | | 1.00 | | 84.00 | | 7.00 | | 93.63 | |  |  |
| CCL5 | 2.97 | | 0.00 | | 0.34 | | 1.00 | | 84.00 | | 7.00 | | 93.63 | |  |  |
| FLT3 | 2.97 | | 0.00 | | 0.34 | | 1.00 | | 84.00 | | 7.00 | | 93.63 | |  |  |
| PRKAR1A | 2.97 | | 0.00 | | 0.34 | | 1.00 | | 84.00 | | 7.00 | | 93.63 | |  |  |
| PIK3CG | 2.97 | | 0.00 | | 0.34 | | 1.00 | | 84.00 | | 7.00 | | 93.63 | |  |  |
| CAT | 2.97 | | 0.00 | | 0.34 | | 1.00 | | 84.00 | | 7.00 | | 93.63 | |  |  |
| CPNE3 | 2.97 | | 0.00 | | 0.34 | | 1.00 | | 84.00 | | 7.00 | | 93.63 | |  |  |
| THOC1 | 2.97 | | 0.00 | | 0.34 | | 1.00 | | 84.00 | | 7.00 | | 93.63 | |  |  |
| ABI1 | 2.97 | | 0.00 | | 0.34 | | 1.00 | | 84.00 | | 7.00 | | 93.63 | |  |  |
| GCNT3 | 2.97 | | 0.00 | | 0.34 | | 1.00 | | 84.00 | | 7.00 | | 93.63 | |  |  |
| ELF2 | 2.97 | | 0.00 | | 0.34 | | 1.00 | | 84.00 | | 7.00 | | 93.63 | |  |  |
| YTHDF2 | 2.97 | | 0.00 | | 0.34 | | 1.00 | | 84.00 | | 7.00 | | 93.63 | |  |  |
| S100A8 | 2.97 | | 0.00 | | 0.34 | | 1.00 | | 84.00 | | 7.00 | | 93.63 | |  |  |
| CTSL | 2.97 | | 0.00 | | 0.34 | | 1.00 | | 84.00 | | 7.00 | | 93.63 | |  |  |
| PSMB8 | 2.97 | | 0.00 | | 0.34 | | 1.00 | | 84.00 | | 7.00 | | 93.63 | |  |  |
| PIAS1 | 2.97 | | 0.00 | | 0.34 | | 1.00 | | 84.00 | | 7.00 | | 93.63 | |  |  |
| Zc3h8 | 2.97 | | 0.00 | | 0.34 | | 1.00 | | 84.00 | | 7.00 | | 93.63 | |  |  |
| PTAFR | 2.97 | | 0.00 | | 0.34 | | 1.00 | | 84.00 | | 7.00 | | 93.63 | |  |  |
| PSMD14 | 2.97 | | 0.00 | | 0.34 | | 1.00 | | 84.00 | | 7.00 | | 93.63 | |  |  |
| SLAMF1 | 2.97 | | 0.00 | | 0.34 | | 1.00 | | 84.00 | | 7.00 | | 93.63 | |  |  |
| LY9 | 2.97 | | 0.00 | | 0.34 | | 1.00 | | 84.00 | | 7.00 | | 93.63 | |  |  |
| PSPC1 | 2.97 | | 0.00 | | 0.34 | | 1.00 | | 84.00 | | 7.00 | | 93.63 | |  |  |
| PVR | 2.97 | | 0.00 | | 0.34 | | 1.00 | | 84.00 | | 7.00 | | 93.63 | |  |  |
| IL27RA | 2.97 | | 0.00 | | 0.34 | | 1.00 | | 84.00 | | 7.00 | | 93.63 | |  |  |
| KITLG | 2.97 | | 0.00 | | 0.34 | | 1.00 | | 84.00 | | 7.00 | | 93.63 | |  |  |
| FBXO7 | 2.97 | | 0.00 | | 0.34 | | 1.00 | | 84.00 | | 7.00 | | 93.63 | |  |  |
| BIN2 | 2.97 | | 0.00 | | 0.34 | | 1.00 | | 84.00 | | 7.00 | | 93.63 | |  |  |
| LCK | 2.97 | | 0.00 | | 0.34 | | 1.00 | | 84.00 | | 7.00 | | 93.63 | |  |  |
| Pak2 | 2.97 | | 0.00 | | 0.34 | | 1.00 | | 84.00 | | 7.00 | | 93.63 | |  |  |
| NFE2L2 | 2.97 | | 0.00 | | 0.34 | | 1.00 | | 84.00 | | 7.00 | | 93.63 | |  |  |
| RARRES2 | 2.97 | | 0.00 | | 0.34 | | 1.00 | | 84.00 | | 7.00 | | 93.63 | |  |  |
| HLA-B | 2.97 | | 0.00 | | 0.34 | | 1.00 | | 84.00 | | 7.00 | | 93.63 | |  |  |
| UNC13D | 2.97 | | 0.00 | | 0.34 | | 1.00 | | 84.00 | | 7.00 | | 93.63 | |  |  |
| RAP2C | 2.97 | | 0.00 | | 0.34 | | 1.00 | | 84.00 | | 7.00 | | 93.63 | |  |  |
| IL16 | 2.97 | | 0.00 | | 0.34 | | 1.00 | | 84.00 | | 7.00 | | 93.63 | |  |  |
| ARMC8 | 2.97 | | 0.00 | | 0.34 | | 1.00 | | 84.00 | | 7.00 | | 93.63 | |  |  |
| Tbk1 | 2.97 | | 0.00 | | 0.34 | | 1.00 | | 84.00 | | 7.00 | | 93.63 | |  |  |
| Faf1 | 2.97 | | 0.00 | | 0.34 | | 1.00 | | 84.00 | | 7.00 | | 93.63 | |  |  |
| LSM14A | 2.97 | | 0.00 | | 0.34 | | 1.00 | | 84.00 | | 7.00 | | 93.63 | |  |  |
| Ly86 | 2.97 | | 0.00 | | 0.34 | | 1.00 | | 84.00 | | 7.00 | | 93.63 | |  |  |
| NFKB2 | 2.97 | | 0.00 | | 0.34 | | 1.00 | | 84.00 | | 7.00 | | 93.63 | |  |  |
| DCLRE1C | 2.97 | | 0.00 | | 0.34 | | 1.00 | | 84.00 | | 7.00 | | 93.63 | |  |  |
| CD40LG | 2.97 | | 0.00 | | 0.34 | | 1.00 | | 84.00 | | 7.00 | | 93.63 | |  |  |
| SLAMF7 | 2.97 | | 0.00 | | 0.34 | | 1.00 | | 84.00 | | 7.00 | | 93.63 | |  |  |
| RSAD2 | 2.97 | | 0.00 | | 0.34 | | 1.00 | | 84.00 | | 7.00 | | 93.63 | |  |  |
| Apobec3 | 2.97 | | 0.00 | | 0.34 | | 1.00 | | 84.00 | | 7.00 | | 93.63 | |  |  |
| RHOG | 2.97 | | 0.00 | | 0.34 | | 1.00 | | 84.00 | | 7.00 | | 93.63 | |  |  |
| Tnfrsf4 | 2.97 | | 0.00 | | 0.34 | | 1.00 | | 84.00 | | 7.00 | | 93.63 | |  |  |
| CD3E | 2.97 | | 0.00 | | 0.34 | | 1.00 | | 84.00 | | 7.00 | | 93.63 | |  |  |
| UBE2I | 2.97 | | 0.00 | | 0.34 | | 1.00 | | 84.00 | | 7.00 | | 93.63 | |  |  |
| IGHG1 | 2.97 | | 0.00 | | 0.34 | | 1.00 | | 84.00 | | 7.00 | | 93.63 | |  |  |
| GBP5 | 2.97 | | 0.00 | | 0.34 | | 1.00 | | 84.00 | | 7.00 | | 93.63 | |  |  |
| DLG1 | 2.97 | | 0.00 | | 0.34 | | 1.00 | | 84.00 | | 7.00 | | 93.63 | |  |  |
| CXCL9 | 2.97 | | 0.00 | | 0.34 | | 1.00 | | 84.00 | | 7.00 | | 93.63 | |  |  |
| DDX23 | 2.97 | | 0.00 | | 0.34 | | 1.00 | | 84.00 | | 7.00 | | 93.63 | |  |  |
| CCL21 | 2.97 | | 0.00 | | 0.34 | | 1.00 | | 84.00 | | 7.00 | | 93.63 | |  |  |
| Tsc22d3 | 2.97 | | 0.00 | | 0.34 | | 1.00 | | 84.00 | | 7.00 | | 93.63 | |  |  |
| BMP5 | 2.97 | | 0.00 | | 0.34 | | 1.00 | | 84.00 | | 7.00 | | 93.63 | |  |  |
| OTULIN | 2.97 | | 0.00 | | 0.34 | | 1.00 | | 84.00 | | 7.00 | | 93.63 | |  |  |
| SELL | 2.97 | | 0.00 | | 0.34 | | 1.00 | | 84.00 | | 7.00 | | 93.63 | |  |  |
| STXBP3 | 2.97 | | 0.00 | | 0.34 | | 1.00 | | 84.00 | | 7.00 | | 93.63 | |  |  |
| HLA-DRB1 | 2.97 | | 0.00 | | 0.34 | | 1.00 | | 84.00 | | 7.00 | | 93.63 | |  |  |
| CAMK4 | 2.97 | | 0.00 | | 0.34 | | 1.00 | | 84.00 | | 7.00 | | 93.63 | |  |  |
| MAP3K4 | 2.97 | | 0.00 | | 0.34 | | 1.00 | | 84.00 | | 7.00 | | 93.63 | |  |  |
| AIRE | 2.97 | | 0.00 | | 0.34 | | 1.00 | | 84.00 | | 7.00 | | 93.63 | |  |  |
| TRIM27 | 2.97 | | 0.00 | | 0.34 | | 1.00 | | 84.00 | | 7.00 | | 93.63 | |  |  |
| UBE2K | 2.97 | | 0.00 | | 0.34 | | 1.00 | | 84.00 | | 7.00 | | 93.63 | |  |  |
| CD7 | 2.97 | | 0.00 | | 0.34 | | 1.00 | | 84.00 | | 7.00 | | 93.63 | |  |  |
| FYN | 2.97 | | 0.00 | | 0.34 | | 1.00 | | 84.00 | | 7.00 | | 93.63 | |  |  |
| JCHAIN | 2.97 | | 0.00 | | 0.34 | | 1.00 | | 84.00 | | 7.00 | | 93.63 | |  |  |
| MAP3K1 | 2.97 | | 0.00 | | 0.34 | | 1.00 | | 84.00 | | 7.00 | | 93.63 | |  |  |
| CDC73 | 2.97 | | 0.00 | | 0.34 | | 1.00 | | 84.00 | | 7.00 | | 93.63 | |  |  |
| PIAS3 | 2.97 | | 0.00 | | 0.34 | | 1.00 | | 84.00 | | 7.00 | | 93.63 | |  |  |
| SNX4 | 2.97 | | 0.00 | | 0.34 | | 1.00 | | 84.00 | | 7.00 | | 93.63 | |  |  |
| PTK2B | 3.03 | | 0.00 | | 0.33 | | 1.00 | | 83.00 | | 6.00 | | 97.30 | |  |  |
| Sec31a | 3.03 | | 0.00 | | 0.33 | | 1.00 | | 83.00 | | 6.00 | | 97.30 | |  |  |
| ROCK1 | 3.03 | | 0.00 | | 0.33 | | 1.00 | | 83.00 | | 6.00 | | 97.30 | |  |  |
| NEDD4 | 3.03 | | 0.00 | | 0.33 | | 1.00 | | 83.00 | | 6.00 | | 97.30 | |  |  |
| NUP98 | 3.03 | | 0.00 | | 0.33 | | 1.00 | | 83.00 | | 6.00 | | 97.30 | |  |  |
| ZC3H12D | 3.03 | | 0.00 | | 0.33 | | 1.00 | | 83.00 | | 6.00 | | 97.30 | |  |  |
| TNIP2 | 3.03 | | 0.00 | | 0.33 | | 1.00 | | 83.00 | | 6.00 | | 97.30 | |  |  |
| TKFC | 3.03 | | 0.00 | | 0.33 | | 1.00 | | 83.00 | | 6.00 | | 97.30 | |  |  |
| RAPGEF1 | 3.03 | | 0.00 | | 0.33 | | 1.00 | | 83.00 | | 6.00 | | 97.30 | |  |  |
| CNN2 | 3.03 | | 0.00 | | 0.33 | | 1.00 | | 83.00 | | 6.00 | | 97.30 | |  |  |
| TNFRSF13C | 3.03 | | 0.00 | | 0.33 | | 1.00 | | 83.00 | | 6.00 | | 97.30 | |  |  |
| CYLD | 3.03 | | 0.00 | | 0.33 | | 1.00 | | 83.00 | | 6.00 | | 97.30 | |  |  |
| KIF18A | 3.03 | | 0.00 | | 0.33 | | 1.00 | | 83.00 | | 6.00 | | 97.30 | |  |  |
| NFATC3 | 3.03 | | 0.00 | | 0.33 | | 1.00 | | 83.00 | | 6.00 | | 97.30 | |  |  |
| WAS | 3.03 | | 0.00 | | 0.33 | | 1.00 | | 83.00 | | 6.00 | | 97.30 | |  |  |
| PYCARD | 3.03 | | 0.00 | | 0.33 | | 1.00 | | 83.00 | | 6.00 | | 97.30 | |  |  |
| NUP88 | 3.03 | | 0.00 | | 0.33 | | 1.00 | | 83.00 | | 6.00 | | 97.30 | |  |  |
| RAC2 | 3.03 | | 0.00 | | 0.33 | | 1.00 | | 83.00 | | 6.00 | | 97.30 | |  |  |
| CCL2 | 3.03 | | 0.00 | | 0.33 | | 1.00 | | 83.00 | | 6.00 | | 97.30 | |  |  |
| KMT2E | 3.03 | | 0.00 | | 0.33 | | 1.00 | | 83.00 | | 6.00 | | 97.30 | |  |  |
| CD79B | 3.03 | | 0.00 | | 0.33 | | 1.00 | | 83.00 | | 6.00 | | 97.30 | |  |  |
| Cyfip2 | 3.03 | | 0.00 | | 0.33 | | 1.00 | | 83.00 | | 6.00 | | 97.30 | |  |  |
| VCP | 3.03 | | 0.00 | | 0.33 | | 1.00 | | 83.00 | | 6.00 | | 97.30 | |  |  |
| TUBB5 | 3.03 | | 0.00 | | 0.33 | | 1.00 | | 83.00 | | 6.00 | | 97.30 | |  |  |
| CORO1A | 3.03 | | 0.00 | | 0.33 | | 1.00 | | 83.00 | | 6.00 | | 97.30 | |  |  |
| NR1H4 | 3.43 | | 0.02 | | 0.29 | | 0.43 | | 83.00 | | 7.00 | | 43.02 | |  |  |
| HSPA8 | 3.03 | | 0.00 | | 0.33 | | 1.00 | | 83.00 | | 6.00 | | 97.30 | |  |  |
| TGFB1 | 3.03 | | 0.00 | | 0.33 | | 1.00 | | 83.00 | | 6.00 | | 97.30 | |  |  |
| SEMA4A | 3.03 | | 0.00 | | 0.33 | | 1.00 | | 83.00 | | 6.00 | | 97.30 | |  |  |
| Cadm1 | 3.03 | | 0.00 | | 0.33 | | 1.00 | | 83.00 | | 6.00 | | 97.30 | |  |  |
| MALT1 | 3.03 | | 0.00 | | 0.33 | | 1.00 | | 83.00 | | 6.00 | | 97.30 | |  |  |
| MUC12 | 3.03 | | 0.00 | | 0.33 | | 1.00 | | 83.00 | | 6.00 | | 97.30 | |  |  |
| SASH3 | 3.03 | | 0.00 | | 0.33 | | 1.00 | | 83.00 | | 6.00 | | 97.30 | |  |  |
| PA2G4 | 3.03 | | 0.00 | | 0.33 | | 1.00 | | 83.00 | | 6.00 | | 97.30 | |  |  |
| LAMP3 | 3.03 | | 0.00 | | 0.33 | | 1.00 | | 83.00 | | 6.00 | | 97.30 | |  |  |
| VAV1 | 3.03 | | 0.00 | | 0.33 | | 1.00 | | 83.00 | | 6.00 | | 97.30 | |  |  |
| UNG | 3.03 | | 0.00 | | 0.33 | | 1.00 | | 83.00 | | 6.00 | | 97.30 | |  |  |
| Cul4a | 3.03 | | 0.00 | | 0.33 | | 1.00 | | 83.00 | | 6.00 | | 97.30 | |  |  |
| ITGAL | 3.03 | | 0.00 | | 0.33 | | 1.00 | | 83.00 | | 6.00 | | 97.30 | |  |  |
| Cd180 | 3.03 | | 0.00 | | 0.33 | | 1.00 | | 83.00 | | 6.00 | | 97.30 | |  |  |
| OTUD4 | 3.03 | | 0.00 | | 0.33 | | 1.00 | | 83.00 | | 6.00 | | 97.30 | |  |  |
| PRKCQ | 3.03 | | 0.00 | | 0.33 | | 1.00 | | 83.00 | | 6.00 | | 97.30 | |  |  |
| GALNT2 | 3.03 | | 0.00 | | 0.33 | | 1.00 | | 83.00 | | 6.00 | | 97.30 | |  |  |
| COTL1 | 3.03 | | 0.00 | | 0.33 | | 1.00 | | 83.00 | | 6.00 | | 97.30 | |  |  |
| Dok3 | 3.03 | | 0.00 | | 0.33 | | 1.00 | | 83.00 | | 6.00 | | 97.30 | |  |  |
| HLA-DOA | 3.03 | | 0.00 | | 0.33 | | 1.00 | | 83.00 | | 6.00 | | 97.30 | |  |  |
| C3AR1 | 3.03 | | 0.00 | | 0.33 | | 1.00 | | 83.00 | | 6.00 | | 97.30 | |  |  |
| SEC24D | 3.03 | | 0.00 | | 0.33 | | 1.00 | | 83.00 | | 6.00 | | 97.30 | |  |  |
| POLM | 3.03 | | 0.00 | | 0.33 | | 1.00 | | 83.00 | | 6.00 | | 97.30 | |  |  |
| GPNMB | 3.03 | | 0.00 | | 0.33 | | 1.00 | | 83.00 | | 6.00 | | 97.30 | |  |  |
| CLU | 3.03 | | 0.00 | | 0.33 | | 1.00 | | 83.00 | | 6.00 | | 97.30 | |  |  |
| MLH1 | 3.03 | | 0.00 | | 0.33 | | 1.00 | | 83.00 | | 6.00 | | 97.30 | |  |  |
| SIT1 | 3.03 | | 0.00 | | 0.33 | | 1.00 | | 83.00 | | 6.00 | | 97.30 | |  |  |
| USP2 | 3.03 | | 0.00 | | 0.33 | | 1.00 | | 83.00 | | 6.00 | | 97.30 | |  |  |
| CLTC | 3.03 | | 0.00 | | 0.33 | | 1.00 | | 83.00 | | 6.00 | | 97.30 | |  |  |
| NCAPG2 | 3.03 | | 0.00 | | 0.33 | | 1.00 | | 83.00 | | 6.00 | | 97.30 | |  |  |
| BPTF | 3.03 | | 0.00 | | 0.33 | | 1.00 | | 83.00 | | 6.00 | | 97.30 | |  |  |
| NFKB1 | 3.03 | | 0.00 | | 0.33 | | 1.00 | | 83.00 | | 6.00 | | 97.30 | |  |  |
| STK11 | 3.03 | | 0.00 | | 0.33 | | 1.00 | | 83.00 | | 6.00 | | 97.30 | |  |  |
| PAG1 | 3.03 | | 0.00 | | 0.33 | | 1.00 | | 83.00 | | 6.00 | | 97.30 | |  |  |
| SLAMF6 | 3.03 | | 0.00 | | 0.33 | | 1.00 | | 83.00 | | 6.00 | | 97.30 | |  |  |
| EP300 | 3.03 | | 0.00 | | 0.33 | | 1.00 | | 83.00 | | 6.00 | | 97.30 | |  |  |
| AMPD3 | 3.03 | | 0.00 | | 0.33 | | 1.00 | | 83.00 | | 6.00 | | 97.30 | |  |  |
| SARM1 | 3.03 | | 0.00 | | 0.33 | | 1.00 | | 83.00 | | 6.00 | | 97.30 | |  |  |
| FCER1A | 3.03 | | 0.00 | | 0.33 | | 1.00 | | 83.00 | | 6.00 | | 97.30 | |  |  |
| RNF19B | 3.03 | | 0.00 | | 0.33 | | 1.00 | | 83.00 | | 6.00 | | 97.30 | |  |  |
| INPP5D | 3.03 | | 0.00 | | 0.33 | | 1.00 | | 83.00 | | 6.00 | | 97.30 | |  |  |
| CD300A | 3.03 | | 0.00 | | 0.33 | | 1.00 | | 83.00 | | 6.00 | | 97.30 | |  |  |
| EXO1 | 3.03 | | 0.00 | | 0.33 | | 1.00 | | 83.00 | | 6.00 | | 97.30 | |  |  |
| PTPN1 | 3.03 | | 0.00 | | 0.33 | | 1.00 | | 83.00 | | 6.00 | | 97.30 | |  |  |
| RUNX1 | 3.03 | | 0.00 | | 0.33 | | 1.00 | | 83.00 | | 6.00 | | 97.30 | |  |  |
| DCTN5 | 3.03 | | 0.00 | | 0.33 | | 1.00 | | 83.00 | | 6.00 | | 97.30 | |  |  |
| SIRT1 | 3.03 | | 0.00 | | 0.33 | | 1.00 | | 83.00 | | 6.00 | | 97.30 | |  |  |
| Pou2af1 | 3.03 | | 0.00 | | 0.33 | | 1.00 | | 83.00 | | 6.00 | | 97.30 | |  |  |
| HLA-DRA | 3.03 | | 0.00 | | 0.33 | | 1.00 | | 83.00 | | 6.00 | | 97.30 | |  |  |
| IFNAR2 | 3.03 | | 0.00 | | 0.33 | | 1.00 | | 83.00 | | 6.00 | | 97.30 | |  |  |
| KLRF2 | 4.57 | | 0.05 | | 0.22 | | 0.47 | | 73.00 | | 8.00 | | 36.08 | |  |  |
| TRAV8-3 | 4.57 | | 0.05 | | 0.22 | | 0.47 | | 73.00 | | 8.00 | | 36.08 | |  |  |
| PLCG1 | 4.57 | | 0.05 | | 0.22 | | 0.47 | | 73.00 | | 8.00 | | 36.08 | |  |  |
| TIGIT | 3.32 | | 0.01 | | 0.30 | | 0.48 | | 72.00 | | 7.00 | | 38.97 | |  |  |
| GCSAM | 3.32 | | 0.01 | | 0.30 | | 0.48 | | 72.00 | | 7.00 | | 38.97 | |  |  |
| CFH | 3.32 | | 0.01 | | 0.30 | | 0.48 | | 72.00 | | 7.00 | | 38.97 | |  |  |
| CD1B | 3.32 | | 0.01 | | 0.30 | | 0.48 | | 72.00 | | 7.00 | | 38.97 | |  |  |
| LTF | 3.32 | | 0.01 | | 0.30 | | 0.48 | | 72.00 | | 7.00 | | 38.97 | |  |  |
| FFAR3 | 3.32 | | 0.01 | | 0.30 | | 0.48 | | 72.00 | | 7.00 | | 38.97 | |  |  |
| IGLV1-40 | 3.32 | | 0.01 | | 0.30 | | 0.48 | | 72.00 | | 7.00 | | 38.97 | |  |  |
| TGFB3 | 3.65 | | 0.01 | | 0.27 | | 0.37 | | 67.00 | | 8.00 | | 31.78 | |  |  |
| TESPA1 | 3.65 | | 0.01 | | 0.27 | | 0.37 | | 67.00 | | 8.00 | | 31.78 | |  |  |
| CR1 | 3.65 | | 0.01 | | 0.27 | | 0.37 | | 67.00 | | 8.00 | | 31.78 | |  |  |
| SH3GL2 | 3.65 | | 0.01 | | 0.27 | | 0.37 | | 67.00 | | 8.00 | | 31.78 | |  |  |
| CRTAM | 3.65 | | 0.01 | | 0.27 | | 0.37 | | 67.00 | | 8.00 | | 31.78 | |  |  |
| PIK3R1 | 3.67 | | 0.00 | | 0.27 | | 1.00 | | 65.00 | | 8.00 | | 77.37 | |  |  |
| STAT6 | 3.67 | | 0.00 | | 0.27 | | 1.00 | | 65.00 | | 8.00 | | 77.37 | |  |  |
| DNAJA3 | 3.67 | | 0.00 | | 0.27 | | 1.00 | | 65.00 | | 8.00 | | 77.37 | |  |  |
| ADORA2A | 3.67 | | 0.00 | | 0.27 | | 1.00 | | 65.00 | | 8.00 | | 77.37 | |  |  |
| LGMN | 3.67 | | 0.00 | | 0.27 | | 1.00 | | 65.00 | | 8.00 | | 77.37 | |  |  |
| FYB1 | 3.67 | | 0.00 | | 0.27 | | 1.00 | | 65.00 | | 8.00 | | 77.37 | |  |  |
| COPS5 | 3.67 | | 0.00 | | 0.27 | | 1.00 | | 65.00 | | 8.00 | | 77.37 | |  |  |
| CD200 | 3.67 | | 0.00 | | 0.27 | | 1.00 | | 65.00 | | 8.00 | | 77.37 | |  |  |
| CXCL12 | 3.67 | | 0.00 | | 0.27 | | 1.00 | | 65.00 | | 8.00 | | 77.37 | |  |  |
| PACS1 | 3.67 | | 0.00 | | 0.27 | | 1.00 | | 65.00 | | 8.00 | | 77.37 | |  |  |
| HLA-DRB1 | 3.67 | | 0.00 | | 0.27 | | 1.00 | | 65.00 | | 8.00 | | 77.37 | |  |  |
| IDO1 | 3.67 | | 0.00 | | 0.27 | | 1.00 | | 65.00 | | 8.00 | | 77.37 | |  |  |
| CD74 | 3.67 | | 0.00 | | 0.27 | | 1.00 | | 65.00 | | 8.00 | | 77.37 | |  |  |
| MZB1 | 3.67 | | 0.00 | | 0.27 | | 1.00 | | 65.00 | | 8.00 | | 77.37 | |  |  |
| ST6GAL1 | 3.67 | | 0.00 | | 0.27 | | 1.00 | | 65.00 | | 8.00 | | 77.37 | |  |  |
| S100A9 | 3.67 | | 0.00 | | 0.27 | | 1.00 | | 65.00 | | 8.00 | | 77.37 | |  |  |
| SLA2 | 3.67 | | 0.00 | | 0.27 | | 1.00 | | 65.00 | | 8.00 | | 77.37 | |  |  |
| FCAMR | 3.67 | | 0.00 | | 0.27 | | 1.00 | | 65.00 | | 8.00 | | 77.37 | |  |  |
| DTX1 | 3.67 | | 0.00 | | 0.27 | | 1.00 | | 65.00 | | 8.00 | | 77.37 | |  |  |
| CHI3L1 | 3.67 | | 0.00 | | 0.27 | | 1.00 | | 65.00 | | 8.00 | | 77.37 | |  |  |
| FBXW7 | 3.67 | | 0.00 | | 0.27 | | 1.00 | | 65.00 | | 8.00 | | 77.37 | |  |  |
| PTPN6 | 3.67 | | 0.00 | | 0.27 | | 1.00 | | 65.00 | | 8.00 | | 77.37 | |  |  |
| CDC5L | 3.67 | | 0.00 | | 0.27 | | 1.00 | | 65.00 | | 8.00 | | 77.37 | |  |  |
| SLC44A2 | 3.67 | | 0.00 | | 0.27 | | 1.00 | | 65.00 | | 8.00 | | 77.37 | |  |  |
| ICAM3 | 3.67 | | 0.00 | | 0.27 | | 1.00 | | 65.00 | | 8.00 | | 77.37 | |  |  |
| CMTM3 | 3.67 | | 0.00 | | 0.27 | | 1.00 | | 65.00 | | 8.00 | | 77.37 | |  |  |
| ARHGAP45 | 3.67 | | 0.00 | | 0.27 | | 1.00 | | 65.00 | | 8.00 | | 77.37 | |  |  |
| KCNAB2 | 3.67 | | 0.00 | | 0.27 | | 1.00 | | 65.00 | | 8.00 | | 77.37 | |  |  |
| ORAI1 | 3.67 | | 0.00 | | 0.27 | | 1.00 | | 65.00 | | 8.00 | | 77.37 | |  |  |
| DEGS1 | 3.67 | | 0.00 | | 0.27 | | 1.00 | | 65.00 | | 8.00 | | 77.37 | |  |  |
| TP53BP1 | 3.67 | | 0.00 | | 0.27 | | 1.00 | | 65.00 | | 8.00 | | 77.37 | |  |  |
| HLA-DMA | 3.67 | | 0.00 | | 0.27 | | 1.00 | | 65.00 | | 8.00 | | 77.37 | |  |  |
| APBB1IP | 3.67 | | 0.00 | | 0.27 | | 1.00 | | 65.00 | | 8.00 | | 77.37 | |  |  |
| ANKRD17 | 3.67 | | 0.00 | | 0.27 | | 1.00 | | 65.00 | | 8.00 | | 77.37 | |  |  |
| HAVCR2 | 3.67 | | 0.00 | | 0.27 | | 1.00 | | 65.00 | | 8.00 | | 77.37 | |  |  |
| KIF3A | 3.67 | | 0.00 | | 0.27 | | 1.00 | | 65.00 | | 8.00 | | 77.37 | |  |  |
| ATP7A | 3.67 | | 0.00 | | 0.27 | | 1.00 | | 65.00 | | 8.00 | | 77.37 | |  |  |
| IL6ST | 3.67 | | 0.00 | | 0.27 | | 1.00 | | 65.00 | | 8.00 | | 77.37 | |  |  |
| RASGRP1 | 3.67 | | 0.00 | | 0.27 | | 1.00 | | 65.00 | | 8.00 | | 77.37 | |  |  |
| CD79A | 3.67 | | 0.00 | | 0.27 | | 1.00 | | 65.00 | | 8.00 | | 77.37 | |  |  |
| ADGRE1 | 3.67 | | 0.00 | | 0.27 | | 1.00 | | 65.00 | | 8.00 | | 77.37 | |  |  |
| IGLV1-40 | 3.67 | | 0.00 | | 0.27 | | 1.00 | | 65.00 | | 8.00 | | 77.37 | |  |  |
| CD3G | 3.67 | | 0.00 | | 0.27 | | 1.00 | | 65.00 | | 8.00 | | 77.37 | |  |  |
| SELENOK | 3.67 | | 0.00 | | 0.27 | | 1.00 | | 65.00 | | 8.00 | | 77.37 | |  |  |
| RBM14 | 3.67 | | 0.00 | | 0.27 | | 1.00 | | 65.00 | | 8.00 | | 77.37 | |  |  |
| CD84 | 3.67 | | 0.00 | | 0.27 | | 1.00 | | 65.00 | | 8.00 | | 77.37 | |  |  |
| C12orf4 | 3.67 | | 0.00 | | 0.27 | | 1.00 | | 65.00 | | 8.00 | | 77.37 | |  |  |
| NCF1 | 3.67 | | 0.00 | | 0.27 | | 1.00 | | 65.00 | | 8.00 | | 77.37 | |  |  |
| RAB10 | 3.67 | | 0.00 | | 0.27 | | 1.00 | | 65.00 | | 8.00 | | 77.37 | |  |  |
| CD40 | 3.67 | | 0.00 | | 0.27 | | 1.00 | | 65.00 | | 8.00 | | 77.37 | |  |  |
| CD300LB | 3.67 | | 0.00 | | 0.27 | | 1.00 | | 65.00 | | 8.00 | | 77.37 | |  |  |
| LEO1 | 3.67 | | 0.00 | | 0.27 | | 1.00 | | 65.00 | | 8.00 | | 77.37 | |  |  |
| LAIR1 | 3.67 | | 0.00 | | 0.27 | | 1.00 | | 65.00 | | 8.00 | | 77.37 | |  |  |
| MYO1C | 3.67 | | 0.00 | | 0.27 | | 1.00 | | 65.00 | | 8.00 | | 77.37 | |  |  |
| HK3 | 3.67 | | 0.00 | | 0.27 | | 1.00 | | 65.00 | | 8.00 | | 77.37 | |  |  |
| LCP2 | 3.67 | | 0.00 | | 0.27 | | 1.00 | | 65.00 | | 8.00 | | 77.37 | |  |  |
| SCARB1 | 3.67 | | 0.00 | | 0.27 | | 1.00 | | 65.00 | | 8.00 | | 77.37 | |  |  |
| PLA2G2A | 4.40 | | 0.01 | | 0.23 | | 0.38 | | 63.00 | | 9.00 | | 30.86 | |  |  |
| HSPA6 | 4.40 | | 0.01 | | 0.23 | | 0.38 | | 63.00 | | 9.00 | | 30.86 | |  |  |
| P2RX1 | 4.13 | | 0.07 | | 0.24 | | 0.53 | | 62.00 | | 7.00 | | 33.90 | |  |  |
| IL2RA | 4.13 | | 0.07 | | 0.24 | | 0.53 | | 62.00 | | 7.00 | | 33.90 | |  |  |
| BCL2 | 4.13 | | 0.07 | | 0.24 | | 0.53 | | 62.00 | | 7.00 | | 33.90 | |  |  |
| Pol | 4.03 | | 0.25 | | 0.25 | | 0.50 | | 62.00 | | 7.00 | | 33.76 | |  |  |
| TNFRSF13B | 4.13 | | 0.07 | | 0.24 | | 0.53 | | 62.00 | | 7.00 | | 33.90 | |  |  |
| Itm2a | 4.37 | | 0.01 | | 0.23 | | 0.40 | | 55.00 | | 9.00 | | 29.24 | |  |  |
| Susd4 | 4.37 | | 0.01 | | 0.23 | | 0.40 | | 55.00 | | 9.00 | | 29.24 | |  |  |
| IL33 | 4.37 | | 0.01 | | 0.23 | | 0.40 | | 55.00 | | 9.00 | | 29.24 | |  |  |
| FRMPD3 | 4.37 | | 0.01 | | 0.23 | | 0.40 | | 55.00 | | 9.00 | | 29.24 | |  |  |
| SH2D1A | 4.37 | | 0.01 | | 0.23 | | 0.40 | | 55.00 | | 9.00 | | 29.24 | |  |  |
| GPR171 | 4.37 | | 0.01 | | 0.23 | | 0.40 | | 55.00 | | 9.00 | | 29.24 | |  |  |
| LY86 | 4.37 | | 0.01 | | 0.23 | | 0.40 | | 55.00 | | 9.00 | | 29.24 | |  |  |
| H2BC11 | 4.81 | | 0.06 | | 0.21 | | 0.44 | | 52.00 | | 8.00 | | 23.94 | |  |  |
| LIMK1 | 4.81 | | 0.06 | | 0.21 | | 0.44 | | 52.00 | | 8.00 | | 23.94 | |  |  |
| FCRL4 | 4.81 | | 0.06 | | 0.21 | | 0.44 | | 52.00 | | 8.00 | | 23.94 | |  |  |
| NOTCH1 | 4.81 | | 0.06 | | 0.21 | | 0.44 | | 52.00 | | 8.00 | | 23.94 | |  |  |
| ITGAX | 4.94 | | 0.04 | | 0.20 | | 0.47 | | 51.00 | | 9.00 | | 25.43 | |  |  |
| ANO6 | 4.94 | | 0.04 | | 0.20 | | 0.47 | | 51.00 | | 9.00 | | 25.43 | |  |  |
| MILR1 | 4.67 | | 0.03 | | 0.21 | | 0.51 | | 48.00 | | 8.00 | | 28.83 | |  |  |
| CARD11 | 3.37 | | 0.16 | | 0.30 | | 0.59 | | 48.00 | | 7.00 | | 37.83 | |  |  |
| NFATC1 | 4.67 | | 0.03 | | 0.21 | | 0.51 | | 48.00 | | 8.00 | | 28.83 | |  |  |
| CEACAM1 | 3.56 | | 0.04 | | 0.28 | | 0.74 | | 47.00 | | 7.00 | | 43.02 | |  |  |
| HMGB2 | 3.22 | | 0.00 | | 0.31 | | 1.00 | | 45.00 | | 6.00 | | 54.07 | |  |  |
| PLAC8 | 3.22 | | 0.00 | | 0.31 | | 1.00 | | 45.00 | | 6.00 | | 54.07 | |  |  |
| LY96 | 3.22 | | 0.00 | | 0.31 | | 1.00 | | 45.00 | | 6.00 | | 54.07 | |  |  |
| GRB2 | 3.22 | | 0.00 | | 0.31 | | 1.00 | | 45.00 | | 6.00 | | 54.07 | |  |  |
| TNFRSF21 | 3.22 | | 0.00 | | 0.31 | | 1.00 | | 45.00 | | 6.00 | | 54.07 | |  |  |
| BRAF | 3.22 | | 0.00 | | 0.31 | | 1.00 | | 45.00 | | 6.00 | | 54.07 | |  |  |
| RC3H2 | 3.22 | | 0.00 | | 0.31 | | 1.00 | | 45.00 | | 6.00 | | 54.07 | |  |  |
| SUMO1 | 3.22 | | 0.00 | | 0.31 | | 1.00 | | 45.00 | | 6.00 | | 54.07 | |  |  |
| VAV2 | 3.22 | | 0.00 | | 0.31 | | 1.00 | | 45.00 | | 6.00 | | 54.07 | |  |  |
| Zbtb7b | 3.22 | | 0.00 | | 0.31 | | 1.00 | | 45.00 | | 6.00 | | 54.07 | |  |  |
| CYRIB | 3.22 | | 0.00 | | 0.31 | | 1.00 | | 45.00 | | 6.00 | | 54.07 | |  |  |
| ERAP1 | 3.22 | | 0.00 | | 0.31 | | 1.00 | | 45.00 | | 6.00 | | 54.07 | |  |  |
| NSD2 | 3.22 | | 0.00 | | 0.31 | | 1.00 | | 45.00 | | 6.00 | | 54.07 | |  |  |
| ACE | 3.22 | | 0.00 | | 0.31 | | 1.00 | | 45.00 | | 6.00 | | 54.07 | |  |  |
| GNA11 | 3.22 | | 0.00 | | 0.31 | | 1.00 | | 45.00 | | 6.00 | | 54.07 | |  |  |
| PPP2R3C | 3.22 | | 0.00 | | 0.31 | | 1.00 | | 45.00 | | 6.00 | | 54.07 | |  |  |
| DSN1 | 3.22 | | 0.00 | | 0.31 | | 1.00 | | 45.00 | | 6.00 | | 54.07 | |  |  |
| HLA-A | 3.22 | | 0.00 | | 0.31 | | 1.00 | | 45.00 | | 6.00 | | 54.07 | |  |  |
| SERPINB9 | 3.22 | | 0.00 | | 0.31 | | 1.00 | | 45.00 | | 6.00 | | 54.07 | |  |  |
| ARSA | 3.22 | | 0.00 | | 0.31 | | 1.00 | | 45.00 | | 6.00 | | 54.07 | |  |  |
| GP2 | 3.22 | | 0.00 | | 0.31 | | 1.00 | | 45.00 | | 6.00 | | 54.07 | |  |  |
| KIF23 | 3.22 | | 0.00 | | 0.31 | | 1.00 | | 45.00 | | 6.00 | | 54.07 | |  |  |
| ZP2 | 3.22 | | 0.00 | | 0.31 | | 1.00 | | 45.00 | | 6.00 | | 54.07 | |  |  |
| MOSPD2 | 3.22 | | 0.00 | | 0.31 | | 1.00 | | 45.00 | | 6.00 | | 54.07 | |  |  |
| CBFB | 3.22 | | 0.00 | | 0.31 | | 1.00 | | 45.00 | | 6.00 | | 54.07 | |  |  |
| F2RL1 | 3.22 | | 0.00 | | 0.31 | | 1.00 | | 45.00 | | 6.00 | | 54.07 | |  |  |
| MEF2C | 3.22 | | 0.00 | | 0.31 | | 1.00 | | 45.00 | | 6.00 | | 54.07 | |  |  |
| JAK2 | 3.22 | | 0.00 | | 0.31 | | 1.00 | | 45.00 | | 6.00 | | 54.07 | |  |  |
| ARPC3 | 3.22 | | 0.00 | | 0.31 | | 1.00 | | 45.00 | | 6.00 | | 54.07 | |  |  |
| CDK13 | 3.22 | | 0.00 | | 0.31 | | 1.00 | | 45.00 | | 6.00 | | 54.07 | |  |  |
| ALCAM | 3.22 | | 0.00 | | 0.31 | | 1.00 | | 45.00 | | 6.00 | | 54.07 | |  |  |
| DCAF1 | 3.22 | | 0.00 | | 0.31 | | 1.00 | | 45.00 | | 6.00 | | 54.07 | |  |  |
| BCL6 | 3.22 | | 0.00 | | 0.31 | | 1.00 | | 45.00 | | 6.00 | | 54.07 | |  |  |
| CGAS | 3.22 | | 0.00 | | 0.31 | | 1.00 | | 45.00 | | 6.00 | | 54.07 | |  |  |
| RACGAP1 | 3.22 | | 0.00 | | 0.31 | | 1.00 | | 45.00 | | 6.00 | | 54.07 | |  |  |
| TRPM4 | 3.22 | | 0.00 | | 0.31 | | 1.00 | | 45.00 | | 6.00 | | 54.07 | |  |  |
| RBM15 | 3.22 | | 0.00 | | 0.31 | | 1.00 | | 45.00 | | 6.00 | | 54.07 | |  |  |
| TTR | 3.22 | | 0.00 | | 0.31 | | 1.00 | | 45.00 | | 6.00 | | 54.07 | |  |  |
| UBASH3B | 3.22 | | 0.00 | | 0.31 | | 1.00 | | 45.00 | | 6.00 | | 54.07 | |  |  |
| ICAM1 | 3.68 | | 0.00 | | 0.27 | | 1.00 | | 40.00 | | 7.00 | | 48.45 | |  |  |
| LGALS9 | 3.68 | | 0.00 | | 0.27 | | 1.00 | | 40.00 | | 7.00 | | 48.45 | |  |  |
| IHH | 3.68 | | 0.00 | | 0.27 | | 1.00 | | 40.00 | | 7.00 | | 48.45 | |  |  |
| GGH | 3.68 | | 0.00 | | 0.27 | | 1.00 | | 40.00 | | 7.00 | | 48.45 | |  |  |
| SFTPD | 3.68 | | 0.00 | | 0.27 | | 1.00 | | 40.00 | | 7.00 | | 48.45 | |  |  |
| Mapk3 | 3.68 | | 0.00 | | 0.27 | | 1.00 | | 40.00 | | 7.00 | | 48.45 | |  |  |
| DGAT1 | 3.68 | | 0.00 | | 0.27 | | 1.00 | | 40.00 | | 7.00 | | 48.45 | |  |  |
| TOM1 | 3.68 | | 0.00 | | 0.27 | | 1.00 | | 40.00 | | 7.00 | | 48.45 | |  |  |
| ZAP70 | 4.77 | | 0.00 | | 0.21 | | 1.00 | | 40.00 | | 8.00 | | 43.03 | |  |  |
| TRBV14 | 4.77 | | 0.00 | | 0.21 | | 1.00 | | 40.00 | | 8.00 | | 43.03 | |  |  |
| Jup | 3.68 | | 0.00 | | 0.27 | | 1.00 | | 40.00 | | 7.00 | | 48.45 | |  |  |
| TRB | 4.77 | | 0.00 | | 0.21 | | 1.00 | | 40.00 | | 8.00 | | 43.03 | |  |  |
| NBL1 | 4.77 | | 0.00 | | 0.21 | | 1.00 | | 40.00 | | 8.00 | | 43.03 | |  |  |
| PRKCH | 4.77 | | 0.00 | | 0.21 | | 1.00 | | 40.00 | | 8.00 | | 43.03 | |  |  |
| APOD | 4.77 | | 0.00 | | 0.21 | | 1.00 | | 40.00 | | 8.00 | | 43.03 | |  |  |
| TRDMT1 | 4.77 | | 0.00 | | 0.21 | | 1.00 | | 40.00 | | 8.00 | | 43.03 | |  |  |
| TICAM1 | 3.68 | | 0.00 | | 0.27 | | 1.00 | | 40.00 | | 7.00 | | 48.45 | |  |  |
| CD247 | 4.77 | | 0.00 | | 0.21 | | 1.00 | | 40.00 | | 8.00 | | 43.03 | |  |  |
| MAPKAPK3 | 3.68 | | 0.00 | | 0.27 | | 1.00 | | 40.00 | | 7.00 | | 48.45 | |  |  |
| BCAR1 | 3.68 | | 0.00 | | 0.27 | | 1.00 | | 40.00 | | 7.00 | | 48.45 | |  |  |
| TWSG1 | 4.77 | | 0.00 | | 0.21 | | 1.00 | | 40.00 | | 8.00 | | 43.03 | |  |  |
| PRDX6 | 3.68 | | 0.00 | | 0.27 | | 1.00 | | 40.00 | | 7.00 | | 48.45 | |  |  |
| NLRP6 | 3.68 | | 0.00 | | 0.27 | | 1.00 | | 40.00 | | 7.00 | | 48.45 | |  |  |
| FOSL2 | 3.68 | | 0.00 | | 0.27 | | 1.00 | | 40.00 | | 7.00 | | 48.45 | |  |  |
| TMEM106B | 4.77 | | 0.00 | | 0.21 | | 1.00 | | 40.00 | | 8.00 | | 43.03 | |  |  |
| Pol | 4.77 | | 0.00 | | 0.21 | | 1.00 | | 40.00 | | 8.00 | | 43.03 | |  |  |
| GATA3 | 4.77 | | 0.00 | | 0.21 | | 1.00 | | 40.00 | | 8.00 | | 43.03 | |  |  |
| STAT5B | 4.77 | | 0.00 | | 0.21 | | 1.00 | | 40.00 | | 8.00 | | 43.03 | |  |  |
| Dnm2 | 3.68 | | 0.00 | | 0.27 | | 1.00 | | 40.00 | | 7.00 | | 48.45 | |  |  |
| Src | 3.68 | | 0.00 | | 0.27 | | 1.00 | | 40.00 | | 7.00 | | 48.45 | |  |  |
| CCL20 | 4.77 | | 0.00 | | 0.21 | | 1.00 | | 40.00 | | 8.00 | | 43.03 | |  |  |
| ITGB2 | 4.77 | | 0.00 | | 0.21 | | 1.00 | | 40.00 | | 8.00 | | 43.03 | |  |  |
| INAVA | 3.68 | | 0.00 | | 0.27 | | 1.00 | | 40.00 | | 7.00 | | 48.45 | |  |  |
| POLQ | 3.68 | | 0.00 | | 0.27 | | 1.00 | | 40.00 | | 7.00 | | 48.45 | |  |  |
| FABP5 | 4.77 | | 0.00 | | 0.21 | | 1.00 | | 40.00 | | 8.00 | | 43.03 | |  |  |
| S100B | 4.77 | | 0.00 | | 0.21 | | 1.00 | | 40.00 | | 8.00 | | 43.03 | |  |  |
| RBPJ | 4.77 | | 0.00 | | 0.21 | | 1.00 | | 40.00 | | 8.00 | | 43.03 | |  |  |
| Gm2a | 4.77 | | 0.00 | | 0.21 | | 1.00 | | 40.00 | | 8.00 | | 43.03 | |  |  |
| TRB | 4.77 | | 0.00 | | 0.21 | | 1.00 | | 40.00 | | 8.00 | | 43.03 | |  |  |
| MAPKAPK2 | 4.77 | | 0.00 | | 0.21 | | 1.00 | | 40.00 | | 8.00 | | 43.03 | |  |  |
| MEIS2 | 4.77 | | 0.00 | | 0.21 | | 1.00 | | 40.00 | | 8.00 | | 43.03 | |  |  |
| C1R | 4.77 | | 0.00 | | 0.21 | | 1.00 | | 40.00 | | 8.00 | | 43.03 | |  |  |
| ADGRF5 | 4.77 | | 0.00 | | 0.21 | | 1.00 | | 40.00 | | 8.00 | | 43.03 | |  |  |
| N4BP2L2 | 4.77 | | 0.00 | | 0.21 | | 1.00 | | 40.00 | | 8.00 | | 43.03 | |  |  |
| PECAM1 | 4.77 | | 0.00 | | 0.21 | | 1.00 | | 40.00 | | 8.00 | | 43.03 | |  |  |
| FOS | 3.68 | | 0.00 | | 0.27 | | 1.00 | | 40.00 | | 7.00 | | 48.45 | |  |  |
| CD80 | 3.68 | | 0.00 | | 0.27 | | 1.00 | | 40.00 | | 7.00 | | 48.45 | |  |  |
| PPP3CA | 3.68 | | 0.00 | | 0.27 | | 1.00 | | 40.00 | | 7.00 | | 48.45 | |  |  |
| ITK | 3.68 | | 0.00 | | 0.27 | | 1.00 | | 40.00 | | 7.00 | | 48.45 | |  |  |
| SEMA7A | 3.68 | | 0.00 | | 0.27 | | 1.00 | | 40.00 | | 7.00 | | 48.45 | |  |  |
| NEU1 | 3.68 | | 0.00 | | 0.27 | | 1.00 | | 40.00 | | 7.00 | | 48.45 | |  |  |
| CANT1 | 3.68 | | 0.00 | | 0.27 | | 1.00 | | 40.00 | | 7.00 | | 48.45 | |  |  |
| TYRO3 | 3.68 | | 0.00 | | 0.27 | | 1.00 | | 40.00 | | 7.00 | | 48.45 | |  |  |
| PFKL | 3.68 | | 0.00 | | 0.27 | | 1.00 | | 40.00 | | 7.00 | | 48.45 | |  |  |
| MARCHF7 | 4.77 | | 0.00 | | 0.21 | | 1.00 | | 40.00 | | 8.00 | | 43.03 | |  |  |
| CACNB3 | 4.77 | | 0.00 | | 0.21 | | 1.00 | | 40.00 | | 8.00 | | 43.03 | |  |  |
| SH2B2 | 3.68 | | 0.00 | | 0.27 | | 1.00 | | 40.00 | | 7.00 | | 48.45 | |  |  |
| BATF | 4.77 | | 0.00 | | 0.21 | | 1.00 | | 40.00 | | 8.00 | | 43.03 | |  |  |
| ADCY7 | 4.77 | | 0.00 | | 0.21 | | 1.00 | | 40.00 | | 8.00 | | 43.03 | |  |  |
| SERPING1 | 4.77 | | 0.00 | | 0.21 | | 1.00 | | 40.00 | | 8.00 | | 43.03 | |  |  |
| SKAP1 | 4.77 | | 0.00 | | 0.21 | | 1.00 | | 40.00 | | 8.00 | | 43.03 | |  |  |
| TRIM15 | 3.68 | | 0.00 | | 0.27 | | 1.00 | | 40.00 | | 7.00 | | 48.45 | |  |  |
| C7 | 4.77 | | 0.00 | | 0.21 | | 1.00 | | 40.00 | | 8.00 | | 43.03 | |  |  |
| PPARG | 3.68 | | 0.00 | | 0.27 | | 1.00 | | 40.00 | | 7.00 | | 48.45 | |  |  |
| MUC1 | 3.68 | | 0.00 | | 0.27 | | 1.00 | | 40.00 | | 7.00 | | 48.45 | |  |  |
| DTX3L | 4.77 | | 0.00 | | 0.21 | | 1.00 | | 40.00 | | 8.00 | | 43.03 | |  |  |
| PLD2 | 3.68 | | 0.00 | | 0.27 | | 1.00 | | 40.00 | | 7.00 | | 48.45 | |  |  |
| Ubash3a | 4.77 | | 0.00 | | 0.21 | | 1.00 | | 40.00 | | 8.00 | | 43.03 | |  |  |
| SOCS3 | 4.77 | | 0.00 | | 0.21 | | 1.00 | | 40.00 | | 8.00 | | 43.03 | |  |  |
| TRA | 4.77 | | 0.00 | | 0.21 | | 1.00 | | 40.00 | | 8.00 | | 43.03 | |  |  |
| Oscar | 3.38 | | 0.49 | | 0.30 | | 0.39 | | 38.00 | | 5.00 | | 16.63 | |  |  |
| Pol | 4.19 | | 0.03 | | 0.24 | | 0.65 | | 38.00 | | 7.00 | | 29.21 | |  |  |
| FOXF1 | 4.19 | | 0.03 | | 0.24 | | 0.65 | | 38.00 | | 7.00 | | 29.21 | |  |  |
| PDCD1LG2 | 4.19 | | 0.03 | | 0.24 | | 0.65 | | 38.00 | | 7.00 | | 29.21 | |  |  |
| Sec14l1 | 3.49 | | 0.00 | | 0.29 | | 1.00 | | 35.00 | | 7.00 | | 45.37 | |  |  |
| TRIM44 | 5.01 | | 0.00 | | 0.20 | | 1.00 | | 35.00 | | 8.00 | | 38.09 | |  |  |
| CAV1 | 5.01 | | 0.00 | | 0.20 | | 1.00 | | 35.00 | | 8.00 | | 38.09 | |  |  |
| HEBP2 | 3.49 | | 0.00 | | 0.29 | | 1.00 | | 35.00 | | 7.00 | | 45.37 | |  |  |
| IL1B | 3.49 | | 0.00 | | 0.29 | | 1.00 | | 35.00 | | 7.00 | | 45.37 | |  |  |
| SIGLEC14 | 5.01 | | 0.00 | | 0.20 | | 1.00 | | 35.00 | | 8.00 | | 38.09 | |  |  |
| EIF2AK4 | 5.01 | | 0.00 | | 0.20 | | 1.00 | | 35.00 | | 8.00 | | 38.09 | |  |  |
| Patr-A | 5.01 | | 0.00 | | 0.20 | | 1.00 | | 35.00 | | 8.00 | | 38.09 | |  |  |
| SEC24B | 5.01 | | 0.00 | | 0.20 | | 1.00 | | 35.00 | | 8.00 | | 38.09 | |  |  |
| RUNX3 | 3.49 | | 0.00 | | 0.29 | | 1.00 | | 35.00 | | 7.00 | | 45.37 | |  |  |
| LPXN | 4.14 | | 0.00 | | 0.24 | | 1.00 | | 35.00 | | 8.00 | | 43.77 | |  |  |
| PLCB1 | 5.01 | | 0.00 | | 0.20 | | 1.00 | | 35.00 | | 8.00 | | 38.09 | |  |  |
| NIT2 | 5.01 | | 0.00 | | 0.20 | | 1.00 | | 35.00 | | 8.00 | | 38.09 | |  |  |
| CBL | 3.49 | | 0.00 | | 0.29 | | 1.00 | | 35.00 | | 7.00 | | 45.37 | |  |  |
| ADAM15 | 3.49 | | 0.00 | | 0.29 | | 1.00 | | 35.00 | | 7.00 | | 45.37 | |  |  |
| A2M | 5.01 | | 0.00 | | 0.20 | | 1.00 | | 35.00 | | 8.00 | | 38.09 | |  |  |
| -- | 4.14 | | 0.00 | | 0.24 | | 1.00 | | 35.00 | | 8.00 | | 43.77 | |  |  |
| Rab3d | 4.14 | | 0.00 | | 0.24 | | 1.00 | | 35.00 | | 8.00 | | 43.77 | |  |  |
| GLMN | 3.49 | | 0.00 | | 0.29 | | 1.00 | | 35.00 | | 7.00 | | 45.37 | |  |  |
| MED1 | 3.49 | | 0.00 | | 0.29 | | 1.00 | | 35.00 | | 7.00 | | 45.37 | |  |  |
| FER | 3.49 | | 0.00 | | 0.29 | | 1.00 | | 35.00 | | 7.00 | | 45.37 | |  |  |
| PML | 5.01 | | 0.00 | | 0.20 | | 1.00 | | 35.00 | | 8.00 | | 38.09 | |  |  |
| CRK | 5.01 | | 0.00 | | 0.20 | | 1.00 | | 35.00 | | 8.00 | | 38.09 | |  |  |
| TMEM131L | 4.14 | | 0.00 | | 0.24 | | 1.00 | | 35.00 | | 8.00 | | 43.77 | |  |  |
| IFNLR1 | 4.14 | | 0.00 | | 0.24 | | 1.00 | | 35.00 | | 8.00 | | 43.77 | |  |  |
| DDX58 | 5.01 | | 0.00 | | 0.20 | | 1.00 | | 35.00 | | 8.00 | | 38.09 | |  |  |
| FGL2 | 5.01 | | 0.00 | | 0.20 | | 1.00 | | 35.00 | | 8.00 | | 38.09 | |  |  |
| DDT | 4.14 | | 0.00 | | 0.24 | | 1.00 | | 35.00 | | 8.00 | | 43.77 | |  |  |
| ARRB2 | 5.01 | | 0.00 | | 0.20 | | 1.00 | | 35.00 | | 8.00 | | 38.09 | |  |  |
| ADORA2B | 4.14 | | 0.00 | | 0.24 | | 1.00 | | 35.00 | | 8.00 | | 43.77 | |  |  |
| LAMP1 | 4.14 | | 0.00 | | 0.24 | | 1.00 | | 35.00 | | 8.00 | | 43.77 | |  |  |
| GPR18 | 3.49 | | 0.00 | | 0.29 | | 1.00 | | 35.00 | | 7.00 | | 45.37 | |  |  |
| IGLV1-40 | 4.14 | | 0.00 | | 0.24 | | 1.00 | | 35.00 | | 8.00 | | 43.77 | |  |  |
| KIF2C | 4.14 | | 0.00 | | 0.24 | | 1.00 | | 35.00 | | 8.00 | | 43.77 | |  |  |
| SYK | 3.49 | | 0.00 | | 0.29 | | 1.00 | | 35.00 | | 7.00 | | 45.37 | |  |  |
| MFNG | 3.49 | | 0.00 | | 0.29 | | 1.00 | | 35.00 | | 7.00 | | 45.37 | |  |  |
| Prkcb | 3.49 | | 0.00 | | 0.29 | | 1.00 | | 35.00 | | 7.00 | | 45.37 | |  |  |
| XRCC5 | 3.49 | | 0.00 | | 0.29 | | 1.00 | | 35.00 | | 7.00 | | 45.37 | |  |  |
| AP1M2 | 4.14 | | 0.00 | | 0.24 | | 1.00 | | 35.00 | | 8.00 | | 43.77 | |  |  |
| AP1S2 | 4.14 | | 0.00 | | 0.24 | | 1.00 | | 35.00 | | 8.00 | | 43.77 | |  |  |
| VAMP8 | 3.49 | | 0.00 | | 0.29 | | 1.00 | | 35.00 | | 7.00 | | 45.37 | |  |  |
| DOCK2 | 3.49 | | 0.00 | | 0.29 | | 1.00 | | 35.00 | | 7.00 | | 45.37 | |  |  |
| SOCS5 | 5.01 | | 0.00 | | 0.20 | | 1.00 | | 35.00 | | 8.00 | | 38.09 | |  |  |
| CFB | 5.01 | | 0.00 | | 0.20 | | 1.00 | | 35.00 | | 8.00 | | 38.09 | |  |  |
| TNFRSF1B | 5.01 | | 0.00 | | 0.20 | | 1.00 | | 35.00 | | 8.00 | | 38.09 | |  |  |
| YES1 | 5.01 | | 0.00 | | 0.20 | | 1.00 | | 35.00 | | 8.00 | | 38.09 | |  |  |
| RAB17 | 4.14 | | 0.00 | | 0.24 | | 1.00 | | 35.00 | | 8.00 | | 43.77 | |  |  |
| FAS | 3.49 | | 0.00 | | 0.29 | | 1.00 | | 35.00 | | 7.00 | | 45.37 | |  |  |
| FCGR2 | 5.01 | | 0.00 | | 0.20 | | 1.00 | | 35.00 | | 8.00 | | 38.09 | |  |  |
| CD63 | 4.14 | | 0.00 | | 0.24 | | 1.00 | | 35.00 | | 8.00 | | 43.77 | |  |  |
| Cda | 4.14 | | 0.00 | | 0.24 | | 1.00 | | 35.00 | | 8.00 | | 43.77 | |  |  |
| GSTP1 | 4.14 | | 0.00 | | 0.24 | | 1.00 | | 35.00 | | 8.00 | | 43.77 | |  |  |
| CDH17 | 4.14 | | 0.00 | | 0.24 | | 1.00 | | 35.00 | | 8.00 | | 43.77 | |  |  |
| HMOX2 | 4.14 | | 0.00 | | 0.24 | | 1.00 | | 35.00 | | 8.00 | | 43.77 | |  |  |
| MS4A1 | 3.49 | | 0.00 | | 0.29 | | 1.00 | | 35.00 | | 7.00 | | 45.37 | |  |  |
| Ccr2 | 4.14 | | 0.00 | | 0.24 | | 1.00 | | 35.00 | | 8.00 | | 43.77 | |  |  |
| MVP | 4.14 | | 0.00 | | 0.24 | | 1.00 | | 35.00 | | 8.00 | | 43.77 | |  |  |
| LGALS3 | 3.49 | | 0.00 | | 0.29 | | 1.00 | | 35.00 | | 7.00 | | 45.37 | |  |  |
| CCN3 | 5.01 | | 0.00 | | 0.20 | | 1.00 | | 35.00 | | 8.00 | | 38.09 | |  |  |
| ALAD | 4.14 | | 0.00 | | 0.24 | | 1.00 | | 35.00 | | 8.00 | | 43.77 | |  |  |
| DNM2 | 4.14 | | 0.00 | | 0.24 | | 1.00 | | 35.00 | | 8.00 | | 43.77 | |  |  |
| B4GALT1 | 3.49 | | 0.00 | | 0.29 | | 1.00 | | 35.00 | | 7.00 | | 45.37 | |  |  |
| HFE | 4.14 | | 0.00 | | 0.24 | | 1.00 | | 35.00 | | 8.00 | | 43.77 | |  |  |
| RIPK2 | 4.14 | | 0.00 | | 0.24 | | 1.00 | | 35.00 | | 8.00 | | 43.77 | |  |  |
| Tmem178a | 3.49 | | 0.00 | | 0.29 | | 1.00 | | 35.00 | | 7.00 | | 45.37 | |  |  |
| TRIL | 3.49 | | 0.00 | | 0.29 | | 1.00 | | 35.00 | | 7.00 | | 45.37 | |  |  |
| AGPAT2 | 4.14 | | 0.00 | | 0.24 | | 1.00 | | 35.00 | | 8.00 | | 43.77 | |  |  |
| FCRL3 | 5.01 | | 0.00 | | 0.20 | | 1.00 | | 35.00 | | 8.00 | | 38.09 | |  |  |
| CRISPLD2 | 5.01 | | 0.00 | | 0.20 | | 1.00 | | 35.00 | | 8.00 | | 38.09 | |  |  |
| KLHL6 | 3.49 | | 0.00 | | 0.29 | | 1.00 | | 35.00 | | 7.00 | | 45.37 | |  |  |
| ATG5 | 5.01 | | 0.00 | | 0.20 | | 1.00 | | 35.00 | | 8.00 | | 38.09 | |  |  |
| SYNGR1 | 5.01 | | 0.00 | | 0.20 | | 1.00 | | 35.00 | | 8.00 | | 38.09 | |  |  |
| TNFRSF11A | 5.01 | | 0.00 | | 0.20 | | 1.00 | | 35.00 | | 8.00 | | 38.09 | |  |  |
| Fcer1g | 5.01 | | 0.00 | | 0.20 | | 1.00 | | 35.00 | | 8.00 | | 38.09 | |  |  |
| NLRC5 | 5.01 | | 0.00 | | 0.20 | | 1.00 | | 35.00 | | 8.00 | | 38.09 | |  |  |
| DNAJC3 | 5.01 | | 0.00 | | 0.20 | | 1.00 | | 35.00 | | 8.00 | | 38.09 | |  |  |
| MYC | 5.01 | | 0.00 | | 0.20 | | 1.00 | | 35.00 | | 8.00 | | 38.09 | |  |  |
| CD46 | 5.01 | | 0.00 | | 0.20 | | 1.00 | | 35.00 | | 8.00 | | 38.09 | |  |  |
| ADAR | 5.01 | | 0.00 | | 0.20 | | 1.00 | | 35.00 | | 8.00 | | 38.09 | |  |  |
| CREG1 | 5.01 | | 0.00 | | 0.20 | | 1.00 | | 35.00 | | 8.00 | | 38.09 | |  |  |
| SAMHD1 | 3.49 | | 0.00 | | 0.29 | | 1.00 | | 35.00 | | 7.00 | | 45.37 | |  |  |
| IKZF1 | 3.49 | | 0.00 | | 0.29 | | 1.00 | | 35.00 | | 7.00 | | 45.37 | |  |  |
| PIK3AP1 | 3.49 | | 0.00 | | 0.29 | | 1.00 | | 35.00 | | 7.00 | | 45.37 | |  |  |
| TCTA | 4.14 | | 0.00 | | 0.24 | | 1.00 | | 35.00 | | 8.00 | | 43.77 | |  |  |
| AOC1 | 3.49 | | 0.00 | | 0.29 | | 1.00 | | 35.00 | | 7.00 | | 45.37 | |  |  |
| PAK1 | 4.14 | | 0.00 | | 0.24 | | 1.00 | | 35.00 | | 8.00 | | 43.77 | |  |  |
| TNIP3 | 4.14 | | 0.00 | | 0.24 | | 1.00 | | 35.00 | | 8.00 | | 43.77 | |  |  |
| ZNF271 | 5.89 | | 0.00 | | 0.17 | | 1.00 | | 32.00 | | 10.00 | | 33.19 | |  |  |
| CTSS | 5.89 | | 0.00 | | 0.17 | | 1.00 | | 32.00 | | 10.00 | | 33.19 | |  |  |
| RPL30 | 3.75 | | 0.00 | | 0.27 | | 1.00 | | 32.00 | | 8.00 | | 40.69 | |  |  |
| RNASE6 | 3.75 | | 0.00 | | 0.27 | | 1.00 | | 32.00 | | 8.00 | | 40.69 | |  |  |
| JAK3 | 3.75 | | 0.00 | | 0.27 | | 1.00 | | 32.00 | | 8.00 | | 40.69 | |  |  |
| LAT2 | 3.75 | | 0.00 | | 0.27 | | 1.00 | | 32.00 | | 8.00 | | 40.69 | |  |  |
| RPS27A | 3.75 | | 0.00 | | 0.27 | | 1.00 | | 32.00 | | 8.00 | | 40.69 | |  |  |
| TRAT1 | 3.75 | | 0.00 | | 0.27 | | 1.00 | | 32.00 | | 8.00 | | 40.69 | |  |  |
| RNF168 | 3.75 | | 0.00 | | 0.27 | | 1.00 | | 32.00 | | 8.00 | | 40.69 | |  |  |
| CANX | 5.89 | | 0.00 | | 0.17 | | 1.00 | | 32.00 | | 10.00 | | 33.19 | |  |  |
| ITGAX | 5.89 | | 0.00 | | 0.17 | | 1.00 | | 32.00 | | 10.00 | | 33.19 | |  |  |
| IRAK4 | 5.89 | | 0.00 | | 0.17 | | 1.00 | | 32.00 | | 10.00 | | 33.19 | |  |  |
| PJA2 | 5.89 | | 0.00 | | 0.17 | | 1.00 | | 32.00 | | 10.00 | | 33.19 | |  |  |
| IQGAP1 | 5.89 | | 0.00 | | 0.17 | | 1.00 | | 32.00 | | 10.00 | | 33.19 | |  |  |
| ATP6V1D | 5.89 | | 0.00 | | 0.17 | | 1.00 | | 32.00 | | 10.00 | | 33.19 | |  |  |
| Sin3a | 5.89 | | 0.00 | | 0.17 | | 1.00 | | 32.00 | | 10.00 | | 33.19 | |  |  |
| FAF2 | 5.89 | | 0.00 | | 0.17 | | 1.00 | | 32.00 | | 10.00 | | 33.19 | |  |  |
| IL18RAP | 4.18 | | 0.11 | | 0.24 | | 0.39 | | 32.00 | | 8.00 | | 18.13 | |  |  |
| CXorf21 | 5.89 | | 0.00 | | 0.17 | | 1.00 | | 32.00 | | 10.00 | | 33.19 | |  |  |
| TREM2 | 5.89 | | 0.00 | | 0.17 | | 1.00 | | 32.00 | | 10.00 | | 33.19 | |  |  |
| LMO2 | 3.75 | | 0.00 | | 0.27 | | 1.00 | | 32.00 | | 8.00 | | 40.69 | |  |  |
| NBN | 3.75 | | 0.00 | | 0.27 | | 1.00 | | 32.00 | | 8.00 | | 40.69 | |  |  |
| STAP1 | 3.75 | | 0.00 | | 0.27 | | 1.00 | | 32.00 | | 8.00 | | 40.69 | |  |  |
| Tcf3 | 3.75 | | 0.00 | | 0.27 | | 1.00 | | 32.00 | | 8.00 | | 40.69 | |  |  |
| PLA2G3 | 3.75 | | 0.00 | | 0.27 | | 1.00 | | 32.00 | | 8.00 | | 40.69 | |  |  |
| IGLV1-40 | 5.89 | | 0.00 | | 0.17 | | 1.00 | | 32.00 | | 10.00 | | 33.19 | |  |  |
| ITGAV | 5.89 | | 0.00 | | 0.17 | | 1.00 | | 32.00 | | 10.00 | | 33.19 | |  |  |
| ADAM10 | 5.89 | | 0.00 | | 0.17 | | 1.00 | | 32.00 | | 10.00 | | 33.19 | |  |  |
| Klre1 | 3.75 | | 0.00 | | 0.27 | | 1.00 | | 32.00 | | 8.00 | | 40.69 | |  |  |
| CTSZ | 3.75 | | 0.00 | | 0.27 | | 1.00 | | 32.00 | | 8.00 | | 40.69 | |  |  |
| IL1R1 | 3.75 | | 0.00 | | 0.27 | | 1.00 | | 32.00 | | 8.00 | | 40.69 | |  |  |
| LIG4 | 3.75 | | 0.00 | | 0.27 | | 1.00 | | 32.00 | | 8.00 | | 40.69 | |  |  |
| MUC5AC | 3.75 | | 0.00 | | 0.27 | | 1.00 | | 32.00 | | 8.00 | | 40.69 | |  |  |
| LTA4H | 5.89 | | 0.00 | | 0.17 | | 1.00 | | 32.00 | | 10.00 | | 33.19 | |  |  |
| XRCC4 | 5.89 | | 0.00 | | 0.17 | | 1.00 | | 32.00 | | 10.00 | | 33.19 | |  |  |
| LRRC17 | 3.75 | | 0.00 | | 0.27 | | 1.00 | | 32.00 | | 8.00 | | 40.69 | |  |  |
| PSMA5 | 5.89 | | 0.00 | | 0.17 | | 1.00 | | 32.00 | | 10.00 | | 33.19 | |  |  |
| PTPN22 | 3.75 | | 0.00 | | 0.27 | | 1.00 | | 32.00 | | 8.00 | | 40.69 | |  |  |
| PRKDC | 5.89 | | 0.00 | | 0.17 | | 1.00 | | 32.00 | | 10.00 | | 33.19 | |  |  |
| GLI3 | 3.75 | | 0.00 | | 0.27 | | 1.00 | | 32.00 | | 8.00 | | 40.69 | |  |  |
| TLR7 | 5.89 | | 0.00 | | 0.17 | | 1.00 | | 32.00 | | 10.00 | | 33.19 | |  |  |
| CASP3 | 5.89 | | 0.00 | | 0.17 | | 1.00 | | 32.00 | | 10.00 | | 33.19 | |  |  |
| ITCH | 5.89 | | 0.00 | | 0.17 | | 1.00 | | 32.00 | | 10.00 | | 33.19 | |  |  |
| USP9X | 5.89 | | 0.00 | | 0.17 | | 1.00 | | 32.00 | | 10.00 | | 33.19 | |  |  |
| TFF2 | 5.89 | | 0.00 | | 0.17 | | 1.00 | | 32.00 | | 10.00 | | 33.19 | |  |  |
| PARP9 | 5.89 | | 0.00 | | 0.17 | | 1.00 | | 32.00 | | 10.00 | | 33.19 | |  |  |
| IGHG2 | 3.75 | | 0.00 | | 0.27 | | 1.00 | | 32.00 | | 8.00 | | 40.69 | |  |  |
| SAR1B | 3.75 | | 0.00 | | 0.27 | | 1.00 | | 32.00 | | 8.00 | | 40.69 | |  |  |
| PDE4B | 3.75 | | 0.00 | | 0.27 | | 1.00 | | 32.00 | | 8.00 | | 40.69 | |  |  |
| TAB3 | 5.89 | | 0.00 | | 0.17 | | 1.00 | | 32.00 | | 10.00 | | 33.19 | |  |  |
| UBR3 | 5.89 | | 0.00 | | 0.17 | | 1.00 | | 32.00 | | 10.00 | | 33.19 | |  |  |
| PTPRB | 3.75 | | 0.00 | | 0.27 | | 1.00 | | 32.00 | | 8.00 | | 40.69 | |  |  |
| CD28 | 3.75 | | 0.00 | | 0.27 | | 1.00 | | 32.00 | | 8.00 | | 40.69 | |  |  |
| CARTPT | 5.89 | | 0.00 | | 0.17 | | 1.00 | | 32.00 | | 10.00 | | 33.19 | |  |  |
| CDK6 | 3.75 | | 0.00 | | 0.27 | | 1.00 | | 32.00 | | 8.00 | | 40.69 | |  |  |
| SLAMF6 | 5.89 | | 0.00 | | 0.17 | | 1.00 | | 32.00 | | 10.00 | | 33.19 | |  |  |
| COPB1 | 5.89 | | 0.00 | | 0.17 | | 1.00 | | 32.00 | | 10.00 | | 33.19 | |  |  |
| IFIT1 | 3.75 | | 0.00 | | 0.27 | | 1.00 | | 32.00 | | 8.00 | | 40.69 | |  |  |
| CD274 | 5.89 | | 0.00 | | 0.17 | | 1.00 | | 32.00 | | 10.00 | | 33.19 | |  |  |
| NPY | 4.11 | | 0.01 | | 0.24 | | 0.47 | | 31.00 | | 8.00 | | 17.52 | |  |  |
| NCR1 | 4.85 | | 0.01 | | 0.21 | | 0.52 | | 31.00 | | 8.00 | | 19.48 | |  |  |
| APOA2 | 4.85 | | 0.01 | | 0.21 | | 0.52 | | 31.00 | | 8.00 | | 19.48 | |  |  |
| CCL8 | 4.85 | | 0.01 | | 0.21 | | 0.52 | | 31.00 | | 8.00 | | 19.48 | |  |  |
| FOXP3 | 4.11 | | 0.01 | | 0.24 | | 0.47 | | 31.00 | | 8.00 | | 17.52 | |  |  |
| GPER1 | 4.85 | | 0.01 | | 0.21 | | 0.52 | | 31.00 | | 8.00 | | 19.48 | |  |  |
| SNAP25 | 4.85 | | 0.01 | | 0.21 | | 0.52 | | 31.00 | | 8.00 | | 19.48 | |  |  |
| DEFB103A | 4.11 | | 0.01 | | 0.24 | | 0.47 | | 31.00 | | 8.00 | | 17.52 | |  |  |
| SH2D1B | 4.11 | | 0.01 | | 0.24 | | 0.47 | | 31.00 | | 8.00 | | 17.52 | |  |  |
| C1S | 5.38 | | 0.00 | | 0.19 | | 1.00 | | 30.00 | | 9.00 | | 35.50 | |  |  |
| PRAM1 | 5.38 | | 0.00 | | 0.19 | | 1.00 | | 30.00 | | 9.00 | | 35.50 | |  |  |
| TMEM176A | 5.38 | | 0.00 | | 0.19 | | 1.00 | | 30.00 | | 9.00 | | 35.50 | |  |  |
| SUPT5H | 5.38 | | 0.00 | | 0.19 | | 1.00 | | 30.00 | | 9.00 | | 35.50 | |  |  |
| WASF2 | 5.38 | | 0.00 | | 0.19 | | 1.00 | | 30.00 | | 9.00 | | 35.50 | |  |  |
| GPR183 | 5.38 | | 0.00 | | 0.19 | | 1.00 | | 30.00 | | 9.00 | | 35.50 | |  |  |
| BPI | 5.38 | | 0.00 | | 0.19 | | 1.00 | | 30.00 | | 9.00 | | 35.50 | |  |  |
| Ccl19 | 5.38 | | 0.00 | | 0.19 | | 1.00 | | 30.00 | | 9.00 | | 35.50 | |  |  |
| TRB | 5.38 | | 0.00 | | 0.19 | | 1.00 | | 30.00 | | 9.00 | | 35.50 | |  |  |
| CD8A | 5.38 | | 0.00 | | 0.19 | | 1.00 | | 30.00 | | 9.00 | | 35.50 | |  |  |
| TNFAIP8L2 | 5.38 | | 0.00 | | 0.19 | | 1.00 | | 30.00 | | 9.00 | | 35.50 | |  |  |
| MXRA8 | 5.38 | | 0.00 | | 0.19 | | 1.00 | | 30.00 | | 9.00 | | 35.50 | |  |  |
| CD81 | 5.38 | | 0.00 | | 0.19 | | 1.00 | | 30.00 | | 9.00 | | 35.50 | |  |  |
| LDB1 | 5.38 | | 0.00 | | 0.19 | | 1.00 | | 30.00 | | 9.00 | | 35.50 | |  |  |
| ZBP1 | 5.38 | | 0.00 | | 0.19 | | 1.00 | | 30.00 | | 9.00 | | 35.50 | |  |  |
| HLA-DQB1 | 5.38 | | 0.00 | | 0.19 | | 1.00 | | 30.00 | | 9.00 | | 35.50 | |  |  |
| C4A | 5.38 | | 0.00 | | 0.19 | | 1.00 | | 30.00 | | 9.00 | | 35.50 | |  |  |
| CD4 | 5.38 | | 0.00 | | 0.19 | | 1.00 | | 30.00 | | 9.00 | | 35.50 | |  |  |
| PIK3R2 | 5.38 | | 0.00 | | 0.19 | | 1.00 | | 30.00 | | 9.00 | | 35.50 | |  |  |
| CCR7 | 5.38 | | 0.00 | | 0.19 | | 1.00 | | 30.00 | | 9.00 | | 35.50 | |  |  |
| THY1 | 5.38 | | 0.00 | | 0.19 | | 1.00 | | 30.00 | | 9.00 | | 35.50 | |  |  |
| CX3CR1 | 5.38 | | 0.00 | | 0.19 | | 1.00 | | 30.00 | | 9.00 | | 35.50 | |  |  |
| DHX30 | 5.38 | | 0.00 | | 0.19 | | 1.00 | | 30.00 | | 9.00 | | 35.50 | |  |  |
| IL6R | 5.38 | | 0.00 | | 0.19 | | 1.00 | | 30.00 | | 9.00 | | 35.50 | |  |  |
| IRAK3 | 5.38 | | 0.00 | | 0.19 | | 1.00 | | 30.00 | | 9.00 | | 35.50 | |  |  |
| Dusp22 | 5.38 | | 0.00 | | 0.19 | | 1.00 | | 30.00 | | 9.00 | | 35.50 | |  |  |
| ANKHD1 | 4.98 | | 0.00 | | 0.20 | | 1.00 | | 29.00 | | 8.00 | | 34.48 | |  |  |
| MARCO | 4.98 | | 0.00 | | 0.20 | | 1.00 | | 29.00 | | 8.00 | | 34.48 | |  |  |
| PPIE | 4.98 | | 0.00 | | 0.20 | | 1.00 | | 29.00 | | 8.00 | | 34.48 | |  |  |
| C1qc | 4.98 | | 0.00 | | 0.20 | | 1.00 | | 29.00 | | 8.00 | | 34.48 | |  |  |
| USP46 | 4.98 | | 0.00 | | 0.20 | | 1.00 | | 29.00 | | 8.00 | | 34.48 | |  |  |
| FOXP1 | 4.98 | | 0.00 | | 0.20 | | 1.00 | | 29.00 | | 8.00 | | 34.48 | |  |  |
| AP3D1 | 4.98 | | 0.00 | | 0.20 | | 1.00 | | 29.00 | | 8.00 | | 34.48 | |  |  |
| IKBKB | 4.98 | | 0.00 | | 0.20 | | 1.00 | | 29.00 | | 8.00 | | 34.48 | |  |  |
| RNF125 | 4.98 | | 0.00 | | 0.20 | | 1.00 | | 29.00 | | 8.00 | | 34.48 | |  |  |
| IGF2R | 4.98 | | 0.00 | | 0.20 | | 1.00 | | 29.00 | | 8.00 | | 34.48 | |  |  |
| CD14 | 4.98 | | 0.00 | | 0.20 | | 1.00 | | 29.00 | | 8.00 | | 34.48 | |  |  |
| MMP2 | 4.98 | | 0.00 | | 0.20 | | 1.00 | | 29.00 | | 8.00 | | 34.48 | |  |  |
| Axl | 4.98 | | 0.00 | | 0.20 | | 1.00 | | 29.00 | | 8.00 | | 34.48 | |  |  |
| FCGR3 | 4.98 | | 0.00 | | 0.20 | | 1.00 | | 29.00 | | 8.00 | | 34.48 | |  |  |
| GATA2 | 4.98 | | 0.00 | | 0.20 | | 1.00 | | 29.00 | | 8.00 | | 34.48 | |  |  |
| IRF7 | 4.98 | | 0.00 | | 0.20 | | 1.00 | | 29.00 | | 8.00 | | 34.48 | |  |  |
| RAB24 | 4.98 | | 0.00 | | 0.20 | | 1.00 | | 29.00 | | 8.00 | | 34.48 | |  |  |
| TMEM176B | 4.98 | | 0.00 | | 0.20 | | 1.00 | | 29.00 | | 8.00 | | 34.48 | |  |  |
| CD33 | 4.98 | | 0.00 | | 0.20 | | 1.00 | | 29.00 | | 8.00 | | 34.48 | |  |  |
| ITGAM | 4.98 | | 0.00 | | 0.20 | | 1.00 | | 29.00 | | 8.00 | | 34.48 | |  |  |
| PRKCE | 4.98 | | 0.00 | | 0.20 | | 1.00 | | 29.00 | | 8.00 | | 34.48 | |  |  |
| HLA-DQA2 | 4.98 | | 0.00 | | 0.20 | | 1.00 | | 29.00 | | 8.00 | | 34.48 | |  |  |
| CCR6 | 4.98 | | 0.00 | | 0.20 | | 1.00 | | 29.00 | | 8.00 | | 34.48 | |  |  |
| ATP8A1 | 5.71 | | 0.00 | | 0.18 | | 1.00 | | 28.00 | | 9.00 | | 31.43 | |  |  |
| USP12 | 5.71 | | 0.00 | | 0.18 | | 1.00 | | 28.00 | | 9.00 | | 31.43 | |  |  |
| PGLYRP1 | 4.18 | | 0.02 | | 0.24 | | 0.45 | | 28.00 | | 8.00 | | 16.57 | |  |  |
| ALDOC | 5.71 | | 0.00 | | 0.18 | | 1.00 | | 28.00 | | 9.00 | | 31.43 | |  |  |
| Cfi | 5.71 | | 0.00 | | 0.18 | | 1.00 | | 28.00 | | 9.00 | | 31.43 | |  |  |
| IMPDH1 | 5.71 | | 0.00 | | 0.18 | | 1.00 | | 28.00 | | 9.00 | | 31.43 | |  |  |
| PYGL | 5.71 | | 0.00 | | 0.18 | | 1.00 | | 28.00 | | 9.00 | | 31.43 | |  |  |
| PLCB4 | 5.71 | | 0.00 | | 0.18 | | 1.00 | | 28.00 | | 9.00 | | 31.43 | |  |  |
| TLR8 | 5.71 | | 0.00 | | 0.18 | | 1.00 | | 28.00 | | 9.00 | | 31.43 | |  |  |
| HTRA1 | 5.71 | | 0.00 | | 0.18 | | 1.00 | | 28.00 | | 9.00 | | 31.43 | |  |  |
| IFNGR1 | 5.71 | | 0.00 | | 0.18 | | 1.00 | | 28.00 | | 9.00 | | 31.43 | |  |  |
| GNS | 5.71 | | 0.00 | | 0.18 | | 1.00 | | 28.00 | | 9.00 | | 31.43 | |  |  |
| DDX3X | 5.71 | | 0.00 | | 0.18 | | 1.00 | | 28.00 | | 9.00 | | 31.43 | |  |  |
| TRPM2 | 5.71 | | 0.00 | | 0.18 | | 1.00 | | 28.00 | | 9.00 | | 31.43 | |  |  |
| FBN1 | 5.71 | | 0.00 | | 0.18 | | 1.00 | | 28.00 | | 9.00 | | 31.43 | |  |  |
| Cd44 | 5.71 | | 0.00 | | 0.18 | | 1.00 | | 28.00 | | 9.00 | | 31.43 | |  |  |
| BANK1 | 5.71 | | 0.00 | | 0.18 | | 1.00 | | 28.00 | | 9.00 | | 31.43 | |  |  |
| DOCK10 | 5.71 | | 0.00 | | 0.18 | | 1.00 | | 28.00 | | 9.00 | | 31.43 | |  |  |
| ATP11B | 5.71 | | 0.00 | | 0.18 | | 1.00 | | 28.00 | | 9.00 | | 31.43 | |  |  |
| MMP7 | 5.71 | | 0.00 | | 0.18 | | 1.00 | | 28.00 | | 9.00 | | 31.43 | |  |  |
| LYZ | 5.71 | | 0.00 | | 0.18 | | 1.00 | | 28.00 | | 9.00 | | 31.43 | |  |  |
| TLR2 | 5.71 | | 0.00 | | 0.18 | | 1.00 | | 28.00 | | 9.00 | | 31.43 | |  |  |
| LTA | 4.18 | | 0.02 | | 0.24 | | 0.45 | | 28.00 | | 8.00 | | 16.57 | |  |  |
| DNASE1L3 | 5.71 | | 0.00 | | 0.18 | | 1.00 | | 28.00 | | 9.00 | | 31.43 | |  |  |
| EIF2B3 | 5.71 | | 0.00 | | 0.18 | | 1.00 | | 28.00 | | 9.00 | | 31.43 | |  |  |
| CD244 | 5.71 | | 0.00 | | 0.18 | | 1.00 | | 28.00 | | 9.00 | | 31.43 | |  |  |
| C9 | 5.71 | | 0.00 | | 0.18 | | 1.00 | | 28.00 | | 9.00 | | 31.43 | |  |  |
| RAB33A | 3.62 | | 0.10 | | 0.28 | | 0.51 | | 27.00 | | 6.00 | | 18.93 | |  |  |
| ARHGAP9 | 3.62 | | 0.10 | | 0.28 | | 0.51 | | 27.00 | | 6.00 | | 18.93 | |  |  |
| THEMIS | 4.31 | | 0.07 | | 0.23 | | 0.51 | | 26.00 | | 7.00 | | 18.19 | |  |  |
| CCL22 | 5.26 | | 0.00 | | 0.19 | | 1.00 | | 25.00 | | 10.00 | | 36.44 | |  |  |
| TRAV13-1 | 5.26 | | 0.00 | | 0.19 | | 1.00 | | 25.00 | | 10.00 | | 36.44 | |  |  |
| GSDMD | 5.26 | | 0.00 | | 0.19 | | 1.00 | | 25.00 | | 10.00 | | 36.44 | |  |  |
| RIPOR2 | 5.26 | | 0.00 | | 0.19 | | 1.00 | | 25.00 | | 10.00 | | 36.44 | |  |  |
| TICAM2 | 5.26 | | 0.00 | | 0.19 | | 1.00 | | 25.00 | | 10.00 | | 36.44 | |  |  |
| Pde4d | 5.26 | | 0.00 | | 0.19 | | 1.00 | | 25.00 | | 10.00 | | 36.44 | |  |  |
| APOA1 | 5.26 | | 0.00 | | 0.19 | | 1.00 | | 25.00 | | 10.00 | | 36.44 | |  |  |
| GMFG | 5.26 | | 0.00 | | 0.19 | | 1.00 | | 25.00 | | 10.00 | | 36.44 | |  |  |
| MMP9 | 5.26 | | 0.00 | | 0.19 | | 1.00 | | 25.00 | | 10.00 | | 36.44 | |  |  |
| GZMB | 5.26 | | 0.00 | | 0.19 | | 1.00 | | 25.00 | | 10.00 | | 36.44 | |  |  |
| RAB6A | 5.26 | | 0.00 | | 0.19 | | 1.00 | | 25.00 | | 10.00 | | 36.44 | |  |  |
| LAG3 | 5.26 | | 0.00 | | 0.19 | | 1.00 | | 25.00 | | 10.00 | | 36.44 | |  |  |
| EPHB6 | 5.26 | | 0.00 | | 0.19 | | 1.00 | | 25.00 | | 10.00 | | 36.44 | |  |  |
| DMTF1 | 5.26 | | 0.00 | | 0.19 | | 1.00 | | 25.00 | | 10.00 | | 36.44 | |  |  |
| SFRP1 | 5.26 | | 0.00 | | 0.19 | | 1.00 | | 25.00 | | 10.00 | | 36.44 | |  |  |
| CXCL10 | 5.26 | | 0.00 | | 0.19 | | 1.00 | | 25.00 | | 10.00 | | 36.44 | |  |  |
| Prkcz | 5.26 | | 0.00 | | 0.19 | | 1.00 | | 25.00 | | 10.00 | | 36.44 | |  |  |
| AICDA | 2.89 | | 0.01 | | 0.35 | | 0.38 | | 24.00 | | 6.00 | | 135.17 | |  |  |
| AGER | 3.61 | | 0.00 | | 0.28 | | 0.41 | | 24.00 | | 8.00 | | 98.75 | |  |  |
| PAK3 | 3.54 | | 0.26 | | 0.28 | | 0.49 | | 24.00 | | 5.00 | | 13.79 | |  |  |
| CXCL13 | 3.61 | | 0.00 | | 0.28 | | 0.41 | | 24.00 | | 8.00 | | 98.75 | |  |  |
| SLIT2 | 3.61 | | 0.00 | | 0.28 | | 0.41 | | 24.00 | | 8.00 | | 98.75 | |  |  |
| VPREB3 | 3.61 | | 0.00 | | 0.28 | | 0.41 | | 24.00 | | 8.00 | | 98.75 | |  |  |
| VPREB1 | 2.89 | | 0.01 | | 0.35 | | 0.38 | | 24.00 | | 6.00 | | 135.17 | |  |  |
| COCH | 3.61 | | 0.00 | | 0.28 | | 0.41 | | 24.00 | | 8.00 | | 98.75 | |  |  |
| CNR1 | 3.61 | | 0.00 | | 0.28 | | 0.41 | | 24.00 | | 8.00 | | 98.75 | |  |  |
| IL6 | 3.61 | | 0.00 | | 0.28 | | 0.41 | | 24.00 | | 8.00 | | 98.75 | |  |  |
| GH1 | 2.89 | | 0.01 | | 0.35 | | 0.38 | | 24.00 | | 6.00 | | 135.17 | |  |  |
| IL31RA | 2.89 | | 0.01 | | 0.35 | | 0.38 | | 24.00 | | 6.00 | | 135.17 | |  |  |
| SLIT2 | 3.61 | | 0.00 | | 0.28 | | 0.41 | | 24.00 | | 8.00 | | 98.75 | |  |  |
| SUCNR1 | 3.61 | | 0.00 | | 0.28 | | 0.41 | | 24.00 | | 8.00 | | 98.75 | |  |  |
| CXCL11 | 3.61 | | 0.00 | | 0.28 | | 0.41 | | 24.00 | | 8.00 | | 98.75 | |  |  |
| CLEC4E | 3.61 | | 0.00 | | 0.28 | | 0.41 | | 24.00 | | 8.00 | | 98.75 | |  |  |
| CLEC12A | 2.89 | | 0.01 | | 0.35 | | 0.38 | | 24.00 | | 6.00 | | 135.17 | |  |  |
| TAL1 | 3.61 | | 0.00 | | 0.28 | | 0.41 | | 24.00 | | 8.00 | | 98.75 | |  |  |
| C5 | 3.61 | | 0.00 | | 0.28 | | 0.41 | | 24.00 | | 8.00 | | 98.75 | |  |  |
| -- | 3.61 | | 0.00 | | 0.28 | | 0.41 | | 24.00 | | 8.00 | | 98.75 | |  |  |
| IGF1 | 2.89 | | 0.01 | | 0.35 | | 0.38 | | 24.00 | | 6.00 | | 135.17 | |  |  |
| CRHR1 | 2.89 | | 0.01 | | 0.35 | | 0.38 | | 24.00 | | 6.00 | | 135.17 | |  |  |
| DNASE1 | 3.61 | | 0.00 | | 0.28 | | 0.41 | | 24.00 | | 8.00 | | 98.75 | |  |  |
| IL21 | 2.89 | | 0.01 | | 0.35 | | 0.38 | | 24.00 | | 6.00 | | 135.17 | |  |  |
| Nr4a3 | 3.61 | | 0.00 | | 0.28 | | 0.41 | | 24.00 | | 8.00 | | 98.75 | |  |  |
| TRAV8-4 | 3.61 | | 0.00 | | 0.28 | | 0.41 | | 24.00 | | 8.00 | | 98.75 | |  |  |
| FOXJ1 | 3.61 | | 0.00 | | 0.28 | | 0.41 | | 24.00 | | 8.00 | | 98.75 | |  |  |
| TXK | 3.61 | | 0.00 | | 0.28 | | 0.41 | | 24.00 | | 8.00 | | 98.75 | |  |  |
| CARD9 | 5.74 | | 0.00 | | 0.17 | | 1.00 | | 20.00 | | 9.00 | | 26.40 | |  |  |
| C1QA | 5.74 | | 0.00 | | 0.17 | | 1.00 | | 20.00 | | 9.00 | | 26.40 | |  |  |
| TNFSF13 | 4.57 | | 0.00 | | 0.22 | | 1.00 | | 20.00 | | 9.00 | | 36.05 | |  |  |
| LRRC70 | 4.57 | | 0.00 | | 0.22 | | 1.00 | | 20.00 | | 9.00 | | 36.05 | |  |  |
| CTNNB1 | 5.74 | | 0.00 | | 0.17 | | 1.00 | | 20.00 | | 9.00 | | 26.40 | |  |  |
| Tgfb2 | 3.52 | | 0.08 | | 0.28 | | 0.72 | | 20.00 | | 5.00 | | 16.90 | |  |  |
| GAB2 | 5.74 | | 0.00 | | 0.17 | | 1.00 | | 20.00 | | 9.00 | | 26.40 | |  |  |
| PROS1 | 4.57 | | 0.00 | | 0.22 | | 1.00 | | 20.00 | | 9.00 | | 36.05 | |  |  |
| S100A14 | 4.57 | | 0.00 | | 0.22 | | 1.00 | | 20.00 | | 9.00 | | 36.05 | |  |  |
| RAB3B | 4.57 | | 0.00 | | 0.22 | | 1.00 | | 20.00 | | 9.00 | | 36.05 | |  |  |
| ADORA1 | 4.57 | | 0.00 | | 0.22 | | 1.00 | | 20.00 | | 9.00 | | 36.05 | |  |  |
| BCR | 4.57 | | 0.00 | | 0.22 | | 1.00 | | 20.00 | | 9.00 | | 36.05 | |  |  |
| APBB1IP | 4.57 | | 0.00 | | 0.22 | | 1.00 | | 20.00 | | 9.00 | | 36.05 | |  |  |
| FSTL3 | 5.74 | | 0.00 | | 0.17 | | 1.00 | | 20.00 | | 9.00 | | 26.40 | |  |  |
| Ms4a2 | 5.74 | | 0.00 | | 0.17 | | 1.00 | | 20.00 | | 9.00 | | 26.40 | |  |  |
| TRBV12-4 | 5.02 | | 0.05 | | 0.20 | | 0.24 | | 20.00 | | 8.00 | | 7.30 | |  |  |
| KIF5A | 3.52 | | 0.08 | | 0.28 | | 0.72 | | 20.00 | | 5.00 | | 16.90 | |  |  |
| CEBPG | 5.74 | | 0.00 | | 0.17 | | 1.00 | | 20.00 | | 9.00 | | 26.40 | |  |  |
| LAX1 | 4.57 | | 0.00 | | 0.22 | | 1.00 | | 20.00 | | 9.00 | | 36.05 | |  |  |
| C8G | 5.74 | | 0.00 | | 0.17 | | 1.00 | | 20.00 | | 9.00 | | 26.40 | |  |  |
| HCK | 5.74 | | 0.00 | | 0.17 | | 1.00 | | 20.00 | | 9.00 | | 26.40 | |  |  |
| PLAU | 5.74 | | 0.00 | | 0.17 | | 1.00 | | 20.00 | | 9.00 | | 26.40 | |  |  |
| ADA2 | 5.74 | | 0.00 | | 0.17 | | 1.00 | | 20.00 | | 9.00 | | 26.40 | |  |  |
| TNFRSF17 | 5.74 | | 0.00 | | 0.17 | | 1.00 | | 20.00 | | 9.00 | | 26.40 | |  |  |
| CD3D | 4.57 | | 0.00 | | 0.22 | | 1.00 | | 20.00 | | 9.00 | | 36.05 | |  |  |
| TRAV16 | 3.52 | | 0.08 | | 0.28 | | 0.72 | | 20.00 | | 5.00 | | 16.90 | |  |  |
| ADM | 4.57 | | 0.00 | | 0.22 | | 1.00 | | 20.00 | | 9.00 | | 36.05 | |  |  |
| NOD2 | 4.57 | | 0.00 | | 0.22 | | 1.00 | | 20.00 | | 9.00 | | 36.05 | |  |  |
| STK11IP | 5.74 | | 0.00 | | 0.17 | | 1.00 | | 20.00 | | 9.00 | | 26.40 | |  |  |
| PTPRJ | 5.74 | | 0.00 | | 0.17 | | 1.00 | | 20.00 | | 9.00 | | 26.40 | |  |  |
| C1QB | 5.74 | | 0.00 | | 0.17 | | 1.00 | | 20.00 | | 9.00 | | 26.40 | |  |  |
| Dctn4 | 5.74 | | 0.00 | | 0.17 | | 1.00 | | 20.00 | | 9.00 | | 26.40 | |  |  |
| Tcf7 | 4.57 | | 0.00 | | 0.22 | | 1.00 | | 20.00 | | 9.00 | | 36.05 | |  |  |
| TLR5 | 5.74 | | 0.00 | | 0.17 | | 1.00 | | 20.00 | | 9.00 | | 26.40 | |  |  |
| GAPT | 4.57 | | 0.00 | | 0.22 | | 1.00 | | 20.00 | | 9.00 | | 36.05 | |  |  |
| SAMSN1 | 4.37 | | 0.01 | | 0.23 | | 0.56 | | 18.00 | | 7.00 | | 15.78 | |  |  |
| TRIM6 | 4.37 | | 0.01 | | 0.23 | | 0.56 | | 18.00 | | 7.00 | | 15.78 | |  |  |
| TRAF3IP1 | 4.37 | | 0.01 | | 0.23 | | 0.56 | | 18.00 | | 7.00 | | 15.78 | |  |  |
| RIPK2 | 3.74 | | 0.01 | | 0.27 | | 0.99 | | 18.00 | | 6.00 | | 22.56 | |  |  |
| CD96 | 3.74 | | 0.01 | | 0.27 | | 0.99 | | 18.00 | | 6.00 | | 22.56 | |  |  |
| IGF2 | 3.74 | | 0.01 | | 0.27 | | 0.99 | | 18.00 | | 6.00 | | 22.56 | |  |  |
| NMI | 3.74 | | 0.01 | | 0.27 | | 0.99 | | 18.00 | | 6.00 | | 22.56 | |  |  |
| PTPRS | 3.74 | | 0.01 | | 0.27 | | 0.99 | | 18.00 | | 6.00 | | 22.56 | |  |  |
| BMI1 | 3.74 | | 0.01 | | 0.27 | | 0.99 | | 18.00 | | 6.00 | | 22.56 | |  |  |
| Grap2 | 3.74 | | 0.01 | | 0.27 | | 0.99 | | 18.00 | | 6.00 | | 22.56 | |  |  |
| IGLV8-61 | 3.74 | | 0.01 | | 0.27 | | 0.99 | | 18.00 | | 6.00 | | 22.56 | |  |  |
| GAS6 | 3.74 | | 0.01 | | 0.27 | | 0.99 | | 18.00 | | 6.00 | | 22.56 | |  |  |
| PLA2G1B | 3.74 | | 0.01 | | 0.27 | | 0.99 | | 18.00 | | 6.00 | | 22.56 | |  |  |
| CD6 | 3.74 | | 0.01 | | 0.27 | | 0.99 | | 18.00 | | 6.00 | | 22.56 | |  |  |
| GPR68 | 3.74 | | 0.01 | | 0.27 | | 0.99 | | 18.00 | | 6.00 | | 22.56 | |  |  |
| CDK11B | 3.74 | | 0.01 | | 0.27 | | 0.99 | | 18.00 | | 6.00 | | 22.56 | |  |  |
| EIF2AK2 | 3.74 | | 0.01 | | 0.27 | | 0.99 | | 18.00 | | 6.00 | | 22.56 | |  |  |
| GPRC5B | 3.74 | | 0.01 | | 0.27 | | 0.99 | | 18.00 | | 6.00 | | 22.56 | |  |  |
| TYROBP | 4.49 | | 0.00 | | 0.22 | | 1.00 | | 17.00 | | 9.00 | | 37.59 | |  |  |
| IRAK2 | 4.49 | | 0.00 | | 0.22 | | 1.00 | | 17.00 | | 9.00 | | 37.59 | |  |  |
| CD37 | 4.49 | | 0.00 | | 0.22 | | 1.00 | | 17.00 | | 9.00 | | 37.59 | |  |  |
| MGST1 | 4.49 | | 0.00 | | 0.22 | | 1.00 | | 17.00 | | 9.00 | | 37.59 | |  |  |
| MUC2 | 4.49 | | 0.00 | | 0.22 | | 1.00 | | 17.00 | | 9.00 | | 37.59 | |  |  |
| IGLV8-61 | 4.49 | | 0.00 | | 0.22 | | 1.00 | | 17.00 | | 9.00 | | 37.59 | |  |  |
| TFEB | 4.49 | | 0.00 | | 0.22 | | 1.00 | | 17.00 | | 9.00 | | 37.59 | |  |  |
| FCER2 | 4.49 | | 0.00 | | 0.22 | | 1.00 | | 17.00 | | 9.00 | | 37.59 | |  |  |
| KLRD1 | 4.49 | | 0.00 | | 0.22 | | 1.00 | | 17.00 | | 9.00 | | 37.59 | |  |  |
| CCDC194 | 4.49 | | 0.00 | | 0.22 | | 1.00 | | 17.00 | | 9.00 | | 37.59 | |  |  |
| VIP | 3.80 | | 0.00 | | 0.26 | | 1.00 | | 16.00 | | 5.00 | | 18.63 | |  |  |
| PLEKHA1 | 3.80 | | 0.00 | | 0.26 | | 1.00 | | 16.00 | | 5.00 | | 18.63 | |  |  |
| COMMD9 | 4.96 | | 0.00 | | 0.20 | | 1.00 | | 16.00 | | 9.00 | | 21.25 | |  |  |
| RORC | 4.96 | | 0.00 | | 0.20 | | 1.00 | | 16.00 | | 9.00 | | 21.25 | |  |  |
| MMP12 | 4.96 | | 0.00 | | 0.20 | | 1.00 | | 16.00 | | 9.00 | | 21.25 | |  |  |
| KLRK1 | 5.13 | | 0.00 | | 0.19 | | 1.00 | | 16.00 | | 8.00 | | 21.31 | |  |  |
| MMP25 | 5.13 | | 0.00 | | 0.19 | | 1.00 | | 16.00 | | 8.00 | | 21.31 | |  |  |
| Pum1 | 3.80 | | 0.00 | | 0.26 | | 1.00 | | 16.00 | | 5.00 | | 18.63 | |  |  |
| CMTM6 | 3.80 | | 0.00 | | 0.26 | | 1.00 | | 16.00 | | 5.00 | | 18.63 | |  |  |
| RPS6 | 3.80 | | 0.00 | | 0.26 | | 1.00 | | 16.00 | | 5.00 | | 18.63 | |  |  |
| DDX60 | 3.80 | | 0.00 | | 0.26 | | 1.00 | | 16.00 | | 5.00 | | 18.63 | |  |  |
| GFER | 5.13 | | 0.00 | | 0.19 | | 1.00 | | 16.00 | | 8.00 | | 21.31 | |  |  |
| RBP4 | 4.96 | | 0.00 | | 0.20 | | 1.00 | | 16.00 | | 9.00 | | 21.25 | |  |  |
| PDZD2 | 5.13 | | 0.00 | | 0.19 | | 1.00 | | 16.00 | | 8.00 | | 21.31 | |  |  |
| CTSF | 4.96 | | 0.00 | | 0.20 | | 1.00 | | 16.00 | | 9.00 | | 21.25 | |  |  |
| C5 | 3.80 | | 0.00 | | 0.26 | | 1.00 | | 16.00 | | 5.00 | | 18.63 | |  |  |
| ANGPT1 | 5.13 | | 0.00 | | 0.19 | | 1.00 | | 16.00 | | 8.00 | | 21.31 | |  |  |
| ADAM8 | 5.13 | | 0.00 | | 0.19 | | 1.00 | | 16.00 | | 8.00 | | 21.31 | |  |  |
| CD27 | 5.13 | | 0.00 | | 0.19 | | 1.00 | | 16.00 | | 8.00 | | 21.31 | |  |  |
| TGFBR2 | 3.80 | | 0.00 | | 0.26 | | 1.00 | | 16.00 | | 5.00 | | 18.63 | |  |  |
| CD226 | 5.13 | | 0.00 | | 0.19 | | 1.00 | | 16.00 | | 8.00 | | 21.31 | |  |  |
| CEP290 | 5.13 | | 0.00 | | 0.19 | | 1.00 | | 16.00 | | 8.00 | | 21.31 | |  |  |
| PRDX5 | 4.96 | | 0.00 | | 0.20 | | 1.00 | | 16.00 | | 9.00 | | 21.25 | |  |  |
| DDX60 | 3.80 | | 0.00 | | 0.26 | | 1.00 | | 16.00 | | 5.00 | | 18.63 | |  |  |
| C6 | 3.80 | | 0.00 | | 0.26 | | 1.00 | | 16.00 | | 5.00 | | 18.63 | |  |  |
| IL7 | 3.80 | | 0.00 | | 0.26 | | 1.00 | | 16.00 | | 5.00 | | 18.63 | |  |  |
| PDE5A | 3.80 | | 0.00 | | 0.26 | | 1.00 | | 16.00 | | 5.00 | | 18.63 | |  |  |
| JUN | 4.96 | | 0.00 | | 0.20 | | 1.00 | | 16.00 | | 9.00 | | 21.25 | |  |  |
| IGLV8-61 | 5.13 | | 0.00 | | 0.19 | | 1.00 | | 16.00 | | 8.00 | | 21.31 | |  |  |
| FADS3 | 4.96 | | 0.00 | | 0.20 | | 1.00 | | 16.00 | | 9.00 | | 21.25 | |  |  |
| CIB1 | 5.13 | | 0.00 | | 0.19 | | 1.00 | | 16.00 | | 8.00 | | 21.31 | |  |  |
| BCL6B | 4.96 | | 0.00 | | 0.20 | | 1.00 | | 16.00 | | 9.00 | | 21.25 | |  |  |
| IFI30 | 4.96 | | 0.00 | | 0.20 | | 1.00 | | 16.00 | | 9.00 | | 21.25 | |  |  |
| C4BPA | 4.96 | | 0.00 | | 0.20 | | 1.00 | | 16.00 | | 9.00 | | 21.25 | |  |  |
| RAB9B | 4.54 | | 0.04 | | 0.22 | | 0.29 | | 16.00 | | 7.00 | | 7.94 | |  |  |
| GAL | 3.98 | | 0.04 | | 0.25 | | 0.50 | | 15.00 | | 6.00 | | 13.13 | |  |  |
| IL12A | 3.56 | | 0.36 | | 0.28 | | 0.40 | | 15.00 | | 6.00 | | 33.33 | |  |  |
| LAT | 5.57 | | 0.00 | | 0.18 | | 1.00 | | 14.00 | | 9.00 | | 24.93 | |  |  |
| CLEC10A | 4.14 | | 0.03 | | 0.24 | | 0.47 | | 14.00 | | 7.00 | | 43.71 | |  |  |
| SIGLEC14 | 5.57 | | 0.00 | | 0.18 | | 1.00 | | 14.00 | | 9.00 | | 24.93 | |  |  |
| ADIPOQ | 4.14 | | 0.03 | | 0.24 | | 0.47 | | 14.00 | | 7.00 | | 43.71 | |  |  |
| LEF1 | 5.57 | | 0.00 | | 0.18 | | 1.00 | | 14.00 | | 9.00 | | 24.93 | |  |  |
| LPCAT1 | 5.12 | | 0.00 | | 0.20 | | 1.00 | | 14.00 | | 9.00 | | 20.57 | |  |  |
| Eomes | 5.57 | | 0.00 | | 0.18 | | 1.00 | | 14.00 | | 9.00 | | 24.93 | |  |  |
| Clec2d11 | 5.57 | | 0.00 | | 0.18 | | 1.00 | | 14.00 | | 9.00 | | 24.93 | |  |  |
| LST1 | 4.14 | | 0.03 | | 0.24 | | 0.47 | | 14.00 | | 7.00 | | 43.71 | |  |  |
| REV1 | 5.57 | | 0.00 | | 0.18 | | 1.00 | | 14.00 | | 9.00 | | 24.93 | |  |  |
| Rgcc | 5.57 | | 0.00 | | 0.18 | | 1.00 | | 14.00 | | 9.00 | | 24.93 | |  |  |
| NOTCH2 | 5.12 | | 0.00 | | 0.20 | | 1.00 | | 14.00 | | 9.00 | | 20.57 | |  |  |
| CREBBP | 5.12 | | 0.00 | | 0.20 | | 1.00 | | 14.00 | | 9.00 | | 20.57 | |  |  |
| Pol | 4.14 | | 0.03 | | 0.24 | | 0.47 | | 14.00 | | 7.00 | | 43.71 | |  |  |
| TRAV23DV6 | 4.14 | | 0.03 | | 0.24 | | 0.47 | | 14.00 | | 7.00 | | 43.71 | |  |  |
| JAM3 | 5.57 | | 0.00 | | 0.18 | | 1.00 | | 14.00 | | 9.00 | | 24.93 | |  |  |
| FGR | 5.12 | | 0.00 | | 0.20 | | 1.00 | | 14.00 | | 9.00 | | 20.57 | |  |  |
| LGALS1 | 5.12 | | 0.00 | | 0.20 | | 1.00 | | 14.00 | | 9.00 | | 20.57 | |  |  |
| SHLD2 | 5.12 | | 0.00 | | 0.20 | | 1.00 | | 14.00 | | 9.00 | | 20.57 | |  |  |
| ACLY | 5.12 | | 0.00 | | 0.20 | | 1.00 | | 14.00 | | 9.00 | | 20.57 | |  |  |
| TNF | 5.12 | | 0.00 | | 0.20 | | 1.00 | | 14.00 | | 9.00 | | 20.57 | |  |  |
| Huwe1 | 5.12 | | 0.00 | | 0.20 | | 1.00 | | 14.00 | | 9.00 | | 20.57 | |  |  |
| C5AR1 | 5.12 | | 0.00 | | 0.20 | | 1.00 | | 14.00 | | 9.00 | | 20.57 | |  |  |
| ATM | 5.12 | | 0.00 | | 0.20 | | 1.00 | | 14.00 | | 9.00 | | 20.57 | |  |  |
| CYBB | 5.12 | | 0.00 | | 0.20 | | 1.00 | | 14.00 | | 9.00 | | 20.57 | |  |  |
| THEMIS2 | 4.26 | | 0.00 | | 0.24 | | 1.00 | | 12.00 | | 8.00 | | 23.00 | |  |  |
| APAF1 | 4.26 | | 0.00 | | 0.24 | | 1.00 | | 12.00 | | 8.00 | | 23.00 | |  |  |
| TNFAIP3 | 4.26 | | 0.00 | | 0.24 | | 1.00 | | 12.00 | | 8.00 | | 23.00 | |  |  |
| TLR1 | 4.26 | | 0.00 | | 0.24 | | 1.00 | | 12.00 | | 8.00 | | 23.00 | |  |  |
| MAFB | 4.26 | | 0.00 | | 0.24 | | 1.00 | | 12.00 | | 8.00 | | 23.00 | |  |  |
| MAPK1 | 4.26 | | 0.00 | | 0.24 | | 1.00 | | 12.00 | | 8.00 | | 23.00 | |  |  |
| LCN2 | 4.26 | | 0.00 | | 0.24 | | 1.00 | | 12.00 | | 8.00 | | 23.00 | |  |  |
| IFIH1 | 5.13 | | 0.00 | | 0.19 | | 1.00 | | 10.00 | | 8.00 | | 20.80 | |  |  |
| CD1A | 5.13 | | 0.00 | | 0.19 | | 1.00 | | 10.00 | | 8.00 | | 20.80 | |  |  |
| GAA | 5.13 | | 0.00 | | 0.19 | | 1.00 | | 10.00 | | 8.00 | | 20.80 | |  |  |
| NAA25 | 5.13 | | 0.00 | | 0.19 | | 1.00 | | 10.00 | | 8.00 | | 20.80 | |  |  |
| ZBTB16 | 5.13 | | 0.00 | | 0.19 | | 1.00 | | 10.00 | | 8.00 | | 20.80 | |  |  |
| WNT5A | 4.84 | | 0.00 | | 0.21 | | 1.00 | | 9.00 | | 7.00 | | 12.67 | |  |  |
| NOL6 | 4.84 | | 0.00 | | 0.21 | | 1.00 | | 9.00 | | 7.00 | | 12.67 | |  |  |
| PGLYRP2 | 4.84 | | 0.00 | | 0.21 | | 1.00 | | 9.00 | | 7.00 | | 12.67 | |  |  |
| PLA2G2D | 4.86 | | 0.00 | | 0.21 | | 0.61 | | 9.00 | | 9.00 | | 34.89 | |  |  |
| MUC15 | 4.86 | | 0.00 | | 0.21 | | 0.61 | | 9.00 | | 9.00 | | 34.89 | |  |  |
| PKP1 | 3.58 | | 0.33 | | 0.28 | | 0.00 | | 9.00 | | 6.00 | | 9.78 | |  |  |
| PIK3R6 | 4.84 | | 0.00 | | 0.21 | | 1.00 | | 9.00 | | 7.00 | | 12.67 | |  |  |
| JAG1 | 4.84 | | 0.00 | | 0.21 | | 1.00 | | 9.00 | | 7.00 | | 12.67 | |  |  |
| LRRC7 | 4.86 | | 0.00 | | 0.21 | | 0.61 | | 9.00 | | 9.00 | | 34.89 | |  |  |
| RAB34 | 4.84 | | 0.00 | | 0.21 | | 1.00 | | 9.00 | | 7.00 | | 12.67 | |  |  |
| MASP1 | 4.86 | | 0.00 | | 0.21 | | 0.61 | | 9.00 | | 9.00 | | 34.89 | |  |  |
| CLEC5A | 4.86 | | 0.00 | | 0.21 | | 0.61 | | 9.00 | | 9.00 | | 34.89 | |  |  |
| IL13RA2 | 4.86 | | 0.00 | | 0.21 | | 0.61 | | 9.00 | | 9.00 | | 34.89 | |  |  |
| Cd38 | 1.00 | | 0.00 | | 1.00 | | 1.00 | | 8.00 | | 1.00 | | 8.00 | |  |  |
| Spns2 | 1.00 | | 0.00 | | 1.00 | | 1.00 | | 8.00 | | 1.00 | | 8.00 | |  |  |
| VNN1 | 5.50 | | 0.00 | | 0.18 | | 1.00 | | 8.00 | | 8.00 | | 10.50 | |  |  |
| NLRP3 | 5.50 | | 0.00 | | 0.18 | | 1.00 | | 8.00 | | 8.00 | | 10.50 | |  |  |
| Pol | 1.00 | | 0.00 | | 1.00 | | 1.00 | | 8.00 | | 1.00 | | 8.00 | |  |  |
| HERC5 | 1.00 | | 0.00 | | 1.00 | | 1.00 | | 8.00 | | 1.00 | | 8.00 | |  |  |
| DHRS1 | 5.50 | | 0.00 | | 0.18 | | 1.00 | | 8.00 | | 8.00 | | 10.50 | |  |  |
| Clcf1 | 1.00 | | 0.00 | | 1.00 | | 1.00 | | 8.00 | | 1.00 | | 8.00 | |  |  |
| POLR3B | 1.00 | | 0.00 | | 1.00 | | 1.00 | | 8.00 | | 1.00 | | 8.00 | |  |  |
| EMILIN1 | 5.50 | | 0.00 | | 0.18 | | 1.00 | | 8.00 | | 8.00 | | 10.50 | |  |  |
| LOXL3 | 5.50 | | 0.00 | | 0.18 | | 1.00 | | 8.00 | | 8.00 | | 10.50 | |  |  |
| RPS6KA5 | 1.00 | | 0.00 | | 1.00 | | 1.00 | | 8.00 | | 1.00 | | 8.00 | |  |  |
| CFP | 1.00 | | 0.00 | | 1.00 | | 1.00 | | 8.00 | | 1.00 | | 8.00 | |  |  |
| AP2S1 | 5.50 | | 0.00 | | 0.18 | | 1.00 | | 8.00 | | 8.00 | | 10.50 | |  |  |
| LIME1 | 1.00 | | 0.00 | | 1.00 | | 1.00 | | 8.00 | | 1.00 | | 8.00 | |  |  |
| KIF3C | 5.50 | | 0.00 | | 0.18 | | 1.00 | | 8.00 | | 8.00 | | 10.50 | |  |  |
| NFKBIL1 | 4.44 | | 0.00 | | 0.23 | | 1.00 | | 7.00 | | 8.00 | | 18.71 | |  |  |
| APOB | 4.16 | | 0.04 | | 0.24 | | 0.33 | | 7.00 | | 7.00 | | 36.14 | |  |  |
| HLX | 3.78 | | 0.01 | | 0.26 | | 0.90 | | 7.00 | | 5.00 | | 20.57 | |  |  |
| ALOX15 | 4.16 | | 0.04 | | 0.24 | | 0.33 | | 7.00 | | 7.00 | | 36.14 | |  |  |
| NAPRT | 4.44 | | 0.00 | | 0.23 | | 1.00 | | 7.00 | | 8.00 | | 18.71 | |  |  |
| Dusp10 | 4.44 | | 0.00 | | 0.23 | | 1.00 | | 7.00 | | 8.00 | | 18.71 | |  |  |
| ADCYAP1 | 4.16 | | 0.04 | | 0.24 | | 0.33 | | 7.00 | | 7.00 | | 36.14 | |  |  |
| NLRX1 | 4.44 | | 0.00 | | 0.23 | | 1.00 | | 7.00 | | 8.00 | | 18.71 | |  |  |
| TRBV7-9 | 4.16 | | 0.04 | | 0.24 | | 0.33 | | 7.00 | | 7.00 | | 36.14 | |  |  |
| IL23R | 4.16 | | 0.04 | | 0.24 | | 0.33 | | 7.00 | | 7.00 | | 36.14 | |  |  |
| CCL25 | 4.16 | | 0.04 | | 0.24 | | 0.33 | | 7.00 | | 7.00 | | 36.14 | |  |  |
| MMP28 | 4.44 | | 0.00 | | 0.23 | | 1.00 | | 7.00 | | 8.00 | | 18.71 | |  |  |
| MAPK10 | 4.16 | | 0.04 | | 0.24 | | 0.33 | | 7.00 | | 7.00 | | 36.14 | |  |  |
| MEIS1 | 3.78 | | 0.01 | | 0.26 | | 0.90 | | 7.00 | | 5.00 | | 20.57 | |  |  |
| TNFSF13B | 3.93 | | 0.01 | | 0.25 | | 0.93 | | 6.00 | | 6.00 | | 21.67 | |  |  |
| COLEC12 | 3.93 | | 0.01 | | 0.25 | | 0.93 | | 6.00 | | 6.00 | | 21.67 | |  |  |
| RORA | 3.93 | | 0.01 | | 0.25 | | 0.93 | | 6.00 | | 6.00 | | 21.67 | |  |  |
| ZBTB46 | 6.00 | | 0.00 | | 0.17 | | 1.00 | | 5.00 | | 9.00 | | 8.00 | |  |  |
| BCL3 | 6.00 | | 0.00 | | 0.17 | | 1.00 | | 5.00 | | 9.00 | | 8.00 | |  |  |
| FCRL5 | 6.00 | | 0.00 | | 0.17 | | 1.00 | | 5.00 | | 9.00 | | 8.00 | |  |  |
| EBI3 | 6.00 | | 0.00 | | 0.17 | | 1.00 | | 5.00 | | 9.00 | | 8.00 | |  |  |
| NLRC4 | 6.00 | | 0.00 | | 0.17 | | 1.00 | | 5.00 | | 9.00 | | 8.00 | |  |  |
| AGL | 4.52 | | 0.00 | | 0.22 | | 1.00 | | 4.00 | | 6.00 | | 9.00 | |  |  |
| C3 | 4.52 | | 0.00 | | 0.22 | | 1.00 | | 4.00 | | 6.00 | | 9.00 | |  |  |
| CLEC7A | 4.52 | | 0.00 | | 0.22 | | 1.00 | | 4.00 | | 6.00 | | 9.00 | |  |  |
| CFH | 4.52 | | 0.00 | | 0.22 | | 1.00 | | 4.00 | | 6.00 | | 9.00 | |  |  |
| H2B-I | 4.25 | | 0.03 | | 0.24 | | 0.00 | | 2.00 | | 6.00 | | 20.00 | |  |  |
| DCST1 | 4.37 | | 0.00 | | 0.23 | | 0.00 | | 1.00 | | 8.00 | | 48.00 | |  |  |
