# Supplementary material for: Integrated omics analysis reveals the immunologic characteristics of cystic Peyer’s patches in the cecum of Bactrian camels
Source: PeerJ. 2023 Jan 9;11:e14647. doi: 10.7717/peerj.14647 (PMC9835693; doi:10.7717/peerj.14647)
Supplement: Table S4 [file peerj-11-14647-s004.docx]

Table S4. Information of 113 differential abundant metabolites

| Compound_ID | NPPS  (mean) | PPS  (mean) | log2_FC  (PPS/NPPS) | FDR | Vip value | Name |
| --- | --- | --- | --- | --- | --- | --- |
| M537T175_POS | 7115.61 | 131946.38 | 4.21 | 0.26 | 1.31 | Telmisartan |
| M100T56_POS | 265509.54 | 2046250.90 | 2.95 | 0.06 | 5.41 | 2-piperidone |
| M568T157_POS | 23169.54 | 156825.50 | 2.76 | 0.25 | 1.34 | 1-Stearoyl-sn-glycerol 3-phosphocholine |
| M146T370_2_POS | 1720109.41 | 9931868.08 | 2.53 | 0.07 | 11.72 | Deoxycarnitine |
| M309T38_NEG | 204053.35 | 755007.76 | 1.89 | 0.27 | 2.83 | Eicosenoic acid |
| M86T261_POS | 58972.90 | 213212.71 | 1.85 | 0.11 | 1.57 | 1,5-pentanediamine |
| M563T171_POS | 56047.40 | 177816.53 | 1.67 | 0.08 | 1.38 | Protoporphyrin ix |
| M328T35_POS | 110626.06 | 304725.86 | 1.46 | 0.23 | 1.68 | Stearoyl ethanolamide |
| M255T46_NEG | 9114555.78 | 20593210.02 | 1.18 | 0.28 | 12.64 | Palmitic acid |
| M227T114_NEG | 137167.62 | 274519.61 | 1.00 | 0.08 | 1.48 | Ile-Pro |
| M143T132_NEG | 71048.27 | 138448.75 | 0.96 | 0.14 | 1.06 | Ammeline |
| M241T101_NEG | 308555.02 | 452319.28 | 0.55 | 0.06 | 1.55 | His-ser |
| M141T347_2_NEG | 12756542.08 | 7481904.17 | -0.77 | 0.12 | 9.12 | Cis,cis-muconic acid |
| M181T348_NEG | 562441.56 | 310826.20 | -0.86 | 0.10 | 2.02 | 3,4-dihydroxyhydrocinnamic acid |
| M288T38_POS | 1816931.61 | 988924.97 | -0.88 | 0.21 | 3.61 | C17-sphinganine |
| M295T489_NEG | 794230.58 | 425212.99 | -0.90 | 0.15 | 2.40 | Propanoic acid, 3-[[[2-[(aminoiminomethyl)amino]-4-thiazolyl]methyl]thio]- |
| M454T191_POS | 359614.00 | 186858.31 | -0.94 | 0.14 | 1.64 | 1-palmitoyl-2-hydroxy-sn-glycero-3-phosphoethanolamine |
| M229T295_POS | 423391.32 | 219686.21 | -0.95 | 0.13 | 1.79 | Pro-leu |
| M187T338_2_NEG | 301475.69 | 141547.58 | -1.09 | 0.15 | 1.58 | Azelaic acid |
| M309T25_NEG | 252190.86 | 116275.84 | -1.12 | 0.29 | 1.36 | Mestranol |
| M131T122_NEG | 319108.19 | 128060.07 | -1.32 | 0.29 | 1.64 | 6-hydroxyhexanoate |
| M365T360_POS | 106754.94 | 42214.15 | -1.34 | 0.07 | 1.04 | Melibiose |
| M434T376_POS | 141719.88 | 54624.90 | -1.38 | 0.16 | 1.11 | Fluvastatin |
| M395T33_2_POS | 145834.38 | 56086.47 | -1.38 | 0.08 | 1.21 | Fucosterol |
| M399T536_POS | 742724.47 | 284662.28 | -1.38 | 0.14 | 2.66 | S-adenosyl-l-methionine |
| M337T27_2_NEG | 153773.55 | 57570.01 | -1.42 | 0.19 | 1.24 | 18-carboxydinorleukotriene b4 |
| M480T188_POS | 1532850.03 | 551804.55 | -1.47 | 0.22 | 3.64 | 1-oleoyl-sn-glycero-3-phosphoethanolamine |
| M348T250_POS | 217115.71 | 77763.80 | -1.48 | 0.07 | 1.48 | 2'-deoxyguanosine 5'-monophosphate |
| M391T32_2_NEG | 146078.93 | 51364.64 | -1.51 | 0.09 | 1.24 | Dodecanoic acid, 12-[[(tricyclo[3.3.1.13,7]dec-1-ylamino)carbonyl]amino]- |
| M216T74_POS | 117665.33 | 40677.06 | -1.53 | 0.14 | 1.10 | Scopoline |
| M747T38_1_POS | 868404.20 | 294335.91 | -1.56 | 0.22 | 2.85 | 1-hexadecyl-2-(9z-octadecenoyl)-sn-glycero-3-phosphocholine |
| M293T421_POS | 173862.97 | 58672.41 | -1.57 | 0.11 | 1.35 | Ethylenediaminetetraacetic acid |
| M149T101_2_NEG | 6576650.90 | 2131687.89 | -1.63 | 0.10 | 8.58 | 3-methylphenylacetic acid |
| M375T84_NEG | 15664414.60 | 5063543.19 | -1.63 | 0.13 | 12.94 | Lithocholic acid |
| M391T159_POS | 128727.55 | 41509.20 | -1.63 | 0.13 | 1.16 | .gamma.-muricholic acid |
| M449T172_NEG | 166833.29 | 53058.11 | -1.65 | 0.11 | 1.34 | Vitamin k1 |
| M277T42_2_NEG | 585946.17 | 178986.84 | -1.71 | 0.25 | 2.33 | all cis-(6,9,12)-Linolenic acid |
| M211T83_POS | 93755.64 | 28558.83 | -1.71 | 0.06 | 1.05 | Jasmonic acid |
| M331T159_NEG | 177129.34 | 53643.12 | -1.72 | 0.11 | 1.42 | 21-Hydroxypregnenolone |
| M411T27_2_NEG | 86097.14 | 25827.95 | -1.74 | 0.06 | 1.00 | Pentanoic acid, 5-[(3ar,4r,5r,6as)-3,3-difluorohexahydro-5-hydroxy-4-[(1e,3s,4s)-3-hydroxy-4-methyl-1-nonen-6-ynyl]-2h-cyclopenta[b]furan-2-ylidene]-, (5z)- |
| M337T36_1_NEG | 550067.98 | 159681.13 | -1.78 | 0.22 | 2.47 | 2-(2-hydroxybut-3-en-2-yl)-3a,6,6,9a-tetramethyl-2,4,5,5a,7,8,9,9b-octahydro-1h-benzo[e][1]benzofuran-4,5-diol |
| M363T178_NEG | 178004.11 | 51277.63 | -1.80 | 0.06 | 1.45 | 9s,11r,15s-trihydroxy-20a,20b-dihomo-5z,13e-prostadienoic acid |
| M195T447_NEG | 116063.17 | 33032.95 | -1.81 | 0.07 | 1.19 | Galactonic acid |
| M477T86_NEG | 137486.74 | 38989.33 | -1.82 | 0.17 | 1.21 | 1,4-bis[(p-hydroxyphenethyl)amino]anthraquinone |
| M457T24_NEG | 883809.10 | 250440.26 | -1.82 | 0.07 | 3.27 | Andrastin c |
| M482T187_POS | 1758578.06 | 488328.85 | -1.85 | 0.23 | 4.13 | 1-Stearoyl-2-hydroxy-sn-glycero-3-phosphoethanolamine |
| M302T208_POS | 97709.63 | 26683.56 | -1.87 | 0.11 | 1.05 | Dihydrocodeine |
| M434T182_NEG | 88472.01 | 24117.29 | -1.88 | 0.06 | 1.03 | N-acetyl-s-geranylgeranyl-l-cysteine |
| M189T380_2_POS | 268842.21 | 72241.45 | -1.90 | 0.10 | 1.75 | N-acetylglutamine |
| M173T235_2_POS | 192414.87 | 51034.12 | -1.91 | 0.09 | 1.49 | L-Isoleucine |
| M195T156_POS | 137862.73 | 36336.57 | -1.92 | 0.07 | 1.27 | Dodecanedioic acid |
| M329T119_NEG | 116933.25 | 30701.75 | -1.93 | 0.16 | 1.11 | 11beta-hydroxyprogesterone |
| M403T65_NEG | 108068.87 | 27927.75 | -1.95 | 0.14 | 1.07 | 1-o-(9z-octadecenyl)-sn-glycero-2,3-cyclic-phosphate |
| M182T38_NEG | 120629.59 | 31048.78 | -1.96 | 0.24 | 1.17 | 4-pyridoxic acid |
| M332T180_POS | 455819.17 | 112798.78 | -2.01 | 0.21 | 2.33 | 1h-indazole-3-carboxamide, n-[(1s)-1-(aminocarbonyl)-2-methylpropyl]-1-(5-fluoropentyl)- |
| M347T95_NEG | 205135.39 | 50470.06 | -2.02 | 0.23 | 1.56 | Anacardic acid |
| M389T114_NEG | 13504749.64 | 3143242.24 | -2.10 | 0.12 | 13.09 | Cholic acid |
| M473T27_NEG | 208768.32 | 48385.54 | -2.11 | 0.06 | 1.62 | Amastatin |
| M391T119_2_POS | 7014043.55 | 1582530.62 | -2.15 | 0.26 | 9.15 | 12-ketodeoxycholic acid |
| M432T162_NEG | 232824.04 | 52128.49 | -2.16 | 0.10 | 1.70 | Lithocholylglycine |
| M193T382_NEG | 226637.87 | 50559.19 | -2.16 | 0.09 | 1.69 | 2-keto-d-gluconic acid |
| M299T390_POS | 76810.37 | 17002.76 | -2.18 | 0.05 | 1.01 | Olomoucine |
| M341T388_NEG | 115722.45 | 24817.74 | -2.22 | 0.06 | 1.23 | D-Maltose |
| M309T119_POS | 958132.85 | 205457.71 | -2.22 | 0.13 | 3.49 | 4.alpha.-hydroxystanozolol |
| M337T116_POS | 664396.11 | 138671.70 | -2.26 | 0.23 | 2.88 | Prostaglandin b1 |
| M563T159_NEG | 100500.06 | 20773.25 | -2.27 | 0.23 | 1.12 | L-homoarginine |
| M365T413_POS | 302332.02 | 61219.92 | -2.30 | 0.11 | 1.98 | Cellobiose |
| M195T413_NEG | 141137.60 | 28213.36 | -2.32 | 0.04 | 1.38 | D-gluconate |
| M439T23_NEG | 268508.77 | 52932.53 | -2.34 | 0.06 | 1.89 | 5-hydroxy-l-tryptophan |
| M771T136_POS | 3657008.00 | 720298.29 | -2.34 | 0.11 | 6.65 | 1-hexadecyl-2-(8z,11z,14z-eicosatrienoyl)-sn-glycero-3-phosphocholine |
| M275T156_POS | 210337.21 | 40443.33 | -2.38 | 0.07 | 1.65 | 9-oxo-10e,12z,15z-octadecatrienoic acid |
| M506T174_2_POS | 2015781.49 | 377947.51 | -2.42 | 0.07 | 5.15 | 1-stearoyl-2-hydroxy-sn-glycero-3-phosphocholine |
| M464T243_2_NEG | 883369.96 | 164757.28 | -2.42 | 0.13 | 3.35 | Glycocholic acid |
| M373T175_POS | 157537.45 | 29187.27 | -2.43 | 0.10 | 1.46 | .beta.-muricholic acid |
| M448T200_2_NEG | 2058155.94 | 350879.03 | -2.55 | 0.13 | 5.09 | Glycodeoxycholic acid |
| M373T117_POS | 3561457.58 | 596517.80 | -2.58 | 0.19 | 6.91 | Ursocholic acid |
| M410T157_2_POS | 4092987.72 | 634807.58 | -2.69 | 0.09 | 7.59 | Ursodeoxycholic acid |
| M329T37_1_NEG | 250349.50 | 38589.38 | -2.70 | 0.12 | 1.82 | Quercetin 3,7-dimethyl ether |
| M215T156_POS | 202604.31 | 31063.07 | -2.71 | 0.04 | 1.71 | 5alpha-androstan-17beta-ol-3-one |
| M229T89_NEG | 316513.12 | 47735.49 | -2.73 | 0.06 | 2.10 | D-ribulose 5-phosphate |
| M417T47_POS | 161216.08 | 23859.28 | -2.76 | 0.08 | 1.52 | 3r-[[(4-fluorophenyl)sulfonyl]amino]-1,2,3,4-tetrahydro-9h-carbazole-9-propanoic acid |
| M347T33_NEG | 357515.82 | 52287.20 | -2.77 | 0.04 | 2.27 | Camptothecin |
| M193T414_NEG | 79465.05 | 10418.95 | -2.93 | 0.09 | 1.08 | D-galacturonic acid |
| M302T206_POS | 223577.77 | 28854.86 | -2.95 | 0.06 | 1.80 | Dobutamine |
| M391T149_2_NEG | 97025108.96 | 12397972.62 | -2.97 | 0.06 | 37.96 | Deoxycholic acid |
| M321T157_POS | 412313.50 | 48264.75 | -3.09 | 0.04 | 2.48 | 3.beta.-hydroxy-5-cholenoic acid |
| M221T385_NEG | 95770.25 | 10768.09 | -3.15 | 0.06 | 1.18 | Palatinose |
| M121T218_POS | 179889.30 | 19862.80 | -3.18 | 0.26 | 1.57 | Tyramine |
| M486T78_POS | 121787.34 | 12701.11 | -3.26 | 0.26 | 1.30 | Dibutyryl-cgmp |
| M169T30_NEG | 867462.54 | 86308.55 | -3.33 | 0.04 | 3.61 | Phloroglucinolcarboxylic acid |
| M209T46_1_POS | 83142.22 | 8264.07 | -3.33 | 0.10 | 1.10 | Trans-3,5-dimethoxy-4-hydroxycinnamaldehyde |
| M425T92_NEG | 1089083.03 | 105265.14 | -3.37 | 0.05 | 4.05 | L-cysteine-glutathione disulfide |
| M417T330_POS | 94242.05 | 8872.64 | -3.41 | 0.27 | 1.07 | Leu-Glu-Arg |
| M529T388_NEG | 311003.56 | 26948.72 | -3.53 | 0.12 | 2.05 | Harpagoside |
| M499T33_1_NEG | 1172287.99 | 94395.96 | -3.63 | 0.16 | 4.03 | Silydianin |
| M349T32_NEG | 1071335.21 | 85349.63 | -3.65 | 0.04 | 4.09 | Idarubicin |
| M241T111_NEG | 617544.97 | 49152.23 | -3.65 | 0.10 | 3.08 | D-glucose 6-phosphate |
| M519T78_NEG | 93753.02 | 7456.69 | -3.65 | 0.20 | 1.17 | [2-(3,4-dihydroxyphenyl)-5-hydroxy-7-methoxy-4-oxo-3-[(2s,3r,4r,5r,6s)-3,4,5-trihydroxy-6-methyloxan-2-yl]oxychromen-8-yl] acetate |
| M455T152_POS | 234307.35 | 18609.10 | -3.65 | 0.09 | 1.90 | 14z,17z,20z,23z,26z,29z-dotriacontahexaen-1-ol |
| M245T25_NEG | 639812.82 | 48006.38 | -3.74 | 0.28 | 3.02 | 3-Methoxy-4-Hydroxyphenylglycol Sulfate |
| M441T33_NEG | 181668.84 | 12266.58 | -3.89 | 0.06 | 1.68 | .alpha.-apooxytetracycline |
| M689T454_POS | 80714.34 | 5114.56 | -3.98 | 0.08 | 1.13 | Maltotetraose |
| M527T409_POS | 363072.61 | 22135.41 | -4.04 | 0.04 | 2.38 | Gentianose |
| M449T86_NEG | 338946.90 | 20366.94 | -4.06 | 0.25 | 2.22 | N-acetyl-p-fluoro-dl-phenylalanine |
| M327T32_NEG | 370982.45 | 20879.31 | -4.15 | 0.08 | 2.43 | 3-hydroxy-6,2',4'-trimethoxyflavone |
| M314T46_POS | 1405245.13 | 71421.08 | -4.30 | 0.15 | 4.66 | Feruloyl tyramine |
| M379T149_NEG | 337491.24 | 16421.75 | -4.36 | 0.16 | 2.27 | Daunorubicin |
| M363T85_NEG | 2313872.45 | 111455.20 | -4.38 | 0.25 | 5.83 | 1-methyluric acid |
| M273T92_NEG | 310117.21 | 14149.00 | -4.45 | 0.09 | 2.23 | Phenylbenzimidazolesulfonic acid |
| M465T127_NEG | 371631.51 | 15903.50 | -4.55 | 0.06 | 2.45 | Agnuside |
| M328T55_NEG | 279216.99 | 11760.94 | -4.57 | 0.23 | 2.05 | (e)-n-[2-hydroxy-2-(4-hydroxyphenyl)ethyl]-3-(4-hydroxy-3-methoxyphenyl)prop-2-enamide |
| M312T55_POS | 1723442.32 | 62957.48 | -4.77 | 0.16 | 5.17 | Icilin |
| M493T120_POS | 112027.60 | 2684.01 | -5.38 | 0.23 | 1.30 | 5-glc tricin |
