# Supplementary material for: Integrated omics analysis reveals the immunologic characteristics of cystic Peyer’s patches in the cecum of Bactrian camels
Source: PeerJ. 2023 Jan 9;11:e14647. doi: 10.7717/peerj.14647 (PMC9835693; doi:10.7717/peerj.14647)
Supplement: Table S5 — The permutation test is applied for Adnois and Anosim tests under a reduced model. Statistic R in Anosim represents a scaled contrast between the among- and within-group ranks. The R2 in Adonis represents the interpretation of differentiation between groups. P < 0.05 represented a significant difference between groups. [file peerj-11-14647-s005.docx]

Table S5. The significant difference between NPPS and PPS by the *Adnois* and *Anosim* tests

| Methods | Number of permutations | statistic R or R2 | *P*-value |
| --- | --- | --- | --- |
| *Adnois* | 719 | 1 | 0.1 |
| *Anosim* | 719 | 0.71489 | 0.1 |

The permutation test is applied for *Adnois* and *Anosim* tests under a reduced model.

Statistic R in *Anosim* represents a scaled contrast between the among- and within-group ranks.

The R2 in *Adonis* represents the interpretation of differentiation between groups.

*P* < 0.05 represented a significant difference between groups.
