# Supplementary figures and images for: Integrated omics analysis reveals the immunologic characteristics of cystic Peyer’s patches in the cecum of Bactrian camels
Source: PeerJ. 2023 Jan 9;11:e14647. doi: 10.7717/peerj.14647 (PMC9835693; doi:10.7717/peerj.14647)

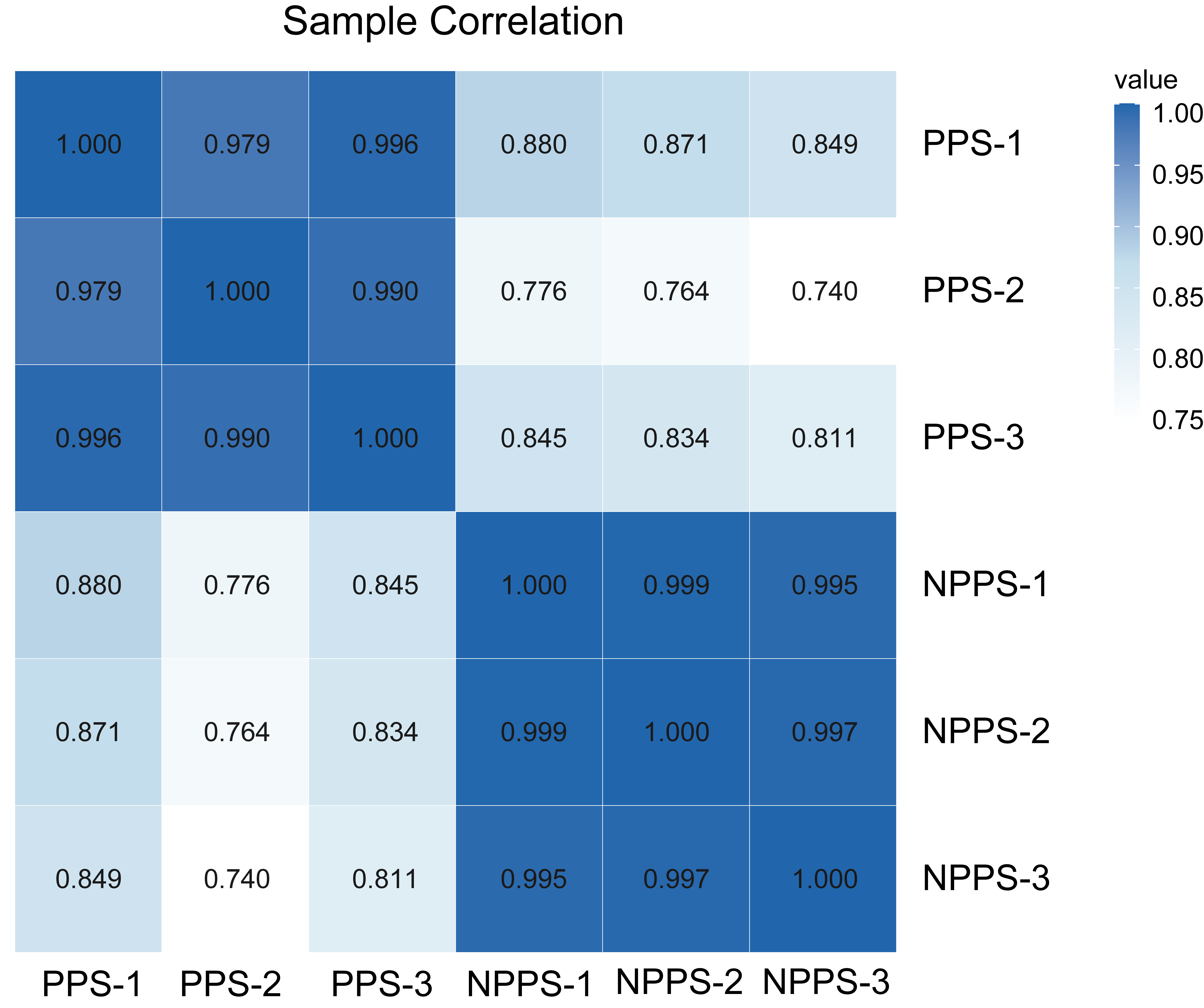

Supplement: Figure S1 [file peerj-11-14647-s006.png]

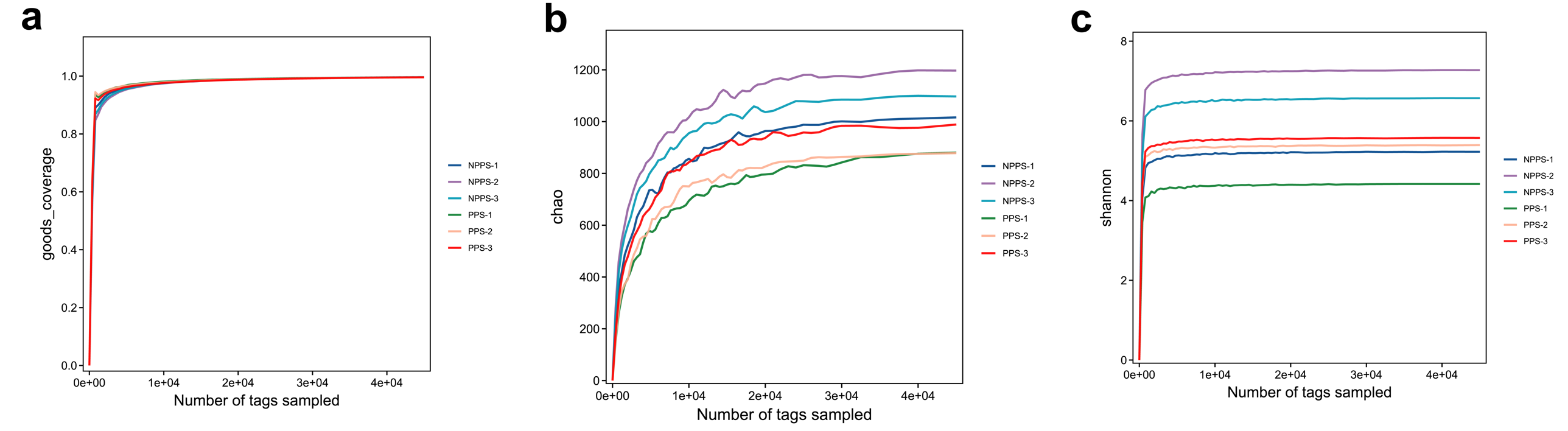

Supplement: Figure S2 [file peerj-11-14647-s007.png]

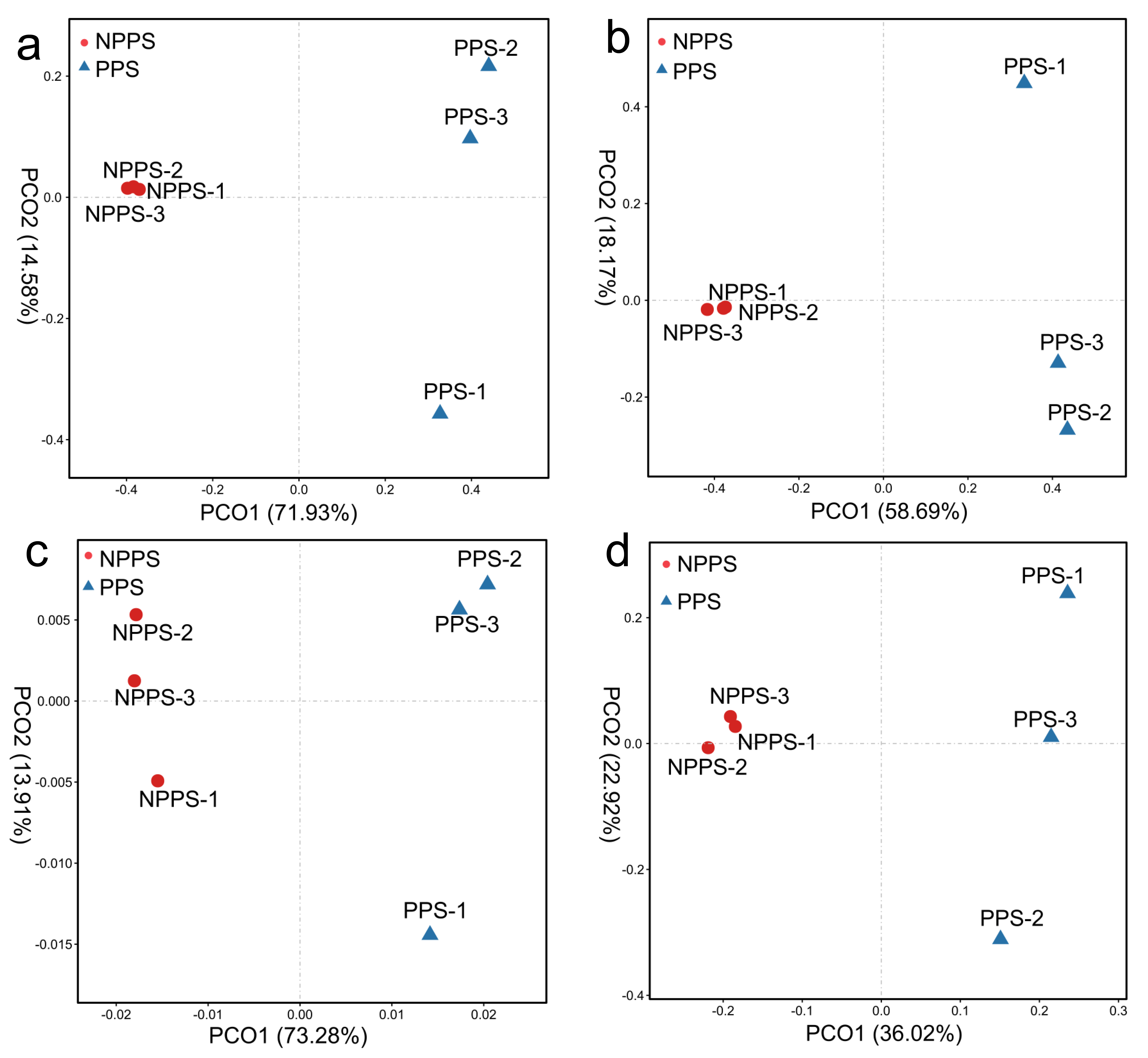

Supplement: Figure S3 — (A) Bray-Curtis PCoA, (B) Jaccard PCoA, (C) weighted UniFrac PCoA, and (D) unweighted UniFrac PCoA. [file peerj-11-14647-s008.png]

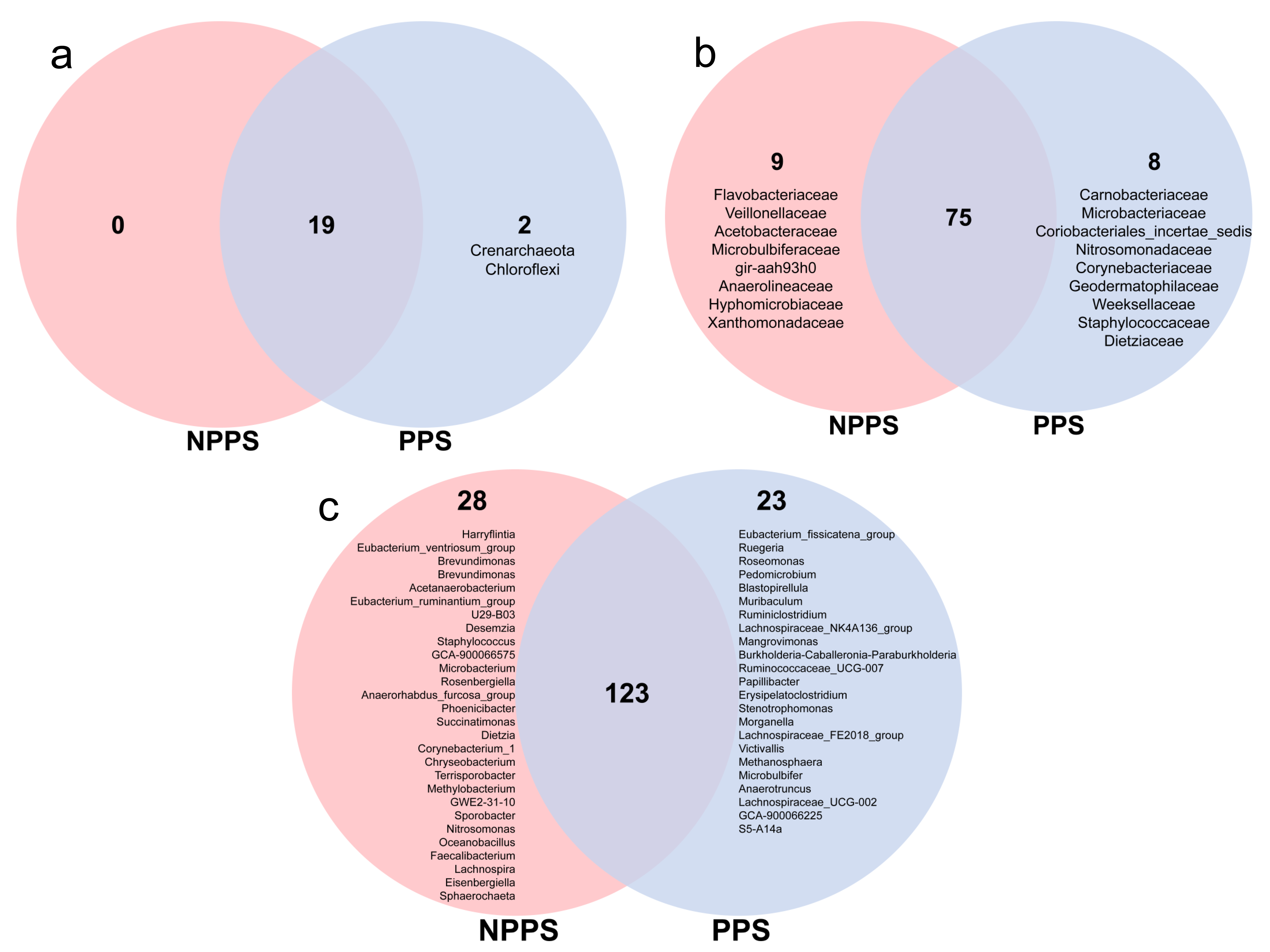

Supplement: Figure S5 — (A) based on the phylum level, (B) based on the family level, and (C) based on the genus level. [file peerj-11-14647-s010.png]
